# Instrument development, data collection, and characteristics of practices, staff, and measures in the Improving Quality of Care in Diabetes (iQuaD) Study

**DOI:** 10.1186/1748-5908-6-61

**Published:** 2011-06-09

**Authors:** Martin P Eccles, Susan Hrisos, Jill J Francis, Elaine Stamp, Marie Johnston, Gillian Hawthorne, Nick Steen, Jeremy M Grimshaw, Marko Elovainio, Justin Presseau, Margaret Hunter

**Affiliations:** 1 Institute of Health and Society, Newcastle University, Baddiley-Clark Building, Richardson Road, Newcastle upon Tyne, NE2 4AX, UK; 2 Health Services Research Unit, University of Aberdeen, Health Sciences Building, Foresterhill, Aberdeen, AB25 2ZD, UK; 3 College of Life Sciences and Medicine, University of Aberdeen, Health Sciences Building, Foresterhill, Aberdeen, AB25 2ZD, UK; 4 Diabetes Centre, Newcastle Primary Care Trust, Newcastle upon Tyne, UK; 5 Clinical Epidemiology Program, Ottawa Health Research Institute, Ottawa and Department of Medicine, University of Ottawa, Canada, 1053 Carling Avenue, Administration Building, Room 2-017, Ottawa ON, K1Y 4E9, Canada; 6 National Institute for Health and Welfare, Health Services Research Unit, PO Box 30, 00271 Helsinki, Finland

## Abstract

**Background:**

Type 2 diabetes is an increasingly prevalent chronic illness and an important cause of avoidable mortality. Patients are managed by the integrated activities of clinical and non-clinical members of primary care teams. This study aimed to: investigate theoretically-based organisational, team, and individual factors determining the multiple behaviours needed to manage diabetes; and identify multilevel determinants of different diabetes management behaviours and potential interventions to improve them. This paper describes the instrument development, study recruitment, characteristics of the study participating practices and their constituent healthcare professionals and administrative staff and reports descriptive analyses of the data collected.

**Methods:**

The study was a predictive study over a 12-month period. Practices (N = 99) were recruited from within the UK Medical Research Council General Practice Research Framework. We identified six behaviours chosen to cover a range of clinical activities (prescribing, non-prescribing), reflect decisions that were not necessarily straightforward (controlling blood pressure that was above target despite other drug treatment), and reflect recommended best practice as described by national guidelines. Practice attributes and a wide range of individually reported measures were assessed at baseline; measures of clinical outcome were collected over the ensuing 12 months, and a number of proxy measures of behaviour were collected at baseline and at 12 months. Data were collected by telephone interview, postal questionnaire (organisational and clinical) to practice staff, postal questionnaire to patients, and by computer data extraction query.

**Results:**

All 99 practices completed a telephone interview and responded to baseline questionnaires. The organisational questionnaire was completed by 931/1236 (75.3%) administrative staff, 423/529 (80.0%) primary care doctors, and 255/314 (81.2%) nurses. Clinical questionnaires were completed by 326/361 (90.3%) primary care doctors and 163/186 (87.6%) nurses. At a practice level, we achieved response rates of 100% from clinicians in 40 practices and > 80% from clinicians in 67 practices. All measures had satisfactory internal consistency (alpha coefficient range from 0.61 to 0.97; Pearson correlation coefficient (two item measures) 0.32 to 0.81); scores were generally consistent with good practice. Measures of behaviour showed relatively high rates of performance of the six behaviours, but with considerable variability within and across the behaviours and measures.

**Discussion:**

We have assembled an unparalleled data set from clinicians reporting on their cognitions in relation to the performance of six clinical behaviours involved in the management of people with one chronic disease (diabetes mellitus), using a range of organisational and individual level measures as well as information on the structure of the practice teams and across a large number of UK primary care practices. We would welcome approaches from other researchers to collaborate on the analysis of this data.

## Background

There is an enduring interest in healthcare in how best to predictably improve the quality of care received by patients. Different researchers approach this issue in different ways using different methods informed by a range of disciplinary backgrounds. Implementation science is the (usually multi-disciplinary) study of those factors that promote the uptake of the findings of clinical research into routine healthcare, thereby improving care for patients; it includes the study of both individual and organisational factors.

Within implementation science there has been increasing interest in the role of theoretical models to understand behaviours and identify techniques to change them. A systematic review of guideline implementation studies concluded that, by 1998, only 14 of 235 studies reported being inspired by or applying theories [1]. Since then there has been a steady increase in the number and type of studies testing or applying specific theories. Systematic reviews have quantified the empirical support for or predictive validity of social cognitive theories in predicting behaviour [2], diagnostic studies have explored a range of social cognitive, action and planning theories' prediction of intentions [3] and behaviour [4-6] and, using the theory of Planned Behaviour, have underpinned both intervention development [7] and process evaluation within randomised controlled trials [8,9]. Given the multiplicity of theories, authors have begun to offer various sorts of consolidated models that draw on multiple theories [10,11].

However, the reality of the efforts to explore these issues has been slower than anticipated due to factors such as the challenges of operationalising theories, the need to characterise clinical care in terms of its constituent behaviours, the challenges of measuring behaviour, and the tension between focussing on individuals *per se *or as constituent members of teams and organisations.

Our previous work focussed on 'relatively simple' clinical behaviours performed by individual healthcare professionals [4-6,12-16], but the majority of healthcare delivered, at least in primary care in high income countries, is for more complex behaviours involved in the management of chronic diseases.

Globally, type 2 diabetes is an increasingly prevalent chronic illness and is an important cause of avoidable mortality. Despite guidelines defining standards of care (*e.g*., http://guidance.nice.org.uk/CG/Published), there is evidence of less than optimum care in a number of areas [17]. Whilst some of the variability in care will reflect variation in patient physiology and behaviour, it will also reflect differences in the clinical management behaviours of individual clinicians and the organisations they work in. In the United Kingdom, patients are managed by the integrated activities of clinical and non-clinical members of primary care teams and therefore, whilst clinicians still perform individual clinical behaviours, process measures of care and patient outcomes reflect a complex mix of individual clinicians' behaviours (*e.g*., examining a patient's feet), sequential behaviours across clinicians (*e.g*., managing a patient's blood pressure, BP), and sequential behaviours across administrative and clinical staff (*e.g*., taking a blood sample to assess glycaemic control and then adjusting medication if appropriate).

The 'Improving The Delivery Of Care For Patients With Diabetes Through Understanding Optimised Team Work And Organisation In Primary Care' study-subsequently shortened to 'Improving Quality of Care in Diabetes (iQuaD)' Study (see study protocol for further detail [18])-aimed to investigate these issues. Designed as a predictive study (over 12 months), it aims to investigate organisational, team, and individual factors determining the multiple behaviours needed to manage diabetes and identified multilevel determinants of different diabetes management behaviours and potential interventions to improve them. This paper describes the instrument development, study recruitment, characteristics of the study participating practices and their constituent healthcare professionals and administrative staff, and reports the descriptive analyses of the data collected.

## Methods

### Study design and overview

The study was a predictive study over a 12-month period. In summary, practice attributes and a wide range of individually reported measures were measured at baseline; measures of clinical outcome were collected over the ensuing 12 months, and a number of proxy measures of behaviour were collected at 12 months (detailed in Table 1).

**Table 1:** Summary of variables, data collection methods and instruments, types and timings of data collected

Variables	Instrument	Data collected	Level and data source	Time period
Structural and functional characteristics of practices	Structured telephone interview	Practice demographics (*e.g*., staffing levels; skill mix) and functional characteristics (*e.g*., frequency and type of meetings held, staffing levels, staff responsibilities (both in general and in relation to diabetes); access to external services within primary and secondary care	Practice Practice manager, lead GP or nurse for diabetes	March to August 2008

Individuals' self-reported cognitions about their organisation	Baseline organisational postal questionnaire	Respondent demographics. Perceptions of: organisational justice, team climate, organisational citizenship and job control and demand, in general and (TCI, JCD) in relation to the provision of diabetes care, work stress, Plans to change employment, sickness absence, identification of key staff involved in provision of diabetes care.	Individual All practice staff	September to December 2008

Individuals' self-reported cognitions about six diabetes behaviours	Baseline clinical postal questionnaire	Theory based perceptions and beliefs in relation to performing the six target behaviours.	Individual Clinicians*	September to December 2008

**Behaviour data**				

Simulated behaviour	Baseline clinical postal questionnaire	Scores on four clinical scenarios	Individual Clinicians*	September to December 2008

Self-reported behaviour	12-month clinician postal questionnaire	Performance of the six target behaviours of interest over the 12 months since the baseline survey	Individual Clinicians*	September to December 2009

Patient physiological, biochemical, and drug data, and clinician diabetes management behaviours	Structured query of practice computer data	Patient physiological, biochemical and drug data and clinician diabetes management behaviours relating to the performance of the six target behaviours over the previous 12 months.	Practice Patients**	Conducted September to December 2009 Covers August 2007 to September 2009

Patient report of clinician behaviour	12 month patient postal questionnaire survey	Performance of four of the six target behaviours over the previous 12 months.	Practice Patients***	September to December 2009

QOF data	National database	Performance indicators for diabetes and primary care practice organisation	Patients**	May 2008 to April 2009

* Involved in care of patients with diabetes

** All patients in practice with type 2 diabetes

*** Random sample of patients with type 2 diabetes

At baseline we collected:

1. Structural and functional characteristics of the participating primary care practices;

2. Individuals' theory-based, self-reported cognitions about team functioning and practice organisational behaviour in their primary care practice (all staff);

3. Individuals theory-based, self-reported cognitions about performing the six clinical behaviours (clinicians only);

4. Simulated behaviour data using four clinical scenarios (clinicians only).

At 12 months we collected:

1. Self-reported performance of the six clinical behaviours (clinicians only)

2. Physiological, biochemical, and drug data and clinician diabetes management behaviours from practice computer systems on all patients with diabetes managed within the participating primary care practices

3. Patient report of clinician behaviour from a sample of patients with diabetes managed within the participating primary care practices

4. Quality and Outcome Framework data for the participating primary care practices

### Setting, recruitment, and participants

Practices were recruited from within the UK Medical Research Council General Practice Research Framework (MRC GPRF). When conducting similar previous studies with individually recruited primary care doctors [5], we had experienced low response rates in the face of long questionnaires. In order to be able to describe, characterise, and explore whole primary care practices, we wanted to achieve as close as possible to a 100% team response rate for the survey instruments from each practice. MRC GPRF practices volunteer to be research active and can directly receive funding to support their participation in research studies; practices were offered full reimbursement for the staff time taken to complete all study activities (including questionnaire completion) on condition that practice completion rates were satisfactory.

Recruitment was by postal invitation via the GPRF administration, with telephone follow-up of interested practices by the study research associate. Participants were all the clinical and non-clinical members of the primary care team in the practices recruited to the study.

### Clinical behaviours

To investigate the care offered to patients we identified six clinical behaviours (Table 2) performed in the management of patients with diabetes. These were chosen to: cover a range of clinical activities (prescribing, non-prescribing); reflect decisions that were not necessarily straightforward (controlling BP that was above target despite other drug treatment); and reflect recommended best practice as described by national guidelines [19]. The behaviours were precisely specified (according to the 'TACT' principle [20]: Target, Action, Context, Time or Who does What, Where and When) in order to provide consistency of measurement across practices and to reduce ambiguity when they were described to survey respondents.

**Table 2:** The six clinical behaviours

**1**.	**Giving advice about weight management **to patients with type 2 diabetes whose BMI is above a target of 30kg/m^2^, even following previous management.
**2**.	**Prescribing additional antihypertensive drugs **for patients with type 2 diabetes whose blood pressure (BP) is above a target of 140 mm Hg for Systolic BP or 80 mm Hg for Diastolic BP, even following previous management.

**3**.	**Examining foot circulation and sensation **in the feet of patients with type 2 diabetes, registered with your practice.

**4**.	**Providing advice about self-management **to patients with type 2 diabetes, registered with your practice.

**5**.	**Prescribing additional therapy for the management of glycaemic control **(HbA1c) for the management of HbA1c in patients whose HbA1c is higher than 8.0%, despite maximum dosage of two oral hypoglycaemic drugs.

**6**.	**Providing general education about diabetes **for patients with type 2 diabetes, registered with your practice.

### Instrument development and piloting

#### Telephone Interview schedule

A structured interview schedule was developed to collect details from a nominated study contact in each practice about practices' structures and functions (see Additional File 1) both in general and in relation to the provision of care for patients with type 2 diabetes. The content of the interview schedule was informed by previous studies [21,22], current recommendations for best practice (relating to the organisation of care for people with type 2 diabetes), and expert opinion. Minor amendments were made after the first two practice interviews.

### Baseline postal questionnaire

#### Questionnaire development

The baseline questionnaire consisted of three sections. The first section measured individuals' perceptions relating to team functioning and practice organisational behaviour, and was to be answered by all members of the practice. The second section covered cognitions about performing the six different clinical behaviours, and was to be answered by those members of the practice who provided care for patients with type 2 diabetes. The third section comprised four clinical scenarios relating to patients with type 2 diabetes, and was to be answered by the same group that answered the second section.

The questions covering individuals' perceptions relating to team functioning and practice organisational behaviour (Additional File 2, pages 1 to 8) comprised items based on theoretical constructs within Exchange Theory [23,24], and based on the premise that fair organisations produce well-functioning teams and good health outcomes for patients. The models were a number of existing validated scales: Organizational Justice Evaluation Scale [25,26], a shortened version of the Team Climate Inventory [27], Organisational Citizenship Behaviour [28], and the Job Content Questionnaire (JCQ) (measuring psychological job characteristics including job decision latitude and job demands [26]), (Table 3). Because high job strain, low organizational justice, and low team climate have all predicted a large variety of employee wellbeing and health outcomes, including psychological distress, low involvement, or low citizenship behaviour, these constructs were measured also as potential mediators of the clinical behaviours. Stress was measured using a 12-item measure based on the General Health Questionnaire (GHQ-12) [29]. In addition, 'diabetes specific' versions of two scales (shortened version of the Team Climate Inventory and the JCQ) were developed in order to explore if they were better predictors of these behaviours than their generic counterparts. These diabetes-specific versions were for completion only by respondents who provided care for patients with type 2 diabetes as part of their routine role within the practice. The questionnaire also included questions about demographic descriptors, the respondent's self-perceived role, who they identified as being involved in delivering care for patients with diabetes in the practice, and two questions covering sickness absence and plans to leave their current job.

**Table 3:** Description of the measures included in the organisational questions of the baseline questionnaire

Measure	Description (number of questions; scoring)
Organisational Justice	Measures perceived organisational justice and fairness (14; 1 to 7). Two dimensions: Procedural Justice (7); Relational Justice (7).

Team Climate Inventory*	Measures perceptions of openness to innovation in teams (14; 1 to 7). Four dimensions: Participation (4); Support for Innovation (3); Vision (4); Task Orientation (3)

Organisational Citizenship Behaviour	Measures 'extra role behaviours' within the team (13; 1 to 7)

Job Content Questionnaire*	Measures psychological job characteristics (13; 1 to 7). Two dimensions: Decision Latitude (9) and Job Demands (4). Decision Latitude is composed of two underlying dimensions: Skill discretion (6) and Decision Authority (3).

Stress measure	Negatively-worded items (6; 1 to 4) Positively-worded items (6; 1 to 4)

Self-reported sickness/illness absence	Free text item

Intention to leave	Free text item

* also included as a diabetes specific version

The second section of the baseline questionnaire (Additional File 2, pages 9 to 43) comprised items based on theoretical constructs from individual psychological models, including social cognitions models (Theory of Planned Behaviour [30], Social Cognitive Theory [31,32], Learning Theory [33,34], Self Reported Habit Index [35], Action Planning/Coping Planning [36,37]) (Table 4) asking about performing the six different clinical behaviours. The measured constructs from models of motivational factors (individual perceptions about, and attitudes towards, personally performing the six clinical behaviours and their intentions to perform the behaviours) and action factors (including habits, rewards, action plans, coping plans) over the following 12 months. The wording of the items to operationalise the theoretical models was informed by the pilot work undertaken for previous studies by the authors using similar methodology and theoretical models [4,5,12,38-40]. We measured intentions in two ways. As well as a traditional strength of intention measure (I intend/plan/expect to < perform behaviour >; score 1 to 7), a direct estimate of intention measure was included (Over the next 12 months, given 10 patients < definition of patients >, for how many do you intend to < perform behaviour >; score 0 to 10), in order to allow us to explore if one or other method of measurement affected the prediction of behaviour.

**Table 4:** Theories, models, and other measures of individual cognitions and attributes and example questions

Model, theoretical constructs (number of questions)	Example Item(s)
**Theory of Planned Behaviour (TPB)**	

Attitude (3)	In my management of patients with diabetes **I think it is beneficial to them to **'provide advice about weight management.' (scored 1 to 7)

Subjective Norm (2)	In my management of patients with diabetes **I am expected to **'provide advice about weight management.' (scored 1 to 7)

Perceived Behavioural Control (2)	In my management of patients with diabetes **I am confident that I can **'provide advice about weight management.' (scored 1 to 7)

Intention (3)	In my management of patients with diabetes **I intend to **'provide advice about weight management.' (scored 1 to 7)

Direct estimate of Intention (1)	Over the next 12 months, given 10 patients 'whose BMI is above target,' for how many do you intend to 'provide advice about weight management.' (Scored 0 to 10)

**Social Cognitive Theory (SCT)**	

Outcome expectancies (3)	In my management of patients with diabetes **I think it is good practice to **'provide advice about weight management.' (scored 1 to 7)

Self Efficacy: Clinical behaviour: 1 (10); 2 (9); 3 (8);(9); 5 (8); 6 (11)	I am confident that I can 'provide advice about weight management' to any patient whose BMI is above target even when 'the patient's BMI has been stable for five years.' (scored 1 to 7)

**Learning Theory (OLT)**	

Anticipated consequences (3)	In my management of patients with diabetes 'whose BMI is above target.'.. **overall, it is highly likely that they will be worse off if I **'provide advice about weight management.' (scored 1 to 7)

Evidence of habitual behaviour (2)	In my management of patients with diabetes 'whose BMI is above target.'.. **it is my usual practice to **'provide advice about weight management.' (scored 1 to 7)

**Self-reported Habit Index (SRHI) (12)**	Providing advice about weight management to patients whose BMI is above target is something that 'I do frequently.' (scored 1 to 7)

**Action planning/coping planning**	

Action planning (3)	I have a clear plan of 'how I will' 'provide advice about weight management.' (scored 1 to 7)

Coping planning: Clinical behaviour: 1 (10); 2 (9); 3 (4); 4 (9); 5 (8); 6 (11)	I have made a clear plan regarding 'providing advice about weight management to patients whose BMI is above target if ...' 'the patient's BMI has been stable for five years' (scored 1 to 7)

**Past behaviour (1)**	Over the past 12 months, for approximately how many of the last 10 patients with diabetes 'whose BMI was above target' did you 'provide advice about weight management' (scored 0 to 10).

**Demographics**	Gender, years qualified, trainer status, sessions worked per week; role within primary care practice; job title

The third section of the baseline questionnaire included four patient scenarios designed to simulate the behaviour that an individual clinician would perform during a consultation and delivered in a format to simulate the computer screen available during consultations (see pages 33 to 43 Additional File 2). Primary care doctors and nurses were asked whether they would address each of a series of diabetes-related factors, including the six behaviours targeted in the present study, by indicating whether they 'would do' or 'would do if time' address each diabetes-related area of care. The attributes of each scenario were varied, but given the small number of scenarios it was not possible to systematically vary every combination of every variable.

### Questionnaire piloting

Two primary care practices in northeast England took part in piloting the questionnaires. The first section (organisational questions) was piloted with seven administrative staff (practice managers, secretarial and reception staff) and seven healthcare professionals (primary care physicians, practice nurses, and one healthcare assistant). Piloting was by postal survey for all administrative staff and for five clinical staff. Participants were provided with the questionnaire and a stamped addressed envelope to return the questionnaire to the study research associate. They were given written guidance that asked them to complete the questions in their own time, noting how long it took to complete and to comment freely on the clarity and acceptability of the questions. The questions were found to be acceptable, there were no missing responses and the time taken to complete the instrument varied from seven to 25 minutes (median 20 minutes). No adjustments were made to the questions following piloting.

The second and third sections were initially piloted using postal methods as described above with one primary care physician and two practice nurses. One lead primary care physician for diabetes and one diabetes specialist nurse also piloted the questionnaire during a face-to-face session with the study research associate using 'think aloud' technique [41]. Based on the feedback received and concerns expressed during the 'think aloud' sessions, adjustments were made to minimise repetition in the wording of the items, and two behavioural scenarios (see Measures of behaviour below) were removed (leaving four in the final version) to shorten the questionnaire and to keep the completion time within an estimated maximum of two hours. The amended questionnaire was then re-piloted using postal methods with the two original 'think aloud' participants and an additional two primary care physicians and two practice nurses. No further amendments were suggested as a result of the re-piloting. All pilot participants received book vouchers (£10 for administrative staff, £20 for nursing staff, and £50 for doctors) for returning a completed questionnaire.

### Twelve-month self-reported behaviour questionnaire

A 'self-reported behaviour' questionnaire, asked individual clinicians about their performance of each of the six clinical behaviours over the previous 12 months (see Additional File 3: Self Reported behaviour questionnaire). The items used in this very brief questionnaire (one item for each of the six clinical behaviours) were worded: Over the past 12 months, given 10 patients with diabetes < attributes of patients >, for how many did you < perform behavior >? (scored 0 to 10). Such measures of behaviour are commonly used and are well predicted by social cognition models [2].

### Instrument administration

#### Telephone interview

Data were collected between March and August 2008 during a 30-minute telephone interview with a nominated study contact (practice manager, practice research nurse, or a general practitioner lead for diabetes) at each of the recruited primary care practices. The study contact was sent a summary of the data collected for verification and asked to check with practice colleagues as necessary if they were uncertain about the accuracy of the data provided.

### Baseline postal questionnaire survey

The baseline postal questionnaire survey ran between September and December 2008. All the questionnaires for a practice were delivered to the nominated study contact in the practice who then distributed the questionnaires to practice colleagues. All participants were provided with written information about the study, asked to complete their questionnaires individually, and provided with a pre-paid envelope to return their questionnaire directly to the study research associate. Reminders were sent to non-responders at two and four weeks. Individuals not wishing to complete the study questionnaire and who wanted this to be confidential from their practice colleagues were given the option of returning a blank questionnaire.

### Twelve-month self-reported behaviour questionnaire survey

This was administered 12 months after the baseline questionnaire and using the same method as described above.

### Measures of behaviour

Five different, complementary measures of the performance of the six study behaviours were collected. The first two provide individual level measures of behaviour, while the latter three give aggregated practice level behavioural data.

### Simulated behaviour

This 'simulated behaviour' measure derived from clinical scenarios (described above) provided the first of two measures of individual clinicians' self-reported performance of the six study behaviours. Clinicians could endorse that they 'would do' (score 2) or 'would do if time' (score 1) each behaviour plus add explanatory text. Scores for one of the simulated behaviours were adjusted to reflect current best practice-prescribing additional drug therapy for the management of HbA1c was, at the time of the study, advised for individuals whose HbA1c was above 8.0%. Therefore, for scenarios in which the simulated patient's HbA1c was ≤8.0%, the correct decision was not to prescribe additional therapy, and respondents who did not indicate that they would act on this were credited with having made the evidence-based decision.

### Clinician self-reported behaviour

The 12-month self-reported behaviour questionnaire (described above) provided the second measure of individual clinicians' self-reported performance of the six study behaviours.

### Clinician behaviour based on data extracted from practice computer systems

Anonymised individual patient biochemical, physiological, and drug data were extracted from practice computer systems for all patients with a diagnosis of type 2 diabetes registered with the practice (see Additional File 4: List of Read Codes for the data items). For each of the computer systems used by the practices, search queries were written by an experienced National Health Service (NHS) performance data manager. Data were extracted for a 25-month period (*i.e*., 12 months prior to and 12 months after the month within which the baseline survey was launched). The search queries were sent to each practice along with written guidance on running the query, a process that practices were familiar with. The performance data manager also provided practices with telephone and email support if needed.

### Patient-report of clinicians' behaviour

We anticipated that information on some of the study behaviours of interest might be recorded poorly, if at all, in the computer records, specifically those on the provision of advice on weight management, self-management, and general education. A single relevant question about each was included in a patient satisfaction questionnaire previously used by the Healthcare Commission [42]. In order to increase the specificity of the measure, as well as the single item, we identified additional items that assessed specific aspects of each behaviour with the aim of producing a composite score for each behaviour. We examined the internal consistencies and ran principle components analyses on the items within each behaviour and then across behaviours. Performance of foot examination was also asked about and so provided an additional, single item, measure of this behaviour.

Using a single posting, anonymous (to the research team) survey (for the questionnaire see Additional File 5), we asked patients in the study practices about their experiences of their clinicians providing advice about weight management, self-management, and general education about their diabetes. Aiming to achieve a final sample size of 25 respondents per practice, 86 practices approached 100 randomly selected patients anticipating a 25% response rate. Questionnaires were distributed from the practice and returned to the study research associate.

### Quality and outcomes framework data

The Quality and Outcomes Framework (QOF) is a voluntary annual reward and incentive programme for all primary care practices in UK, detailing practice performance across a number of clinical areas (of which diabetes mellitus is one) plus organisational areas [43,44]. The data are extracted from practice computer systems by the local primary healthcare administrative authority on an annual basis using a standard data extraction query. The data are publically available and QOF data on the diabetes and organisational domains were obtained from the NHS Information Centre http://www.ic.nhs.uk/. The QOF data for diabetes mellitus and practice organisation were collected for each of the participating practices for the 12-month period of QOF data collection (May 2008 to April 2009) that best matched the 12-month period after baseline questionnaire completion. Where available, practice level numerators and denominators were obtained for diabetes mellitus indicators and percentage achievement levels were calculated; where they were not available, the calculated point score is reported.

### Ethics approval

The study was approved by Newcastle and North Tyneside 2 Research Ethics Committee, REC reference number 07/H0907/102.

## Results

### Recruitment and instrument response rates

**Figure 1:**
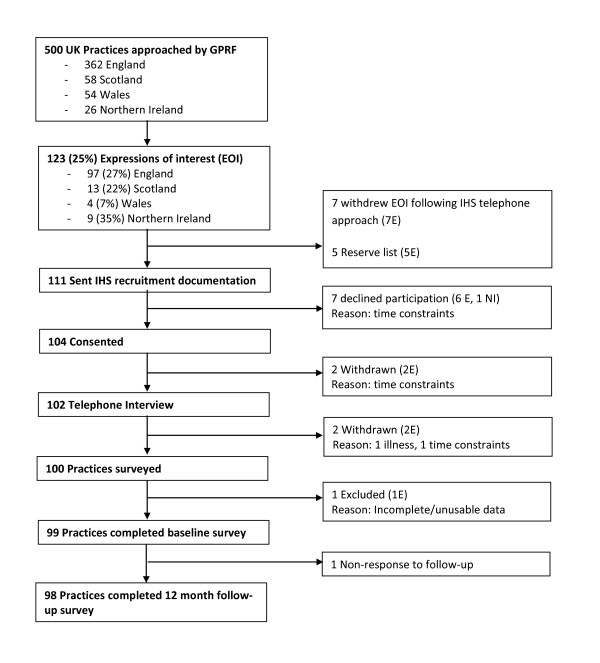
**Flowchart of the recruitment of primary care practices recruited to the iQuaD study**.

The process of recruitment of primary care practices is shown in Figure 1. The initial invitation went to all GPRF practices in Scotland, Wales, Northern Ireland, and a random sample of practices in England up to a total of 500 practices. One hundred practices were recruited and all took part in the telephone interview, baseline, and follow-up phases of the study. One practice was subsequently excluded from all analyses due to low completion rates for all data collection; we subsequently report on 99 practices. All practices completed a telephone interview. Informants were GPs for 47 practices, nurses for 37 practices and the practice manager for 15 practices. All practices were invited to verify their data summaries and 75 did so.

**Figure 2:**
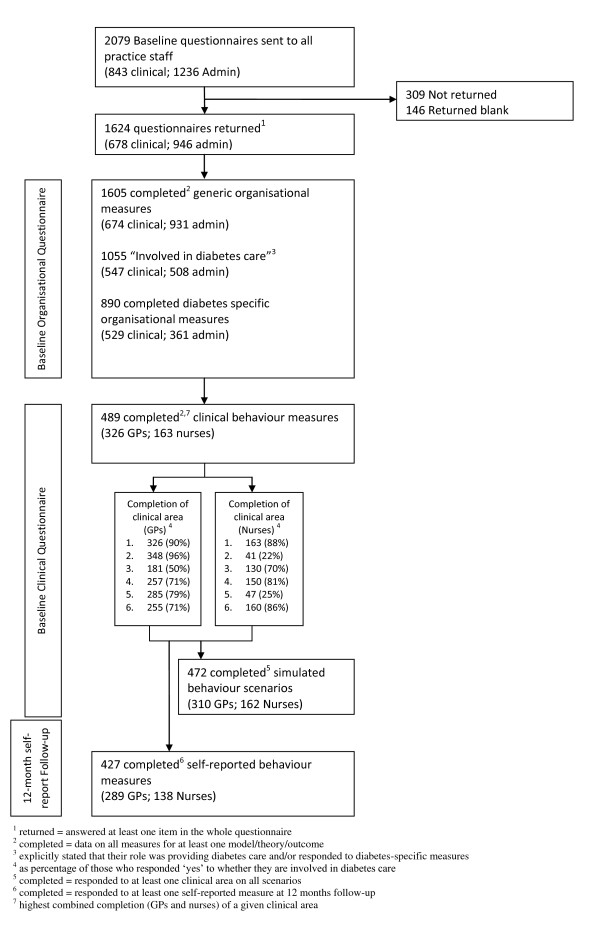
**Flowchart of individual clinicians and administrative staff from the 99 practices recruited to the iQuaD study**.

The baseline questionnaire (organisational questions) was sent to all clinical and administrative staff (2,079 in total). Usable completed questionnaires were returned by 946/1,236 (76.5%) administrative staff, 423/529 (80.0%) primary care doctors, and 255/314 (81.2%) nurses (see Figure 2). One thousand and fifty-five staff members indicated that providing care for patients with diabetes was part of their routine role and 890/1,055 (84.4%) went on to complete the diabetes-specific versions of the measures in the questionnaire.

The baseline questionnaire (clinical questions) was sent to all clinical staff within each of the 99 practices (843 in total). Of clinicians who indicated that they were involved in providing diabetes care, usable completed questionnaires were returned by 326/361 (90.3%) primary care doctors and 163/186 (87.6%) nurses (see Figure 2). Three hundred and ten primary care doctors and 162 primary care nurses responded to at least one area of care in every clinical scenario. Table 5 presents the practice level response rates for the two baseline questionnaires by staff type (excluding 146 questionnaires that were returned blank). We achieved 100% overall response rates from clinicians in 40 practices and achieved responses from over 80% of clinicians in 67 practices. We achieved 100% response from 38% of practices for at least one of the generic organisational questionnaires and from 84% of practice for at least one of the two diabetes-specific organisational questionnaires. Sixty percent of practices had a 100% response for questions on at least one individual-level psychological model.

**Table 5:** Individual level and practice level response rates

		Individual level response rate	Practice level response rates					
**Staff**	**Questionnaire**	**N (%)**	**100%**	**90-99%**	**80-89%**	**70-79%**	**50-69%**	**< 50%**

Overall	Any	1624/2079 (78.1)	18	18	32	8	16	7

Clinicians	Any	678/843 (80.4)	40	9	18	9	15	8
	
	Organisational (generic)	674/843 (80.0)	38	8	20	9	16	8
	
	Organisational (diabetes)	529/547 (96.7)	84	2	6	3	3	1
	
	Clinical	489/547 (89.4)	60	3	13	9	13	1

Admin	Any	946/1236 (76.5)	25	15	24	11	14	10
	
	Organisational (generic)	931/1236 (75.3)	22	13	26	10	18	10
	
	Organisational (diabetes)	361/508 (71.1)	27	1	12	21	25	13

The follow-up questionnaire was sent to 843 clinical staff. Six hundred and ninety-four (82.3%) completed questionnaires were returned. Of those involved in providing diabetes care, 427/547 (78.1%) could be paired with a completed baseline clinical questionnaire (see Figure 2).

Practices were supplied with a total of 8,600 patient questionnaires. Given the anonymous nature of the survey and the fact that practices with less than 100 patients with diabetes will have sent out fewer questionnaires a precise response rate cannot be calculated. A total of 3,591 analysable questionnaires were received (41.8% return rate).

### Study practices

Seventy-four of the recruited practices were located in England, 13 in Scotland, four in Wales, and eight in Northern Ireland. Thirty-seven were rural practices and 62 were urban; 15 had branch surgeries (range 2 to 5 sites); 18 were dispensing practices; 62 were training practices. The mean (SD) patient list size was 7,431 (4,040), with a mean (SD) proportion of patients aged over 65 years of 18% (7%). Most practices served patients of mainly 'White British' origin (84/99), and 63 practices 'never' or 'rarely' used interpreters. Tables 6 and 7 summarise the structural and functional characteristics of the study practices, both in general and in relation to diabetes care. There was a mean (SD) of 5.4 (2.7) doctors per practice covering a mean (SD) of 36.4 (20) half-day (notionally 3.5 hour) sessions and providing a mean (SD) of 515 (315) appointments per week. Similarly 3.1 (1.6) nurses per practice offered 17.7 (10.5) half-day sessions. Though only compared descriptively, study practices were of an equivalent size to MRC GPRF practices overall (mean list size 7,696). Since devolution in 1998, comparative UK data is hard to find but, compared to all general practices in England, the study practices were larger and had more doctors (2007 England mean list size: 6,487; mean number of practitioners per partnership: 4) and, at 4%, the study sample also contained a low proportion of single-handed practices [45].

**Table 6:** Summary data of the general functional and structural characteristics of the practices

Functional Characteristics	Staff levels (mean (SD))
Primary care doctors	5.4 (2.7); Partners 4.2 (2.2); sessions covered 36.4 (20.0); appointments per week 515 (345)

Primary care nurses	3.1 (1.6); sessions covered 17.7 (10.5)

At least one GP or nurse with diploma training	26 have both GP and a nurse; 8 have only a GP; 15 have only a nurse; 23 have neither a GP nor a nurse; 27 not reported

Healthcare Assistants	1.1 (0.9); sessions covered 7.1 (8.8)

Number of reception/administrative staff	11.7 (6.7)

**Staff turnover**	

Clinical staff (GPs and Nurses)	15 practices reported turnover of up to two clinical staff members in the previous twelve months. In all practices these had been replaced.

Admin staff (all clerical and admin)	61 practices reported turnover of up to two admin staff members in the previous twelve months. In all but 5 practices these had been replaced.

**Meetings**	

Practice	Held by 83 practices; monthly* for 1.5 hours; majority (52) include all practice staff

Partner	Held by 75 practices; monthly* for 1.5 hours; 27 GPs only; 48 included other staff, but most frequent combination was partners and practice manager (36).

Clinical meetings	Held by 71 practices; monthly* for one hour; 44 exclusively for clinical staff; 27 included non-clinical staff

Administrative meetings	Held by 66 practices; quarterly* for one hour; 66 include all admin staff.

Educational meetings	Held by 83 practices; 39 at least monthly and 36 at least quarterly, remainder bi-annual or annual, duration varied from one hour to protected half-day sessions: 44, all staff attend; 33, clinical staff only

*median frequency

**Table 7:** Summary data of the (Type 2) diabetes related functional and structural characteristics of the practices

Structure of care provision	N, frequency/service provider
**Dedicated diabetes clinic**	71 practices

Frequency; duration	43, weekly; 14, monthly; 14, n/r*; 1 to 2 half-day sessions

Appointment length	Most frequently 20 to 30 mins

Who leads management?	16, doctor; 49, nurse; 6, co-managed by doctor and nurse

Admin support	29, dedicated member of admin team; 37, general admin team, 1, none; 4, n/r

Doctor available (if required) at clinic	69, diabetes lead doctor; 30, Patient's own or duty doctor

Other staff available at clinic	9, Diabetes specialist nurse; 16, dietician

**Seen in routine appointments**	28 practices

Appointment length	Most frequently 20 ro 30mins

Who leads management?	8, doctor; 19, nurse; 1, co-managed by doctor and nurse

Admin support	11, dedicated member of admin team; 13, general admin team' 4, n/r

**General management of patients**	

Routine recall interval	61, annual review; 34, 6-month review; 4, 3-month review

Who organizes recall?	58, admin support; 36, nurse; 5, GP

Blood tests	77, done in advance; 22, done on day of visit

Patient sees doctor routinely at review	43, always for Annual review; 56, only 'if indicated' for any review

Insulin initiation	50, in-house (16 by doctor, 26 by practice nurse, 6 by DSN**; 2, n/r); 49, in Secondary Care only

Patients on insulin managed in practice	60, yes, only if stable on insulin; 39, secondary care only

Foot inspection	58, in-house; 17, referred to podiatry services; 24, not reported

Use of guidelines for diabetes	53, both national (most frequently NICE***) and local guidelines; 33, national guidelines only; 9, local guidelines only; 4, do not use guidelines

**Patient education**	

Availability of Structured Patient Education Programme	25, secondary care; 37, primary care; 4, location not specified. 33, no structured programme available

Practice provision of patient education	26, provide 'in-house' education only; 73, refer patients for external education: 36, 'structured programme' (most commonly DESMOND); 37, refer to locally developed educational sessions.

Who provides in-house education	75, nurse-led; 5, doctor-led; 19, shared

Materials	55, use in-house leaflets; 68, use DUK**** leaflets; 11, use PCT leaflets. 39, refer patients to DUK website; 5, refer patients to local website; 6, refer patients to in-house website

**Management aids**	

Diaries	67, use patient diaries; 20, do not use diaries;12, n/r

Blood testing kits	40, use with all patients/patients who request kits; 20, use only with patients on insulin; 9, do not use; 24, n/r

Urine testing kits	21, use with all patients/patients who request kits; 5, use only with patients on insulin; 41, do not use; 32, n/r

**Access to specialist support services outside of the practice**	

Diabetes Specialist Nurse	53, via secondary care; 28, primary care; 18, n/a*****

GPwSI (in Diabetes)	6, via secondary care; 14, primary care; 79, n/a

Dietician	40, via secondary care; 17, primary care; 42, n/a

Podiatrist	32, via secondary care; 30, primary care; 37, n/a

Retinal Screening	29, via secondary care; 36, primary care; 34, n/a

Diabetes Centre in Secondary Care	23, available to consult for advice

Specialist Diabetologist	44, available to consult for advice

* n/r not reported; **Diabetes Specialist Nurse; ***National Institute of Health and Clinical Excellence; **** Diabetes UK; ***** n/a not available

### Questionnaire results descriptive data

#### Baseline organisational questionnaire

Table 8 presents alphas for internal consistency of the measures included in the organisational questionnaire and the mean (SD) scores for each measure and for both general and diabetes specific organisational measures. The internal consistencies were all acceptable, with alpha coefficients ranging from 0.61 to 0.97 and Pearson correlation coefficient (used for two item measures) from 0.32 to 0.81. Although the Team Climate Inventory has not been widely used in UK primary care [46], the scores are very similar to those from a recent UK study which reported values from 14 practices in South Tyneside [47]. For scores on constructs in the Job Control Model, the internal consistencies ranged from 0.61 to 0.78, compatible with the range of previously reported values (0.68 to 0.82) [48]. The diabetes specific versions of these two measures were scored very similarly. Scores across the other scales were well into the positive range of responses; for measures on a 1 to 7 scale the median (inter-quartile range) score was 5.32 (5.28 to 5.58). Table 8 also shows rates self-reported episodes and days of sickness and intention to leave. Sickness rates were low (mean number of days lost per year was just over two) and highly skewed with a small number of respondents reporting higher rates of sickness. The table also includes intention to leave with just over 8% of staff reported intending to leave.

**Table 8:** Internal consistency means and standard deviations of scores for team function and organisational behaviour measures, for general and diabetes specific measures and illness sickness absence and intention to leave

			GPs and nurses			Administrative staff		
**Constructs**	**Dimensions**	**N (items)**	**N**	**Internal consistency^1^**	**Mean (SD)**	**N**	**Internal consistency^1^**	**Mean (SD)**

Organisational Justice	Procedural Justice	7	668	0.93	5.25 (0.92)	924	0.96	5.30 (1.13)
	Relational Justice	7	672	0.92	5.80 (0.81)	923	0.95	5.30 (1.10)

Team Climate (TCI)	Participation	4	677	0.92	5.73 (1.07)	940	0.93	5.28 (1.21)
(Generic)	Support for Innovation	3	675	0.88	5.30 (1.07)	937	0.93	5.17 (1.22)
	Vision	4	675	0.86	5.63 (0.78)	920	0.93	5.30 (1.14)
	Task Orientation	3	675	0.87	5.33 (1.01)	930	0.89	5.15 (1.15)

Team Climate (TCI)	Participation	4	533	0.92	5.62 (1.03)	379	0.94	5.40 (1.14)
(Diabetes-specific)	Support for Innovation	3	533	0.92	5.23 (1.14)	379	0.95	5.38 (1.17)
	Vision	4	532	0.84	5.67 (0.81)	360	0.94	5.48 (1.07)
	Task Orientation	3	532	0.89	5.28 (1.03)	358	0.91	5.22 (1.19)

Organisational Citizenship Behaviour		13	671	0.91	5.61 (0.80)	926	0.92	5.40 (0.93)

Job content Questionnaire	Decision Latitude	9	674	0.73	99.01 (10.79)	933	0.78	82.28 (15.85)
(Generic)	Skill Discretion	6	674	0.61	48.76 (4.87)	933	0.67	39.14 (7.55)
	Decision Authority	3	674	0.70	50.24 (7.61)	933	0.76	43.14 (10.55)
	Job Demands	4	674	0.73	44.59 (8.14)	933	0.70	42.66 (8.24)

Job content Questionnaire	Decisional Latitude	9	529	0.77	94.85 (12.27)	361	0.78	75.82 (16.55)
(Diabetes-specific)	Skill Discretion	6	529	0.68	46.73 (5.68)	361	0.71	37.31 (8.25)
	Decision Authority	3	529	0.69	48.12 (8.40)	361	0.68	38.51 (10.67)
	Job Demands	4	529	0.75	42.36 (8.56)	361	0.71	39.31 (9.22)

Stress (negative items)		6	663	0.83	1.96 (0.41)	912	0.83	1.95 (0.48)
Stress (positive items)		6	662	0.81	2.12 (0.36)	926	0.77	2.14 (0.38)

Self-reported sickness/illness	Episodes (mean (range))	1	651	n/a	0.55 (0; 6)	858	n/a	0.80 (0; 6)
	Days (mean (range))	1	632	n/a	2.16 (0; 60)	823	n/a	2.62 (0; 62)

Intention to leave	% responding 'yes'	1	662	n/a	8.16%	889	n/a	8.77%

All scales scored 1 to 7 except Stress which is scored 1 to 4 (Much less than usual, Same as usual, More than usual, Much more than usual) and JCQ recoded from 1 to 7 to 1 to 5.

### Baseline clinical questionnaire

Table 9 presents the mean (SD) scores and internal consistency for each theoretical construct included in the clinical questionnaire. The internal reliability measures are all acceptable. Across the six behaviours, the scores for the constructs were all generally well towards the positive end of the seven point scoring scale. For each of the theories the median (range across behaviours) was:

**Table 9:** Internal consistency, means and standard deviations of scores for predictive clinical measures, by theoretical and conceptual model, for each of the six clinical behaviours

		Behaviour 1: Providing weight management advice			Behaviour 2: Prescribing additional antihypertensive drugs			Behaviour 3: Examining feet (circulation)^2^		
**Model**	**Constructs**	**N items**	**Internal consistency**^**1**^	**Mean (SD)**	**N items**	**Internal consistency**^**1**^	**Mean (SD)**	**N items**	**Internal consistency**^**1**^	**Mean (SD)**

TPB	Attitude	3	0.72	6.27 (0.78)	3	0.95	5.71 (1.04)	3	0.70	6.13 (1.01)
	Subjective norm	2	0.42	5.92 (0.98)	2	0.59	5.56 (1.09)	2	0.69	5.61 (1.51)
	PBC	2	0.41	5.06 (1.12)	2	0.33	5.22 (1.06)	2	0.32	5.62 (1.10)
	Intention strength	3	0.87	6.08 (0.86)	3	0.93	5.46 (1.09)	3	0.97	5.56 (1.67)
	Direct estimation of intention	1	n/a	9.00 (1.82)	1	n/a	7.68 (2.11)	1	n/a	7.36 (3.44)

SCT	Outcome expectancies	3	0.72	6.27 (0.78)	3	0.95	5.71 (1.04)	3	0.70	6.13 (1.01)
	Self-efficacy	10	0.92	4.95 (1.10)	9	0.92	4.63 (1.13)	4	0.90	5.73 (1.28)

LT	Anticipated consequences	2	0.40	6.26 (0.98)	2	0.52	5.77 (1.20)	2	0.37	6.50 (0.85)
	Evidence of habit	2	0.69	5.94 (1.00)	2	0.50	5.41 (1.17)	2	0.81	5.46 (1.69)

n/a	Self-reported habit index	12	0.93	4.82 (1.11)	12	0.94	4.25 (1.21)	12	0.96	4.57 (1.57)

n/a	Past behaviour	1	n/a	7.79 (2.12)	1	n/a	6.39 (2.11)	1	n/a	6.73 (3.35)

Plans	Action planning	3	0.92	5.88 (0.92)	3	0.94	5.91 (0.84)	4	0.94	6.22 (0.99)
	Coping planning	10	0.96	4.45 (1.26)	9	0.95	4.61 (1.22)	4	0.97	5.53 (1.48)

		**Behaviour 4: Providing advice on self-management**			**Behaviour 5: Prescribing additional therapy for managing glycaemic control**			**Behaviour 6: Providing general education**		

**Model**	**Constructs**	**N items**	**Internal consistency**^**1**^	**Mean (SD)**	**N items**	**Internal consistency**^**1**^	**Mean (SD)**	**N items**	**Internal consistency**^**1**^	**Mean (SD)**

TPB	Attitude	3	0.88	6.29 (0.82)	3	0.93	6.00 (0.79)	3	0.80	6.37 (0.75)
	Subjective norm	2	0.56	5.77 (1.07)	2	0.47	5.69 (0.94)	2	0.57	5.82 (1.08)
	PBC	2	0.50	5.29 (1.14)	2	0.36	5.24 (1.07)	2	0.49	5.41 (1.12)
TPB	Intention strength	3	0.93	5.73 (1.17)	3	0.88	5.57 (0.94)	3	0.94	5.92 (1.03)
TPB	Direct estimation of intention	1	n/a	8.16 (2.35)	1	n/a	7.89 (1.97)	1	n/a	8.56 (2.03)

SCT	Outcome expectancies	3	0.88	6.29 (0.82)	3	0.93	6.00 (0.79)	3	0.80	6.37 (0.75)
	Self-efficacy	9	0.92	5.38 (1.05)	8	0.92	5.04 (1.10)	11	0.92	4.79 (1.09)

LT	Anticipated consequences	2	0.42	6.24 (1.02)	2	0.57	6.03 (1.09)	2	0.54	6.32 (1.11)
	Evidence of habit	2	0.81	5.67 (1.21)	2	0.66	5.61 (1.01)	2	0.81	5.86 (1.14)

n/a	Self-reported habit index	12	0.96	4.98 (1.32)	12	0.95	4.42 (1.25)	12	0.96	5.03 (1.30)

n/a	Past behaviour	1	n/a	7.72 (2.42)	1	n/a	6.87 (2.24)	1	n/a	7.93 (2.36)

Plans	Action planning	3	0.96	5.44 (1.16)	3	0.97	5.62 (1.08)	3	0.97	5.58 (1.17)
	Coping planning	9	0.96	4.71 (1.36)	8	0.96	4.76 (1.31)	11	0.96	4.49 (1.26)

TPB Theory of Planned Behaviour, SCT Social Cognitive Theory, LT Learning Theory

All items scored 1 to 7 except for Direct estimation of intention and past behaviour which were scored 1 to 10.

^1^Cronbach's alpha for measures with > 2 items. Pearson correlations for measures with 2 items.

^2^Two sets of four self-efficacy items were used to assess self-efficacy to examine the circulation and sensation of feet separately. Internal consistency for the items measuring sensation was 0.91, mean = 5.69, SD = 1.32

• Theory of Planned Behaviour: Attitude 6.2 (5.7, 6.4), Subjective Norm 5.7 (5.6, 5.9), Perceived Behavioural Control 5.3 (5.1, 5.6), Intention Strength 5.7 (5.5, 6.1), Intention (direct estimation, 0-10) 8.0 (7.4, 9.0).

• Social Cognitive Theory: Outcome Expectancies 6.2 (5.7, 6.4), Self-Efficacy 5.0 (4.6, 5.7), Proximal Goals 5.7 (5.5, 6.1).

• Learning Theory: Anticipated Consequences 6.3 (5.8, 6.5), Evidence of habitual behaviour 5.6 (5.4, 5.9).

• Action Planning 5.8 (5.4, 6.2), Coping Planning 4.7 (4.5, 5.5).

Within the theories, whilst overall no Theory of Planned Behaviour construct was scored below five, the control item had the lowest scores across all six behaviours, a similar pattern to the self-efficacy item scores within Social Cognitive Theory suggested that clinicians had stronger motivational than action cognitions. Coping planning was scored lower than action planning for all six behaviours, suggesting that clinicians were clearer how to initiate behaviours than to cope with problems should their initial plans not succeed.

Intention (measured either as strength of intention or direct estimation) to perform the behaviour was highest for 'giving advice about weight management' and was lowest for 'prescribing additional anti-hypertensive drugs' (strength of intention) and 'foot examination' (direct estimation). The highest habit score was also for 'giving advice about weight management' and the lowest was for 'prescribing additional anti-hypertensive drugs.' For action planning and coping planning the highest scores were both for 'foot examination'; the lowest action planning score was for 'giving advice about self-management' and the lowest coping plan score for 'giving advice about weight management.'

### Measures of behaviour

#### Behaviour simulation

The proportion of clinicians reporting that they 'would do' or 'would do if time' each behaviour by scenario is shown in Table 10. Across the scenarios, there was no behaviour that all clinicians felt should be performed; for doctors, the scores ranged from 22% (scenario 3; prescribing additional therapy for the management of glycaemic control) to 89% (scenario 1; prescribing additional anti-hypertensive drugs), whilst for nurses the scores ranged from 18% (scenario 3; prescribing additional therapy for the management of glycaemic control) to 79% (scenario 1; giving advice about weight management).

**Table 10:** Measures of clinicians' behaviour

			Behaviour					
**Measure of behaviour**			**Provide advice about weight management**	**Prescribing for the management of HbA1c**	**Inspect feet**	**Provide advice about self-management**	**Prescribing additional antihypertensive drugs**	**Provide general patient education**

Behaviour simulation scenarios^# ^% (n) would do or would do if time	GPs	Scenario 1	77% (279)	36% (131)	63% (229)	54% (195)	89% (320)	61% (219)
	Nurses		79% (147)	22% (40)	70% (130)	67% (125)	76% (141)	66% (123)
	
	GPs	Scenario 2	77% (276)	85% (305)	58% (210)	53% (190)	46% (167)	63% (228)
	Nurses		75% (140)	68% (127)	68% (126)	66% (122)	51% (95)	70% (130)
	
	GPs	Scenario 3	68% (246)	22% (78)	52% (188)	41% (149)	81% (294)	53% (191)
	Nurses		70% (130)	18% (34)	67% (124)	60% (112)	65% (121)	62% (115)
	
	GPs	Scenario 4	68% (246)	84% (302)	51% (183)	45% (163)	72% (260)	61% (221)
	Nurses		71% (132)	65% (120)	61% (113)	58% (108)	62% (116)	68% (127)

12-month self report ^##^	GPs	Mean (SD)	7.56 (2.20)	6.93 (2.50)	5.40 (3.47)	7.24 (2.45)	6.68 (2.38)	7.40 (2.44)
	Nurses	Mean (SD)	9.03 (1.91)	7.96 (2.09)	9.16 (1.89)	8.90 (2.03)	5.91 (3.15)	8.86 (2.20)

Patient report		% (n) (single item)	51% (1716)^1^	n/a	91% (3078)^2^	68% (2292)^3^	n/a	73% (2443)^4^

Patient report		N items Mean (SD) (composite)	8 2.50 (2.25)	n/a	n/a	3 1.51 (0.99)	n/a	18 7.44 (5.16)

Practice computer data			81.3% (23864/29362) patients with record weight or BMI Mean BMI 30.74 (95% CI: 30.67, 38.83)	58.9% (624/1059) of eligible patients prescribed an additional therapy	77.1% (22640/29362) with record of foot exam	n/a	39.5% (1595/4038) patients prescribed an additional therapy	n/a

^# ^For behaviour simulation, the denominator for GPs was 361 and for nurses, 186.

^## ^Possible score 0-10.

^1 ^Responded 'Yes' to the question 'Thinking about the last 12 months, when you received care for your diabetes from a doctor or nurse were you given advice about how to manage your weight?'

^2 ^Responded 'Yes' to the question 'In the last 12 months have you had your bare feet examined?

^3 ^Same stem as 1; Responded 'Yes' to the question 'were you given advice about how YOU should manage YOUR diabetes?'

^4 ^Same stem as 1; Responded 'Yes' to the question 'were you provided with general information about diabetes?'

### Clinician self-reported behaviour questionnaire and patient report of clinician behaviour

The mean (SD) rates of performance of the six behaviours are shown in Table 10 along with the patient responses to the questions about the three receiving advice behaviours and foot examination. Within the self-report questions, for both groups of clinicians, although reported rates of performing the behaviours were high, with two-thirds of rates being above seven out of ten, there was variation within the rates with standard deviations generally being just over two. Nurses reported performing the three 'giving advice' behaviours more often than doctors did, reporting performing the behaviour for almost 9 out of 10 patients. For foot examination, there was the widest difference between doctors and nurses, potentially reflecting different agreed roles and different patient populations seen.

The single-item patient report data are directly comparable to the clinician report data and, for foot examination, the patients' reports matched the nurses self-report almost exactly. For the other three advising behaviours, the patient-reported rates of receiving advice are consistently lower than the clinician-reported rates of giving it. For advice about self-management and providing advice about general education (converting the clinician n/10 scores into percentages) the gap is 21% and 14%, respectively. For advice about weight management, the gap is 52% with clinicians reporting that they gave advice about twice as often as patients reported receiving it.

When testing the composite items, the principal components analysis (PCA) on items within each behaviour suggested that each involved more than one component. For providing weight management advice and providing general education, these did not outweigh the clinical face validity of the initial scales nor did removing items improve the internal consistency. For providing self-management advice, PCA results informed the decision to remove three items. For the resulting composite measures, there were eight items for providing weight management advice (Cronbach's alpha 0.80), three items for providing self-management advice (Cronbach's alpha 0.66), and 18 items for providing general education (Cronbach's alpha 0.91). Details of the items and the analysis are in Additional File 6.

The mean (SD) scores for the composite items are shown in Table 10. For providing weight management advice, 51% of patients endorsed the single item but the mean number of items endorsed was 2.5/8, although 71% responded 'yes' to at least one item. Similarly, for providing self-management advice, 67.5% of patients endorsed the single item, the mean score on the composite measure was 1.5/3 and 83.4% responded 'yes' to at least one item; for providing general education, 72.3% endorsed the single item, the mean score on the composite measure was 7.4/18 and 93% responded 'yes' to at least one item.

### Clinician behaviour based on data extracted from practice computer systems

#### Running the query

Of the 99 included practices, one refused to run the data extraction query because of previous problems when running computer data extraction queries. For seven practices operating one computer system the query did not work, and four practices did not run the query despite repeated reminders. Thus 87 of the 99 practices ran the electronic query. For four of the practices, there was no usable drug data; the issuing of prescriptions was recorded but not the drug name or dose. A fifth practice had many missing data items for the second year-no patients in that practice were found as being eligible for the addition of an extra therapy to control their HbA1c and there were no recorded feet inspections in year two (although there were many recorded in year one). A sixth practice had no eligible patients for the glycaemic control behaviour. Therefore the analyses of behaviour two (prescribing additional antihypertensive drugs) and behaviour five (prescribing additional therapy for managing glycaemic control) are based on 83 and 81 practices, respectively, with 86 practices being analysed for behaviour three (examining feet).

### Computer data and the study behaviours

The rates of the study behaviours are in Table 10. The data extracted from the practice computers are usually of the form of process (recording that a behaviour was done such as issuing a prescription) or intermediate patient outcome measures (such as recorded BP). The links between this data and the study behaviours are more or less direct. For behaviour one (providing advice on weight management), data for weight/height/body mass index (BMI) was available from all practices and reflects the physiological endpoint of the behaviour we asked about. However, assuming such advice is given, there are a number of clinician (how well was it given) and patient (was it heard, accepted, acted upon) factors that intervene before any effect of performing the behaviour plays out through a change in a measure such as BMI. Unfortunately, the available computer codes for offering advice about weight though present were infrequently used and hence cannot be used as an outcome measure in this project. Behaviour two (prescribing additional antihypertensive drugs) and behaviour five (prescribing additional therapy for managing glycaemic control) relate to drug prescription in relation to physical examination or laboratory test results. Values for BP and HbA1c were universally available, and drug data that was available from 81 practices. The analysis is currently computing the eligible patient populations (BP > 145/85; HbA1c > 8.0) and whether or not relevant treatment was increased or added at relevant consultations. This is entailing a considerable amount of coding of frequency of dose data (usually entered as text rather than coded data) and coding of maximum doses of drugs to allow the identification of a population of patients who most closely match the behaviour. Although time consuming, this will provide a much more precise measure of a prescribing behaviour than we have been able to achieve in previous studies where we relied on routine data [5]. Data on the rates of performing behaviour three (examining feet) was available from 86 practices. For behaviours four and six, we found low rates of relevant computer codes both within and across practices. For behaviour four (providing advice on self management), we have computer code data for 68 practices (and from only 63 of these in the year following completion of the questionnaires); in addition, we have coded data on the provision of diabetes self-monitoring equipment (the use of which can form part of self-management) recorded from 47 practices. Patient education codes (behaviour six) were recorded in only 33 practices (and in 19 in the year following completion of the baseline questionnaires). Therefore, for these two behaviours we will be using the patients report data as our main measure of the behaviour.

### Quality and Outcomes Framework data

The QOF data are shown in Table 11. The QOF scores give a routinely available measure of clinical and organisational performance, though the rates of achievement against the organisational indicators are almost maximal, suggesting that these indicators will not usefully discriminate. QOF is also limited in terms of how the indicators relate to the clinical behaviours of interest within this project. Neither the behaviour 'giving advice about self-management' nor 'providing general education' have any useful match within the QOF data. For 'giving advice about weight management' the only indicator related to weight is 'patients' notes recording BMI' and, although this might reflect on the organisation of a practice, with mean achievement levels of 96% and a standard deviation of three, like the other organisational indicators, it too is unlikely to have sufficient variation to be discriminating. There is a good match for 'foot examination' and the mean achievement levels of 92% match the clinician and patient report well. For the other two behaviours 'prescribing additional anti-hypertensive drugs' and 'prescribing additional therapy for the management of glycaemic control,' there are indicators that could reflect the active performance of the two behaviours. In practices where clinicians are actively trying to tightly control both BP and glycaemic control, it would be reasonable to expect higher rates of patients with lower BP and HbA1c-and there is one QOF indicator for each of these with rates of performance of 80% and 68% respectively.

**Table 11:** QOF scores on each of the DM indicators, by practice (n = 99) for the 12 month period May 2008 to April 2009

QOF Indicator	% achievement
**Diabetes Mellitus**	**Mean (SD); min, max**

The percentage of patients with diabetes ... in the previous 15 months	

whose notes record BMI	96 (3); 82,100

who have a record of HbA1c or equivalent	98 (2); 85,100

in whom the last HbA1c is 7.5 or less (or equivalent)	68 (9); 54, 95

in whom the last HbA1c is 10 or less (or equivalent)	93 (4);76,100

who have a record of retinal screening	93 (4); 77, 100

with a record of the presence/absence of peripheral pulses	92 (6); 49, 100

with a record of neuropathy testing	92 (6); 49, 99

who have a record of their blood pressure	99 (1); 96, 100

in whom the last blood pressure is 145/85 or less*	80 (7); 59, 97

who have a record of micro-albuminuria testing	90 (6); 64, 100

who have a record of eGFR** or serum creatinine testing	98 (2); 85, 100

with a diagnosis of proteinuria or micro-albuminuria who are treated with ACE inhibitors (or A2 antagonists)*	93 (6); 75, 100

who have a record of total cholesterol	97 (2); 86, 100

whose last measured total cholesterol is 5mmol/l or less	84 (6); 66, 98

who have had influenza immunisation in the preceding 1 September to 31 March*	91 (6); 57, 100

The practice can produce a register of all patients aged 17 years and over with diabetes mellitus, which specifies whether the patient has Type 1 or Type 2 diabetes***	6 (0); 6,6

**Practice organisation**	

Total score for records and information	84.7 (5.4); 38.3, 87

Total score for information for patients	2.9 (0.4); 0.0, 3.0

Total score for education and training	27.2 (4.0); 0.0, 28

Total score for practice management	13.2 (1.9); 0.0, 13.5

Total score for medicines management	35.0 (5.3); 0.0, 36.0
	

**Overall QOF score**	973 (36); 730, 1000

* not dated to previous 15 months

** estimated glomerular filtration rate

*** numerator and denominator not available; QOF score reported

## Discussion

We have assembled an unparalleled data set from clinicians reporting on their cognitions in relation to the performance of six clinical behaviours involved in the management of people with one chronic disease (diabetes mellitus), using a range of organisational and individual level measures as well as information on the structure of the practice teams and across a large number of UK primary care practices.

In the context of generally falling response rates to postal questionnaire surveys of clinicians [49], we have previously had to deal with low response rates for theory-based questionnaires surveys [4-6,50]. As a consequence, we have had to contend with the fact that the data from such studies may not be representative. In this study, individual response rates varied by the clinical behaviour and whether it was the responsibility of the respondent to perform that behaviour (*e.g*., nurses who didn't prescribe didn't answer the two prescribing behaviours questions); nonetheless, we achieved individual response rates that varied within practices from 71 to 96%, figures far higher than usually achieved [49]. We assume that this is in part due to working with motivated practices (though this may compromise representativeness in a different way) and using a powerful behaviour change technique of offering reward (payment) based on satisfactory completion rates by practices rather than simply compensation for each individual's time involved in completing the questionnaires.

More importantly, because diabetes is a condition cared for by the integrated behaviours of multiple team members, we were particularly interested in achieving high levels of responses from all clinicians (physicians and nurses) within a practice. We achieved 100% response rates from clinicians in 40 practices, and achieved responses from over 80% of clinicians in 58 practices; for the questions about the six clinical behaviours, these figures rose to 60 and 76, respectively. However, despite working with research active practices, stressing the requirement for high response rates and recompensing them for their completion, for between 1 and 13 practices (depending on the section of the questionnaire) we received responses from less than 50% of eligible respondents.

Whilst the organisational measures were standard questionnaires (and achieved expected levels of internal consistency), our operationalisation of the individual cognition measures was good with measures of internal consistency all well within accepted ranges and good content coverage of the constructs. Many of the individual cognition scores are high, suggesting that respondents are already positively inclined towards performing the behaviours. These two groups of measures will together form a large part of our explanatory variables in explaining variation in rates of performing the behaviours. A standard analysis would calculate the variance in behaviour explained by each measure but, under circumstances such as these (where values are very positive), it is possible that contextual and environmental factors are important in whether or not the behaviours are successfully performed. Given the range of such factors that we have measured, we will be able to perform a more comprehensive analysis to generate hypotheses about where it might be best to intervene to improve performance.

We have successfully collected a number of different proxy measures of behaviour. These are a mix of individual level measures (self report, scenario simulation scores) and practice level measures (patient report, clinical data from practice computers, and QOF data). They also represent a range of measures of performing the behaviour (self-report) through to measures of the physiological consequences of the behaviour having been performed (measures from the practice computer such as BMI, BP, and HbA1c).

We extracted a considerable dataset relevant to the behaviours from the computers of the participating practices. Having defined six specific behaviours important to the management of patients with type 2 diabetes, it is salutary to reflect that only one (foot examination) was readily available within the computer records. For two of the behaviours (prescribing for BP control and glycaemic control), we will be able to compute an accurate measure (after considerable data processing), and for one other the computer record contained a physiological measure reflecting the performance of the behaviour across several links and interactions with other factors in a causal chain (BMI for advising about weight management). For the other two (advising behaviours), the computer record contained inconsistently recorded, and ultimately unusable, data.

These was no single, ideal, measure of behaviour, and any study such as this will have to balance the strengths and weaknesses of different measures of behaviour. It is not difficult to produce a list of potential biases-clinician self-report will be susceptible to a desirability reporting bias, simulated behaviour scores from the scenarios will be complicated to interpret and score, patient report will be susceptible to (at least) non-response, and recall biases and computer records will be susceptible to recording bias. However, for a study conducted on this scale, there is no ready alternative to the behaviour measures that we have collected, and whilst we will need to be sensitive to the potential shortcomings of the data in our analyses, we do not believe it is possible to produce better measures. While each of these measures on its own could present constraints as a true measure of the target behaviours, having all five measures will allow cross-validation.

Making simultaneous measurement across six behaviours allows a degree of comparison not previously reported in the implementation literature. It is clear from the data presented here that cognitions (all measured at the same point in time) vary across behaviours. Using direct estimation of intention as an example, this varies from 7.4 (out of a possible 10) for examining feet to 9.0 for providing weight management advice for 10 patients. The availability of such variation within and across behaviours should strengthen our ability to explain behaviour.

Given that the data held in practice computers represents the actions of different members of the practice team, the measures of self-report behaviour and simulated behaviour represent our only individual level measures of behaviour. In order to analyse the practice level data (from patient report, the practice computer systems, and QOF), we are going to have to deal with how best to aggregate our individual-level explanatory measures up to that of the team or organisation. Many previous measures have used the arithmetic mean, but it is by no means clear that this is the best metric for aggregation [51]. Approaches such as weighting systems using the scores of those whose role it is to perform the relevant behaviour may represent a better way forward.

The dataset that we have assembled represents one of the most comprehensive of its type, and the research team is very keen to maximise the use of it. To this end, we would welcome approaches to collaborate on the analysis of this data from other researchers and, once we have completed our main analyses, would be willing to explore making suitably anonymised data available to external groups for collaborative analyses.

## Conclusions

This paper is the first of a series of papers. It reports in detail the instrument development and data collected. Analyses of this large data set will, we hope, lead to the development of a series of strategies aimed at promoting the improvement of care for patients with diabetes as well as a series of rich insights into organisational and individual factors influencing clinician behaviour.

## Competing interests

Martin Eccles is Co-Editor in Chief of Implementation Science and Jeremy Grimshaw is a member of the Editorial Board of Implementation Science; all decisions on this paper were made by another editor.

## Authors' contributions

The study was conceived by MPE, JJF, MJ, NS, JMG, ME, GH, and MH. The study was run by SH and MPE with data handling and analyses by SH, ES, JP, and NS, and ongoing advice on operationalisation of theoretical constructs by ME, MJ, and JJF. Writing of the paper was led by MPE and SH with all authors commenting on drafts and approving the final version.

## Supplementary Material

Additional file 1 **Telephone interview schedule.pdf**. Pdf file. Organisational structure telephone interview schedule. Click here for file

Additional file 2 **Baseline Postal Questionnaire.pdf**. Pdf file. Baseline Postal Questionnaire incorporating organisational and clinical questionnaires and behaviour simulation measures. Click here for file

Additional file 3 **12 month clinician self report behaviour questionnaire.pdf**. Pdf file. 12 month clinician self report behaviour questionnaire. Click here for file

Additional file 4 **Computer Read Codes.pdf**. Pdf file. List of primary care practice computer data extraction items. Click here for file

Additional file 5 **Patient Questionnaire.pdf**. Pdf File. Patient questionnaire items. Click here for file

Additional file 6 **Deriving composite measures from the patients survey items.pdf**. Pdf file. Items and analysis for composite measures from the patient survey. Click here for file
