# Supplementary material for: Instrument development, data collection, and characteristics of practices, staff, and measures in the Improving Quality of Care in Diabetes (iQuaD) Study
Source: Implement Sci. 2011 Jun 9;6:61. doi: 10.1186/1748-5908-6-61 (PMC3130687; doi:10.1186/1748-5908-6-61)
Supplement: Additional file 1 — Telephone interview schedule.pdf. Pdf file. Organisational structure telephone interview schedule. [file 1748-5908-6-61-S1.PDF]

**Improving the delivery of care for patients with diabetes through understanding optimised team work and organisation in primary care.**

**Organisational Structure (Telephone interview schedule)**

**Practice demographics**

1. Is the location of your practice urban or rural — Rural Urban
2. Is it a dispensing practice? — Yes No
3. Is it a Training practice? — Yes No
4. What is the patient list size for your practice? ……………..
5. What is the % of patients on this list who are >65 years of age? ……………..
6. What proportions of the patients registered with your practice are:
    a) White British ……………..
    b) Black British ……………..
    c) British Asian ……………..
    d) British Chinese ……………..
    e) British Other ethnicity ……………..
    f) Non-British ……………..
    g) How often do you need to use interpreters? ……………..
7. Which postcodes does your practice cover? (or Carstairs Index score) ……………..
8. Which database system does your practice use?....................................................

**Structure**
***General primary care team members*** (Skill mix - ratio of doctors to non-medical clinical staff and of clinical to administrative staff)

9. **How many GPs** do you have in your practice? ………………

**a. How many are partners?**

| Name | How many sessions are covered by this Dr? |
|---|---|
| ………………………………………………………….. | ……………….. |
| ………………………………………………………….. | ……………….. |
| ………………………………………………………….. | ……………….. |
| ………………………………………………………….. | ……………….. |
| ………………………………………………………….. | ……………….. |
| ………………………………………………………….. | ……………….. |
| ………………………………………………………….. | ……………….. |
| ………………………………………………………….. | ……………….. |
| ………………………………………………………….. | ……………….. |
| ………………………………………………………….. | ……………….. |
| ………………………………………………………….. | ……………….. |

**b. How many are salaried?**

Name | How many sessions are covered by this Dr?
---|---
………………………………………………………….. | ………………..
………………………………………………………….. | ………………..
………………………………………………………….. | ………………..
………………………………………………………….. | ………………..
………………………………………………………….. | ………………..
………………………………………………………….. | ………………..
………………………………………………………….. | ………………..
………………………………………………………….. | ………………..
………………………………………………………….. | ………………..
………………………………………………………….. | ………………..
………………………………………………………….. | ………………..

**10. How many nurses** do you have in your practice?

**a. How many are:**

**Employed** by the practice?

Name | How many sessions are covered by this Nurse?
---|---
………………………………………………………….. | ………………..
………………………………………………………….. | ………………..
………………………………………………………….. | ………………..
………………………………………………………….. | ………………..
………………………………………………………….. | ………………..
………………………………………………………….. | ………………..
………………………………………………………….. | ………………..
………………………………………………………….. | ………………..
………………………………………………………….. | ………………..
………………………………………………………….. | ………………..
………………………………………………………….. | ………………..

b. **Attached** to the practice? (District nurses etc):

Name | How many sessions are covered by this Nurse?
---|---
………………………………………………………….. | ………………..
………………………………………………………….. | ………………..
………………………………………………………….. | ………………..
………………………………………………………….. | ………………..
………………………………………………………….. | ………………..
………………………………………………………….. | ………………..
………………………………………………………….. | ………………..
………………………………………………………….. | ………………..
………………………………………………………….. | ………………..
………………………………………………………….. | ………………..
………………………………………………………….. | ………………..

a.

11. **Are there any other health professionals** attached to (or employed by) your practice?

   a. If yes, note:
      i. Type of HP: Chiropodist, Psychologist, Healthcare assistant, Midwife etc
      ii. Number of sessions:
      iii. Nature of attachment – what they do, who employed by:

| Name | Role | No of sessions |
|---|---|---|
| …………………………………… | …………….. | ……………….. |
| …………………………………… | …………….. | ……………….. |
| …………………………………… | …………….. | ……………….. |
| …………………………………… | …………….. | ……………….. |
| …………………………………… | …………….. | ……………….. |
| …………………………………… | …………….. | ……………….. |
| …………………………………… | …………….. | ……………….. |
| …………………………………… | …………….. | ……………….. |
| …………………………………… | …………….. | ……………….. |
| …………………………………… | …………….. | ……………….. |
| …………………………………… | …………….. | ……………….. |

12. **How many administration staff** (managerial, clerical, reception) do you have in your practice?

| Name | Role | No of sessions |
|---|---|---|
| ……………………………………… | …………….. | ……………….. |
| ……………………………………… | …………….. | ……………….. |
| ……………………………………… | …………….. | ……………….. |
| ……………………………………… | …………….. | ……………….. |
| ……………………………………… | …………….. | ……………….. |
| ……………………………………… | …………….. | ……………….. |
| ……………………………………… | …………….. | ……………….. |
| ……………………………………… | …………….. | ……………….. |
| ……………………………………… | …………….. | ………………… |
| ……………………………………… | …………….. | ……………….. |
| ……………………………………… | …………….. | ……………….. |
| ……………………………………… | …………….. | ……………….. |
| ……………………………………… | …………….. | ……………….. |
| ……………………………………… | …………….. | ……………….. |
| ……………………………………… | …………….. | ……………….. |
| ……………………………………… | …………….. | ……………….. |
| ……………………………………… | …………….. | ……………….. |
| ……………………………………… | …………….. | ……………….. |
| ……………………………………… | …………….. | ……………….. |

13. How many staff members have left your practice in the past 12 months/ How many of these have been replaced?
    a. GPs (partners) ………left . ……….replaced
    b. GPs (salaried) ………left ……….replaced
    c. Nurses (employed) ………left ……….replaced
    d. Nurses (attached) ………left ……….replaced
    e. Administrative staff ………left ……….replaced

14. What is the rate of staff absence due to illness or sickness within your practice for:
    a. GPs? (per person) Days absent ……. How many episodes……
    b. Nurses (employed)? (per person) Days absent ……. How many episodes…….
    c. Administrative staff? (per person) Days absent ……. How many episodes…….

***15.*** **What additional specialist support services outside** of the practice are available to your practice in:

***a.*** **Community** *(e.g. community-based, attached diabetes nurses, "GPwSI" (GP with a special interest), DESMOND**,***

***b.*** **Secondary care** *(e.g. specialist care team)*

16. What is the booking interval for routine patient consultations at your practice (GP appointments)?

…..mins

17. How many surgery appointments do you offer in one week? ………………..

18. How many emergency appointments do you offer in one week? ………………..
(are these in addition to or included in above number – Q17?)

19. Does your practice use guidelines for chronic diseases? ………………..
    a. If yes: which guidelines? National local

***(ASK TO SEND US A COPY OF LOCAL GUIDELINES FOR DIABETES)***

20. Does your practice monitor prescribing? Y N

**(How is this done? Do they have an internal process or is monitoring done by PCT?)**

21. Does your practice provide feedback on performance to the practice team? Y N

**(How is this done? Do they have a formal process – e.g. annual appraisal?)**

22. What types of meetings does your practice hold? (e.g. “partnership meetings”, “practice management meetings”, “educational meetings”, “quality of care meetings”)

Note: type of meeting, how often held, who attends (e.g. GPs, Nurses and/or admin staff), how long they last.

| | | | |
|---|---|---|---|
| Practice | ……………… | …………….. | ……………….. |
| Partner | ……………… | …………….. | ……………….. |
| Educational | ……………… | …………….. | ……………….. |
| Admin | ……………… | …………….. | ……………….. |
| Clinical | ……………… | …………….. | ……………….. |
| ……………… | ……………… | …………….. | ……………….. |
| ……………… | ……………… | …………….. | ……………….. |
| ……………… | ……………… | …………….. | ……………….. |
| ……………… | ……………… | …………….. | ………………… |
| ……………… | ……………… | …………….. | ……………….. |
| ……………… | ……………… | …………….. | ……………….. |
| ……………… | ……………… | …………….. | ……………….. |
| ……………… | ……………… | …………….. | ……………….. |
| ……………… | ……………… | …………….. | ……………….. |
| ……………… | ……………… | …………….. | ……………….. |
| ……………… | ……………… | …………….. | ……………….. |

**23. Does your practice have a diabetes clinic?**

a. **If yes**: Does this sit within a more general structure of chronic disease management clinics?

   i. **If yes:** Describe the structure of care delivery within these management clinics both in general and if anything is different in relation to diabetes care:

   ii. Who runs the diabetes/chronic disease clinics? – what does this person do? Any admin/clerical support – if yes, who and what do they do?

   iii. Is there a GP available during the clinics? **Y** **N**

   **If yes:**

   Does a specific GP take a lead for diabetes (if so – which GP) or do patients see any or their own GP?

   Do patients see the GP on the day of their clinic/review visit – routinely or on a "if necessary" basis?

   iv. Patient recall intervals/appointment system – who does this? How often patients recalled? (e.g. 3m, 6m, 12m, other)

   v. What is the booking interval for annual review/diabetes review patients (how long is the appointment and who do they see – e.g. 30 min appointment might be 20 mins with nurse and 10 mins with Dr)?

   vi. Chasing up non-attenders - who does this? Procedure/how often?

vii. Referral policies/procedures – formal or informal.
What they do for newly diagnosed patients – e.g. longer 1st review etc, refer for education / dietician etc

What procedure do they have for management of patients on insulin? Do they initiate insulin at the practice or is this done following referral to Secondary Care?

viii. Provision of patient education:
What kind of education do they provide for patients?

Anything special for newly diagnosed patients?

ix. Do they use monitoring aids – e.g. patient diaries, blood or urine testing kits?

x. Anything else

……………………………………………………………………………………………………
……………………………………………………………………………………………………
……………………………………………………………………………………………………
……………………………………………………………………………………………………
……………………………………………………………………………………………………

24. Does your practice have a GP(s) or nurse(s) with a specialist interest in diabetes – has anyone had specialist training? What does the specialist do? Describe role in management of patients with diabetes – e.g. manage medication (prescribe), initiate insulin etc. What training have they had (formal and informal)? Is training on-going? Is training compulsory or sought out by individual health professional?

| Name | Role | Training |
|---|---|---|
| …………………………………… | …………….. | ……………….. |
| …………………………………… | …………….. | ……………….. |
| …………………………………… | …………….. | ……………….. |

| | | |
|---|---|---|
| …………………………………… | …………….. | ……………….. |
| …………………………………… | …………….. | ……………….. |
| …………………………………… | …………….. | ……………….. |
| …………………………………… | …………….. | ……………….. |

25. Any other information you think may be helpful to us:
