# Supplementary material for: Instrument development, data collection, and characteristics of practices, staff, and measures in the Improving Quality of Care in Diabetes (iQuaD) Study
Source: Implement Sci. 2011 Jun 9;6:61. doi: 10.1186/1748-5908-6-61 (PMC3130687; doi:10.1186/1748-5908-6-61)
Supplement: Additional file 2 — Baseline Postal Questionnaire.pdf. Pdf file. Baseline Postal Questionnaire incorporating organisational and clinical questionnaires and behaviour simulation measures. [file 1748-5908-6-61-S2.PDF]

Confidential

					/	1.4

Institute of Health&Society

Diabetes UK

Improving the delivery of care for patients with type 2 diabetes

Thank you very much for participating in this project.

This questionnaire has **THREE** sections.

SECTION 1: contains questions that should be answered by **all GPs and Nurses in your practice**. It should take you approximately **10 – 15 minutes** to complete this section.

SECTION 2: contains additional clinical management questions for **GPs and those Nurses** in your practice **who consider themselves to be involved in the delivery of care to patients with type 2 diabetes**

SECTION 3: contains a number of patient scenarios and should be also be completed by **GPs and those Nurses** in your practice **who consider themselves to be involved in the delivery of care to patients with type 2 diabetes.**

It should take you between **1 and 1.5 hours** to complete sections 2 and 3.

All responses to this questionnaire will be strictly confidential.

Newcastle University

Newcastle NHS Primary Care Trust

Background

Are you …? ☐ **Male** ☐ **Female**

In what year did you qualify? ☐☐☐☐

Are you a GP trainer in a vocational training scheme ☐ **Yes** ☐ **No**

How many sessions a week do you work? ☐☐ **Sessions**
(1 session = ½ day)

For the purposes of this study we are referring to all the staff working in your General Practice (both clinical and non-clinical) as being members of a "*Primary Care Team*".

What is your role within your General Practice? (Please circle)

Practice nurse   Nurse Specialist (Specialty:…………………)   Nurse Practitioner

Nurse Prescriber   District Nurse   GP (Salaried)   GP (Partner)

Other (please specify)………………………………

Within *YOUR* role, do you consider yourself to be involved in the routine delivery of care to patients with Type 2 diabetes in any way? (Please tick below)

☐ ***YES* -*** *If **YES**, What kind of care do you provide?* ....
....
....
....
....

- *Please now complete **<u>ALL THREE SECTIONS</u>** of this questionnaire.

☐ ***NO -*** please complete **<u>questions 1 to 10</u>** in **Section One** of this questionnaire (pages 3 to 6), then GO TO page 44.

If you have any queries about the study or this questionnaire, you can find our contact details on the back cover.

SECTION ONE: ***Questions about your primary care team***

> Please consider your answers to the following questions in relation to how your primary care team works ***in general.***

**Generally, in this practice …**	***Strongly disagree***						***Strongly agree***
We have a 'We are in it together' attitude	1	2	3	4	5	6	7
People keep each other informed about work-related issues in the primary care team	1	2	3	4	5	6	7
People feel understood and accepted by each other	1	2	3	4	5	6	7
There are real attempts to share information throughout the team	1	2	3	4	5	6	7
People in this team are always searching for fresh, new ways of looking at problems	1	2	3	4	5	6	7
We take the time needed to develop new ideas	1	2	3	4	5	6	7
People in the team co-operate in order to help develop and apply new ideas	1	2	3	4	5	6	7

**Generally, in this practice …**	***Not At All***						***Completely***
How far are you in agreement with your team's objectives?	1	2	3	4	5	6	7
To what extent do you think your team's objectives are clearly understood by other members of the team?	1	2	3	4	5	6	7
To what extent do you think your team's objectives can actually be achieved?	1	2	3	4	5	6	7
How worthwhile do you think these objectives are to the team?	1	2	3	4	5	6	7

**Generally, in this practice …**	***To a very little extent***						***To a very great extent***
Are team members prepared to question the basis of what the team is doing?	1	2	3	4	5	6	7
Does the team critically appraise potential weaknesses in what it is doing in order to achieve the best possible outcome?	1	2	3	4	5	6	7
Do members of the team build on each other's ideas in order to achieve the best possible outcome?	1	2	3	4	5	6	7

**Within your General Practice …**	***Strongly disagree***						***Strongly agree***
Procedures are designed to collect accurate information necessary for making decisions	1	2	3	4	5	6	7
Procedures are designed to provide opportunities to appeal or challenge the decision	1	2	3	4	5	6	7
Procedures are designed to have all sides affected by the decision represented.	1	2	3	4	5	6	7
Procedures are designed to generate standards so that decisions could be made with consistency.	1	2	3	4	5	6	7
Procedures are designed to hear the concerns of all those affected by the decision	1	2	3	4	5	6	7
Procedures provide useful feedback regarding the decision and its implementation.	1	2	3	4	5	6	7
Procedures are designed to allow for requests for clarification or additional information about the decision	1	2	3	4	5	6	7

**In this General Practice …**	***Strongly disagree***						***Strongly agree***
Primary care team members consider your viewpoint.	1	2	3	4	5	6	7
Primary care team members are able to suppress personal biases.	1	2	3	4	5	6	7
Primary care team members provide you with timely feedback about a decision and its implications.	1	2	3	4	5	6	7
Primary care team members treat you with kindness and consideration.	1	2	3	4	5	6	7
Primary care team members show concern for your rights as a clinician.	1	2	3	4	5	6	7
Primary care team members take steps to deal with you in a truthful manner.	1	2	3	4	5	6	7
Primary care team members consider your viewpoint.	1	2	3	4	5	6	7

**Within my primary care team, team members …**	***Strongly disagree***						***Strongly agree***
Help each other out if someone falls behind in his/her work.	1	2	3	4	5	6	7
Are willing to share their expertise with other members of the team	1	2	3	4	5	6	7
Try to act as peacemakers when other team members have disagreements	1	2	3	4	5	6	7
Take steps to try prevent problems with other team members	1	2	3	4	5	6	7
Are willing give their time to help team members who have work related problems	1	2	3	4	5	6	7
"Touch base" with other team members before initiating actions that may affect them	1	2	3	4	5	6	7
Encourage each other when someone is down	1	2	3	4	5	6	7
Provide constructive suggestions about how the team can improve in effectiveness	1	2	3	4	5	6	7
Are willing to risk disapproval to express their beliefs about what is best for the team	1	2	3	4	5	6	7
Attend and actively participate in team meetings	1	2	3	4	5	6	7
Always focus on what is wrong with our situation, rather than the positive side	1	2	3	4	5	6	7
Consume a lot of time complaining about trivial matters	1	2	3	4	5	6	7
Always find faults with what other team members are doing	1	2	3	4	5	6	7

Generally ...

	Strongly disagree						*Strongly agree*
My job allows me to make a lot of decisions on my own	1	2	3	4	5	6	7
I have a lot of say about what happens in my job	1	2	3	4	5	6	7
In my job, I have very little freedom to decide how I do my work	1	2	3	4	5	6	7
My job requires me to be creative	1	2	3	4	5	6	7
My job involves a lot of repetitive tasks	1	2	3	4	5	6	7
My job requires a high level of knowledge and skills	1	2	3	4	5	6	7
My job requires that I learn new things	1	2	3	4	5	6	7
I get to do a variety of different clinical tasks in my job	1	2	3	4	5	6	7
I have an opportunity to develop my own special abilities	1	2	3	4	5	6	7
My job requires working very fast	1	2	3	4	5	6	7
My job requires working very hard	1	2	3	4	5	6	7
I have to do an excessive amount of work	1	2	3	4	5	6	7
I have enough time to get the job done	1	2	3	4	5	6	7

Which primary care team members in your general practice are mainly involved in providing care for patients with diabetes?

**Name**	**Position** (GP, Nurse, receptionist etc)
--	--
--	--
--	--
--	--
--	--
--	--
--	--
--	--
--	--
--	--
--	--
--	--
--	--
--	--

The next questions are about you and are a little more personal. Please be re-assured that your responses to all questions are **completely confidential** and will not be disclosed to any other member of your primary care team.

**Have you recently …**	***Much less than usual***	***Same as usual***	***More than usual***	***Much more than usual***
Been able to concentrate on whatever you're doing?	1	2	3	4
Lost much sleep over worry?	1	2	3	4
Felt that you are playing a useful part in things?	1	2	3	4
Felt capable of making decisions about things?	1	2	3	4
Felt constantly under strain?	1	2	3	4
Felt you couldn't overcome your difficulties?	1	2	3	4
Been able to enjoy your normal day-to-day activities?	1	2	3	4
Been able to face up to your problems?	1	2	3	4
Been feeling unhappy and depressed?	1	2	3	4
Been losing confidence in yourself?	1	2	3	4
Been thinking of yourself as a worthless person?	1	2	3	4
Been feeling reasonably happy, all things considered?	1	2	3	4

Over the PAST 12months

How many ***episodes*** of sickness/illness have you had that resulted in you being absent from work? Number of episodes …………………

How many ***days in total*** were you absent from work due to sickness/illness? Total number of days absent ………..

Over the NEXT 12months:

Do you have any plans to leave your current position/move to new position? **Yes** **No**

If Yes – it would be helpful to us to know your reason for leaving

……

Thank you for completing the first 10 questions in SECTION 1.

If you **<u>DO NOT</u>** consider yourself to be **involved in the routine delivery of care** to patients with diabetes - **please now go to page 44** where you will find details of how to return this questionnaire to us, and space for you to make comments on the study.

If you **<u>DO</u>** consider yourself to be **involved in the routine delivery of care** to patients with diabetes, **please now CONTINUE to the next page (page 7),** and complete **the additional questions in Section 1.** You should then continue through the questionnaire to **complete SECTIONS 2 and 3.**

You will find that the following questions are the same or very similar to some of those we asked you earlier in Section 1.

This is because we would now like you to consider your answers to these questions ***in relation to how your primary care team works in the management of diabetes.***

**In relation to the management of diabetes …**	***Strongly disagree***						***Strongly agree***
We have a 'we are in it together' attitude	1	2	3	4	5	6	7
People keep each other informed about work-related issues in the primary care team	1	2	3	4	5	6	7
People feel understood and accepted by each other	1	2	3	4	5	6	7
There are real attempts to share information about the management of diabetes throughout the team	1	2	3	4	5	6	7
People in this team are always searching for fresh, new ways of looking at problems relating to the management of patients with diabetes	1	2	3	4	5	6	7
We take the time needed, in this team, to develop new ideas about how to manage patients with diabetes	1	2	3	4	5	6	7
People in the team co-operate in order to help develop and apply new ideas to the management of diabetes	1	2	3	4	5	6	7

**In relation to the management of diabetes …**	***Not At All***						***Completely***
How far are you in agreement with your team's objectives?	1	2	3	4	5	6	7
To what extent do you think your team's objectives are clearly understood by other members of the team?	1	2	3	4	5	6	7
To what extent do you think your team's objectives can actually be achieved?	1	2	3	4	5	6	7
How worthwhile do you think these objectives are to the team?	1	2	3	4	5	6	7

**In relation to the management of diabetes …**	***To a very little extent***						***To a very great extent***
Are team members prepared to question the basis of what the team is doing?	1	2	3	4	5	6	7
Does the team critically appraise potential weaknesses in what it is doing in order to achieve the best possible care for patients?	1	2	3	4	5	6	7
Do members of the team build on each other's ideas in order to achieve the best possible care?	1	2	3	4	5	6	7

In relation to the management of diabetes …

	Strongly disagree						*Strongly agree*
My job allows me to make a lot of decisions on my own	1	2	3	4	5	6	7
I have a lot of say about what happens in my job	1	2	3	4	5	6	7
In my job, I have very little freedom to decide how I do my work	1	2	3	4	5	6	7
My job requires me to be creative	1	2	3	4	5	6	7
My job involves a lot of repetitive tasks	1	2	3	4	5	6	7
My job requires a high level of knowledge and skills	1	2	3	4	5	6	7
My job requires that I learn new things	1	2	3	4	5	6	7
I get to do a variety of different clinical tasks in my job	1	2	3	4	5	6	7
I have an opportunity to develop my own special abilities	1	2	3	4	5	6	7
My job requires working very fast	1	2	3	4	5	6	7
My job requires working very hard	1	2	3	4	5	6	7
I have to do an excessive amount of work	1	2	3	4	5	6	7
I have enough time to get the job done	1	2	3	4	5	6	7

Thank you! You have now completed the first section of the questionnaire.

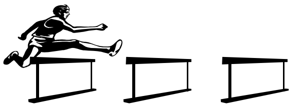

Please now continue to SECTION TWO on the next page.

<u>SECTION 2</u>

Questions about YOUR clinical management of patients with Type 2 diabetes

<u>PLEASE READ THE GUIDANCE AND NOTES BELOW VERY CAREFULLY BEFORE COMPLETING THIS SECTION OF THIS QUESTIONNAIRE</u>

**Section 2** contains questions asking about **YOUR clinical management** of six different aspects of care delivery for patients with **type 2 diabetes:**	
1. Giving advice about weight management	2. Prescribing additional antihypertensive drugs
3. Examining foot circulation & sensation	4. Providing advice about self-management
5. Prescribing additional therapy for the management of glycaemic control (HbA1c)	6. Providing general education about diabetes

Some of the six aspects of care covered in this questionnaire may not be a <u>regular</u> part of your role or routine clinical practice.

- However it is important to us to have your perspective on how you are involved in all these aspects of care delivery for patients with diabetes, ***so please complete all of the questions for all aspects of care that are part of your clinical role***.
- If you do not prescribe you can omit the two clinical care areas relating to prescribing (2 and 5 above), but we will ask you to tell us whose role this is in your practice.

There are a number of core questions that are repeated for each of the six clinical care areas. Also, within each clinical area some questions are worded very similarly but they are measuring different things.

- **It is important for the study that you answer them all.**

Questions are answered by circling one number.

<u>Please be sure to read the contents of the CLINICAL CARE AREA box at the beginning of each care area.</u>

Clinical care area 1:

GIVING ADVICE ABOUT WEIGHT MANAGEMENT

The questions in this clinical area ask about your **provision of advice about weight management**, both **in general** and **over the next 12 months,** to patients with type 2 diabetes whose **BMI is above a target** of

30 kg/m^2

even following previous management.

Is giving advice about weight management to patients with type 2 diabetes part of your clinical role?

If NO: please **write below whose role this is in your practice**, then **GO TO page 14**

Name (or **Initials** if you have already listed this person on page 5**)**

If YES: **please continue below and complete all of the following questions in this clinical area**:

Target BMI: 30 kg/m^2

In my management of patients with diabetes whose BMI is above target …

	Strongly disagree						***Strongly agree***
Overall, it is highly likely that they will be worse off if I *provide advice about weight management*	1	2	3	4	5	6	7
On balance, the consequences for me as a GP/Nurse (e.g. stress, time, future consultations etc.) will be worse in the long run if I *provide advice about weight management*	1	2	3	4	5	6	7
On balance, my life as a GP/Nurse will be easier in the long run if I *provide advice about weight management*	1	2	3	4	5	6	7

**I have a clear plan of …**	***Strongly disagree***						***Strongly agree***
How I will *provide advice about weight management*	1	2	3	4	5	6	7
Under what circumstances I will *provide advice about weight management*	1	2	3	4	5	6	7
When I will *provide advice about weight management*	1	2	3	4	5	6	7

Target BMI: 30 kg/m^2

Over the next 12 months, given 10 patients whose BMI is above target, for how many do you intend to *provide advice about weight management?*

0	1	2	3	4	5	6	7	8	9	10

In my management of patients with diabetes whose BMI is above target …

	Strongly disagree						***Strongly agree***
I think it is beneficial to them to *provide advice about weight management*	1	2	3	4	5	6	7
I think it is good practice to *provide advice about weight management*	1	2	3	4	5	6	7
I think it is a good use of my time to *provide advice about weight management*	1	2	3	4	5	6	7
I am expected to *provide advice about weight management*	1	2	3	4	5	6	7
I feel under pressure to *provide advice about weight management*	1	2	3	4	5	6	7
Most people whose opinions I value would approve if I *provide advice about weight management*	1	2	3	4	5	6	7
I am confident that I can *provide advice about weight management*	1	2	3	4	5	6	7
I intend to *provide advice about weight management*	1	2	3	4	5	6	7
It is entirely up to me whether or not I *provide advice about weight management*	1	2	3	4	5	6	7
I can overcome all obstacles, whatever they may be, to *provide advice about weight management*	1	2	3	4	5	6	7
I always consider *providing advice about weight management*	1	2	3	4	5	6	7
I plan to *provide advice about weight management*	1	2	3	4	5	6	7
It is my usual practice to *provide advice about weight management*	1	2	3	4	5	6	7
I expect to *provide advice about weight management*	1	2	3	4	5	6	7

I am confident that I can *provide advice about weight management* to any patient whose BMI is above target, even when …

	Strongly disagree						***Strongly agree***
The patient's BMI has been stable for 5 years	1	2	3	4	5	6	7
The patient's BMI has been increasing for 5 years	1	2	3	4	5	6	7
The patient is unwilling to discuss their weight	1	2	3	4	5	6	7
The clinic is busy and I am running 20 minutes late	1	2	3	4	5	6	7
The patient has osteoarthritis in their knees	1	2	3	4	5	6	7
The patient's BMI has been decreasing since you advised them to lose weight	1	2	3	4	5	6	7
The patient is depressed	1	2	3	4	5	6	7
The patient's values relating to body size differ from medical values	1	2	3	4	5	6	7
The patient has a low household income	1	2	3	4	5	6	7
Previous attempts by the patient to lose weight have been unsuccessful	1	2	3	4	5	6	7

Target BMI: 30 kg/m^2

Providing advice about weight management to patients whose BMI is above target *is something …*

	Strongly disagree						***Strongly agree***
I do frequently	1	2	3	4	5	6	7
I do automatically	1	2	3	4	5	6	7
I do without having to consciously remember	1	2	3	4	5	6	7
That makes me feel uncomfortable if I do not do it	1	2	3	4	5	6	7
I do without thinking	1	2	3	4	5	6	7
That would require effort not to do it	1	2	3	4	5	6	7
That belongs to my routine	1	2	3	4	5	6	7
I start doing before I realise I'm doing it	1	2	3	4	5	6	7
I would find hard not to do	1	2	3	4	5	6	7
I have no need to think about doing	1	2	3	4	5	6	7
That's typical for me	1	2	3	4	5	6	7
I have been doing for a long time	1	2	3	4	5	6	7

***Providing advice about weight management* to patients whose BMI is above target is something that for me routinely includes …** (please tick all that apply)

1. Advising about calorie restriction.. ☐
2. Advising about increasing exercise .... ☐
3. Prescribing exercise .... ☐
4. Providing a printed leaflet .... ☐
5. Referral to the practice nurse .... ☐
6. Referral to a dietician.. ☐
7. Suggesting a commercial weight loss organisation.. ☐
8. Suggesting a commercial gym/exercise organisation .... ☐
9. Other (please specify)...

....

If, during an appointment with a patient whose BMI is above target, you are really pressed for time and could only do one of the above (1 – 9) which ONE would you do? ....

**Target BMI**: 30 kg/m^2

I have made a clear plan regarding *providing advice about weight management* to patients whose BMI is above target if …

	Strongly disagree						***Strongly agree***
The patient's BMI has been stable for 5 years	1	2	3	4	5	6	7
The patient's BMI has been increasing for 5 years	1	2	3	4	5	6	7
The patient is unwilling to discuss their weight	1	2	3	4	5	6	7
The clinic is busy and I am running 20 minutes late	1	2	3	4	5	6	7
The patient has osteoarthritis in their knees	1	2	3	4	5	6	7
The patient's BMI has been decreasing since you advised them to lose weight	1	2	3	4	5	6	7
The patient is depressed	1	2	3	4	5	6	7
The patient's values relating to body size differ from medical values	1	2	3	4	5	6	7
The patient has a low household income	1	2	3	4	5	6	7
Previous attempts by the patient to lose weight have been unsuccessful	1	2	3	4	5	6	7

Over the past 12 months, for approximately how many of the last 10 patients whose BMI was above target did you *provide advice about weight management*?

0	1	2	3	4	5	6	7	8	9	10

………

Please now continue to Clinical care area 2 on next page

Clinical care area 2:

PRESCRIBING ADDITIONAL ANTIHYPERTENSIVE DRUGS

The questions in this clinical area ask about your **prescribing of additional antihypertensive drugs,** both **in general** and **over the next 12 months,** for patients with type 2 diabetes whose blood pressure (BP) is **5 mm Hg above a target** of

140 mm Hg Systolic BP or 80 mm Hg Diastolic BP

even following previous management.

Is deciding to prescribe antihypertensive drugs to patients with type 2 diabetes part of your clinical role?

If NO: please **write below whose role this is in your practice**, then **GO TO page 17**

Name (or **Initials** if you have already listed this person on page 5**)**

If YES: **please continue below and complete all of the following questions in this clinical area**:

Target BP: Systolic BP 140 mm Hg & Diastolic BP 80 mm Hg

In my management of patients with diabetes whose BP is 5 mm Hg above target …

	Strongly disagree						***Strongly agree***
Overall, it is highly likely that they will be worse off if I *prescribe them an additional antihypertensive drug*	1	2	3	4	5	6	7
On balance, the consequences for me as a GP/Nurse (e.g. stress, time, future consultations etc.) will be worse in the long run if I *prescribe an additional antihypertensive drug*	1	2	3	4	5	6	7
On balance, my life as a GP/Nurse will be easier in the long run if I *prescribe an additional antihypertensive drug*	1	2	3	4	5	6	7

**I have a clear plan of …**	***Strongly disagree***						***Strongly agree***
How I will manage them by *prescribing an additional antihypertensive drug*	1	2	3	4	5	6	7
Under what circumstances I will *prescribe them an additional antihypertensive drug*	1	2	3	4	5	6	7
When I will prescribe them *an additional antihypertensive drug*	1	2	3	4	5	6	7

Target BP: Systolic BP 140 mm Hg & Diastolic BP 80 mm Hg

Over the next 12 months, given 10 patients whose BP is 5 mm Hg above target, for how many do you intend to *prescribe an additional antihypertensive drug*?

0	1	2	3	4	5	6	7	8	9	10

In my management of patients with diabetes whose BP is 5 mm Hg above target …

	Strongly disagree						***Strongly agree***
I think it is beneficial to them to *prescribe an additional antihypertensive drug*	1	2	3	4	5	6	7
I think it is good practice to *prescribe an additional antihypertensive drug*	1	2	3	4	5	6	7
I think it is a good use of my time to *prescribe an additional antihypertensive drug*	1	2	3	4	5	6	7
I am expected to *prescribe an additional antihypertensive drug*	1	2	3	4	5	6	7
I feel under pressure to *prescribe an additional antihypertensive drug*	1	2	3	4	5	6	7
Most people whose opinions I value would approve if I *prescribe an additional antihypertensive drug*	1	2	3	4	5	6	7
I am confident that I can *prescribe an additional antihypertensive drug*	1	2	3	4	5	6	7
I intend to *prescribe an additional antihypertensive drug*	1	2	3	4	5	6	7
It is entirely up to me whether or not I *prescribe an additional antihypertensive drug*	1	2	3	4	5	6	7
I can overcome all obstacles, whatever they may be, to *prescribing an additional antihypertensive drug*	1	2	3	4	5	6	7
I always consider *prescribing an additional antihypertensive drug* for any patient	1	2	3	4	5	6	7
I plan to *prescribe an additional antihypertensive drug*	1	2	3	4	5	6	7
It is my usual practice to *prescribe an additional antihypertensive drug*	1	2	3	4	5	6	7
I expect to *prescribe an additional antihypertensive drug*	1	2	3	4	5	6	7

I am confident that I can *prescribe an additional antihypertensive drug* to any patient with diabetes whose BP is 5 mm Hg above target, even when …

	Strongly disagree						***Strongly agree***
The patient has COPD	1	2	3	4	5	6	7
The clinic is busy and I am running 20 minutes late	1	2	3	4	5	6	7
The patient is having mild side effects from their current antihypertensive medication	1	2	3	4	5	6	7
The patient is already on maximum dosages of three hypertensive drugs	1	2	3	4	5	6	7
The patient is elderly	1	2	3	4	5	6	7
The patient is on seven other drugs	1	2	3	4	5	6	7
The patient is unhappy with the idea of taking more drugs	1	2	3	4	5	6	7
The patient has a past history of falls	1	2	3	4	5	6	7
There are three minutes of the consultation remaining	1	2	3	4	5	6	7

Target BP: Systolic BP 140 mm Hg & Diastolic BP 80 mm Hg

Prescribing an additional antihypertensive drug **for any patient whose BP is 5 mm Hg above target is something …**

	Strongly disagree						***Strongly agree***
I do frequently	1	2	3	4	5	6	7
I do automatically	1	2	3	4	5	6	7
I do without having to consciously remember	1	2	3	4	5	6	7
That makes me feel uncomfortable if I do not do it	1	2	3	4	5	6	7
I do without thinking	1	2	3	4	5	6	7
That would require effort not to do it	1	2	3	4	5	6	7
That belongs to my routine	1	2	3	4	5	6	7
I start doing before I realise I'm doing it	1	2	3	4	5	6	7
I would find hard not to do	1	2	3	4	5	6	7
I have no need to think about doing	1	2	3	4	5	6	7
That's typical for me	1	2	3	4	5	6	7
I have been doing for a long time	1	2	3	4	5	6	7

I have made a clear plan regarding *prescribing an additional antihypertensive drug* for patients whose BP is 5 mm Hg above target if …

	Strongly disagree						***Strongly agree***
The patient has COPD	1	2	3	4	5	6	7
The clinic is busy and I am running 20 minutes late	1	2	3	4	5	6	7
The patient is having mild side effects from their current antihypertensive medication	1	2	3	4	5	6	7
The patient is already on maximum dosages of three hypertensive drugs	1	2	3	4	5	6	7
The patient is elderly	1	2	3	4	5	6	7
The patient is on seven other drugs	1	2	3	4	5	6	7
The patient is unhappy with the idea of taking more drugs	1	2	3	4	5	6	7
The patient has a past history of falls	1	2	3	4	5	6	7
There are three minutes of the consultation remaining	1	2	3	4	5	6	7

Over the past 12 months, for approximately how many of the last 10 patients whose BP was 5 mm Hg above target did you *prescribe an additional antihypertensive drug*?

0	1	2	3	4	5	6	7	8	9	10

……

Clinical care area 3:

EXAMINING FOOT CIRCULATION AND SENSATION

The questions in this clinical area ask both **in general** and **over the next 12 months,** about your **examination of the circulation and sensation in the feet** of patients with type 2 diabetes registered with your practice.

Is examining the circulation and sensation in the feet of patients with type 2 diabetes part of your clinical role?

If NO: please **write below whose role this is in your practice**, then **GO TO page 20**

Name (or **Initials)** if you have already listed this person on page 5**)**

If YES: **please continue below and complete all of the following questions in this clinical area**:

Target: Any patient with type 2 diabetes registered with your practice

**In my management of patients with diabetes …**	***Strongly disagree***						***Strongly agree***
Overall, it is highly likely that patients will be worse off if I *examine the circulation and sensation in their feet*	1	2	3	4	5	6	7
On balance, the consequences for me as a GP/Nurse (e.g. stress, time, future consultations etc.) will be worse in the long run if I *examine the circulation and sensation in their feet*	1	2	3	4	5	6	7
On balance, my life as a GP/Nurse will be easier in the long run if I *examine the circulation and sensation in their feet*	1	2	3	4	5	6	7

**I have a clear plan of …**	***Strongly disagree***						***Strongly agree***
How I will *examine the **circulation** in their feet*	1	2	3	4	5	6	7
How I will *examine the **sensation** in their feet*	1	2	3	4	5	6	7
Under what circumstances I will *examine the **circulation and sensation** in their feet*	1	2	3	4	5	6	7
When I will *examine the **circulation and sensation** in their feet*	1	2	3	4	5	6	7

Target: Any patient with type 2 diabetes registered with your practice

Over the next 12 months, given 10 patients with diabetes, for how many do you intend to *examine the circulation and sensation in their feet*?

0	1	2	3	4	5	6	7	8	9	10

**In my management of patients with diabetes …**	***Strongly disagree***						***Strongly agree***
I think it is beneficial to them to *examine the circulation and sensation in their feet*	1	2	3	4	5	6	7
I think it is good practice to *examine the circulation and sensation in their feet*	1	2	3	4	5	6	7
I think it is a good use of my time to *examine the circulation and sensation in their feet*	1	2	3	4	5	6	7
I am expected to *examine the circulation and sensation in their feet*	1	2	3	4	5	6	7
I feel under pressure to *examine the circulation and sensation in their feet*	1	2	3	4	5	6	7
Most people whose opinions I value would approve if I *examine the circulation and sensation in their feet*	1	2	3	4	5	6	7
I am confident that I can *examine the circulation and sensation in their feet*	1	2	3	4	5	6	7
I intend to *examine the circulation and sensation in their feet*	1	2	3	4	5	6	7
It is entirely up to me whether or not I *examine the circulation and sensation in their feet*	1	2	3	4	5	6	7
I can overcome all obstacles, whatever they may be, to *examine the circulation and sensation in their feet*	1	2	3	4	5	6	7
I always consider *examining the circulation and sensation in their feet*	1	2	3	4	5	6	7
I plan to *examine the circulation and sensation in their feet*	1	2	3	4	5	6	7
It is my usual practice to *examine the circulation and sensation in their feet*	1	2	3	4	5	6	7
I expect to *examine the circulation and sensation in their feet*	1	2	3	4	5	6	7

I am confident that I can *examine the …*

***CIRCULATION in the feet* of patients with diabetes, even when …**

	Strongly disagree						***Strongly agree***
The patient has poor hygiene	1	2	3	4	5	6	7
The clinic is busy and I am running 20 minutes late	1	2	3	4	5	6	7
The patient has poor mobility	1	2	3	4	5	6	7
The patient is wearing tights and needs additional time to undress	1	2	3	4	5	6	7

***SENSATION in the feet* of patients with diabetes, even when …**

	Strongly disagree						***Strongly agree***
The patient has poor hygiene	1	2	3	4	5	6	7
The clinic is busy and I am running 20 minutes late	1	2	3	4	5	6	7
The patient has poor mobility	1	2	3	4	5	6	7
The patient is wearing tights and needs additional time to undress	1	2	3	4	5	6	7

**Target**: Any patient with type 2 diabetes registered with your practice

I have made a clear plan regarding how to *examine the circulation and sensation in the feet* of patients with diabetes if …

	Strongly disagree						***Strongly agree***
The patient has poor hygiene	1	2	3	4	5	6	7
The clinic is busy and I am running 20 minutes late	1	2	3	4	5	6	7
The patient has poor mobility	1	2	3	4	5	6	7
The patient is wearing tights and needs additional time to undress	1	2	3	4	5	6	7

***Examining the circulation and sensation in the feet* of patients with diabetes is something …**

	Strongly disagree						***Strongly agree***
I do frequently	1	2	3	4	5	6	7
I do automatically	1	2	3	4	5	6	7
I do without having to consciously remember	1	2	3	4	5	6	7
That makes me feel uncomfortable if I do not do it	1	2	3	4	5	6	7
I do without thinking	1	2	3	4	5	6	7
That would require effort not to do it	1	2	3	4	5	6	7
That belongs to my routine	1	2	3	4	5	6	7
I start doing before I realise I'm doing it	1	2	3	4	5	6	7
I would find hard not to do	1	2	3	4	5	6	7
I have no need to think about doing	1	2	3	4	5	6	7
That's typical for me	1	2	3	4	5	6	7
I have been doing for a long time	1	2	3	4	5	6	7

As a routine part of YOUR CLINICAL ROLE, do you examine the ...

***CIRCULATION in the feet* of patients with diabetes? (Please tick)** ☐ **Yes** ☐ **No**

***SENSATION in the feet* of patients with diabetes?** (Please **tick)** ☐ **Yes** ☐ **No**

If, during an appointment with a patient with diabetes, you are really pressed for time and could only do one thing, which one of the following would you do in terms of examining their feet?
(please circle one)

Circulation Sensation Both Neither

Over the past 12 months, for approximately how many of the last 10 patients with diabetes did you *examine the circulation and sensation in their feet*?

0	1	2	3	4	5	6	7	8	9	10

....

Clinical care area 4:

PROVIDING ADVICE ABOUT SELF-MANAGEMENT

The questions in this clinical area ask, both **in general** and **over the next 12 months**, about your **provision of advice about self-management** to patients with type 2 diabetes registered with your practice.

Is providing advice about self-management to patients with type 2 diabetes part of your clinical role?

If NO: please **write below whose role this is in your practice**, then **GO TO page 24**

Name (or **Initials)** if you have already listed this person on page 5**)**

If YES: please continue below and complete all of the following questions in this clinical area:

Target: Any patient with type 2 diabetes registered with your practice

In my management of patients with diabetes …

	Strongly disagree						***Strongly agree***
Overall, it is highly likely that they will be worse off if I *provide advice about their self-management*	1	2	3	4	5	6	7
On balance, the consequences for me as a GP/Nurse (e.g. stress, time, future consultations etc.) will be worse in the long run if I *provide advice about their self-management*	1	2	3	4	5	6	7
On balance, my life as a GP/Nurse will be easier in the long run if I *provide advice about their self-management*	1	2	3	4	5	6	7

**I have a clear plan of …**	***Strongly disagree***						***Strongly agree***
How I will *provide advice about their self-management*	1	2	3	4	5	6	7
Under what circumstances I will *provide advice about their self-management*	1	2	3	4	5	6	7
When I will *provide advice about their self-management*	1	2	3	4	5	6	7

**Target**: Any patient with type 2 diabetes registered with your practice

Over the next 12 months, given 10 patients with diabetes, for how many of these patients do you intend to *provide advice about their self-management*?

0	1	2	3	4	5	6	7	8	9	10

**In my management of patients with diabetes …**	***Strongly disagree***						***Strongly agree***
I think it is beneficial to them to *provide advice about their self-management*	1	2	3	4	5	6	7
I think it is good practice to *provide advice about their self-management*	1	2	3	4	5	6	7
I think it is a good use of my time to *provide advice about their self-management*	1	2	3	4	5	6	7
I am expected to *provide advice about their self-management*	1	2	3	4	5	6	7
I feel under pressure to *provide advice about their self-management*	1	2	3	4	5	6	7
Most people whose opinions I value would approve if I *provide advice about their self-management*	1	2	3	4	5	6	7
I am confident that I can *provide advice about their self-management*	1	2	3	4	5	6	7
I intend to *provide advice about their self-management*	1	2	3	4	5	6	7
It is entirely up to me whether or not *I provide advice about their self-management*	1	2	3	4	5	6	7
I can overcome all obstacles, whatever they may be, to *provide advice about their self-management*	1	2	3	4	5	6	7
I always consider *providing advice about self-management*	1	2	3	4	5	6	7
I expect to *provide them with advice about self-management*	1	2	3	4	5	6	7
It is my usual practice to *provide advice about self-management* to patients with diabetes	1	2	3	4	5	6	7
I plan to *provide advice about their self-management*	1	2	3	4	5	6	7

I am confident that I can *provide advice about self-management*, even when …

	Strongly disagree						***Strongly agree***
The patient's diabetes is managed by diet alone	1	2	3	4	5	6	7
The clinic is busy and I am running 20 minutes late	1	2	3	4	5	6	7
The patient has their own monitor and expects you to prescribe specific lancets or strips	1	2	3	4	5	6	7
There are three minutes of the consultation remaining	1	2	3	4	5	6	7
The patient has a low household income	1	2	3	4	5	6	7
The patient has low educational attainment	1	2	3	4	5	6	7
The patient is worried about becoming hypoglycaemic and is diet controlled	1	2	3	4	5	6	7
The patient has been given conflicting advice about self-monitoring from other influential sources	1	2	3	4	5	6	7
The patient expects their doctor to manage their diabetes for them	1	2	3	4	5	6	7

Target: Any patient with type 2 diabetes registered with your practice

Providing patients with advice on the self-management **of their diabetes is something …**

	Strongly disagree						***Strongly agree***
I do frequently	1	2	3	4	5	6	7
I do automatically	1	2	3	4	5	6	7
I do without having to consciously remember	1	2	3	4	5	6	7
That makes me feel uncomfortable if I do not do it	1	2	3	4	5	6	7
I do without thinking	1	2	3	4	5	6	7
That would require effort not to do it	1	2	3	4	5	6	7
That belongs to my routine	1	2	3	4	5	6	7
I start doing before I realise I'm doing it	1	2	3	4	5	6	7
I would find hard not to do	1	2	3	4	5	6	7
I have no need to think about doing	1	2	3	4	5	6	7
That's typical for me	1	2	3	4	5	6	7
I have been doing for a long time	1	2	3	4	5	6	7

Providing patients with advice on the self-management **of their diabetes is something that for me routinely includes …** (please tick all that apply)

1. Advising about the nutritional content of their diet .... ☐
2. Providing disposable equipment for self-monitoring of blood glucose .... ☐
3. Suggesting NHS course for training diabetic patients in self-management........... ☐
4. Referral to the practice nurse .... ☐
5. Referral to a dietician.. ☐
6. Giving advice that takes into account individual circumstances............................ ☐
7. Other (please specify) ....

....

If, during an appointment with a patient with diabetes, you are really pressed for time and could only do one of the above (1 to 7), which ONE would you do? ……………………………….

Target: Any patient with type 2 diabetes registered with your practice

I have made a detailed plan regarding *providing advice about their self-management* to patients with diabetes if …

	Strongly disagree						*Strongly agree*
The patient's diabetes is managed by diet alone	1	2	3	4	5	6	7
The clinic is busy and I am running 20 minutes late	1	2	3	4	5	6	7
The patient has their own monitor and expects you to prescribe specific lancets or strips	1	2	3	4	5	6	7
There are three minutes of the consultation remaining	1	2	3	4	5	6	7
The patient has a low household income	1	2	3	4	5	6	7
The patient has low educational attainment	1	2	3	4	5	6	7
The patient is worried about becoming hypoglycaemic and is diet controlled	1	2	3	4	5	6	7
The patient has been given conflicting advice about self-monitoring from other influential sources	1	2	3	4	5	6	7
The patient expects their doctor to manage their diabetes for them	1	2	3	4	5	6	7

Over the past 12 months, for approximately how many of the last 10 patients with diabetes did you *provide advice about their self-management*?

0	1	2	3	4	5	6	7	8	9	10

....

Please now continue to Clinical care area 5 on next page

Clinical care area 5:

PRESCRIBING AN ADDITIONAL THERAPY FOR THE MANAGEMENT OF GLYCAEMIC CONTROL (HBA1C)

The questions in this clinical area ask about the **prescribing of an additional therapy**, both **in general** and **over the next 12 months**, for the management of HbA1c in patients with type 2 diabetes whose

HbA1c is higher than 8.0%

despite maximum dosage of two oral hypoglycaemic drugs.

Is deciding to prescribe additional therapy for patients with type 2 diabetes who are already on maximum dosage of two oral hypoglycaemic drugs part of your clinical role?

If NO: please **write below whose role this is in your practice**, then **GO TO page 27**

Name (or **Initials** if you have already listed this person on page 5**)**

If YES: please continue below and complete all of the following questions in this clinical area:

Target HbA1c: 8.0%

In my management of patients with diabetes whose HbA1c is above target …

	Strongly disagree						***Strongly agree***
Overall, it is highly likely that they will be worse off if I *prescribe an additional therapy*	1	2	3	4	5	6	7
On balance, the consequences for me as a GP/Nurse (e.g. stress, time, future consultations etc.) will be worse in the long run if I *prescribe an additional therapy*	1	2	3	4	5	6	7
On balance, my life as a GP/Nurse will be easier in the long run if I *prescribe an additional therapy*	1	2	3	4	5	6	7

I have a clear plan of …

	Strongly disagree						***Strongly agree***
How I will manage them by *prescribing an additional therapy*	1	2	3	4	5	6	7
Under what circumstances I will *prescribe an additional therapy*	1	2	3	4	5	6	7
When I will *prescribe an additional therapy*	1	2	3	4	5	6	7

Target HbA1c: 8.0%

Over the next 12 months, given 10 patients whose HbA1c is above target, for how many do you intend to *prescribe an additional therapy*?

0	1	2	3	4	5	6	7	8	9	10

In my management of patients with diabetes whose HbA1c is above target …

	Strongly disagree						***Strongly agree***
I think it is beneficial to them to *prescribe an additional therapy*	1	2	3	4	5	6	7
Good practice to *prescribe an additional therapy*	1	2	3	4	5	6	7
A good use of my time to *prescribe an additional therapy*	1	2	3	4	5	6	7
I am expected to *prescribe an additional therapy*	1	2	3	4	5	6	7
I feel under pressure to *prescribe an additional therapy*	1	2	3	4	5	6	7
Most people whose opinions I value would approve if I *prescribe an additional therapy*	1	2	3	4	5	6	7
I am confident that I can *prescribe an additional therapy*	1	2	3	4	5	6	7
I intend to *prescribe an additional therapy*	1	2	3	4	5	6	7
It is entirely up to me whether or not I *prescribe an additional therapy*	1	2	3	4	5	6	7
I can overcome all obstacles, whatever they may be, to *prescribing an additional therapy*	1	2	3	4	5	6	7
I always consider *prescribing an additional therapy*	1	2	3	4	5	6	7
I plan to *prescribe an additional therapy*	1	2	3	4	5	6	7
It is my usual practice to *prescribe an additional therapy*	1	2	3	4	5	6	7
I expect to *prescribe an additional therapy*	1	2	3	4	5	6	7

I am confident that I can *prescribe additional therapy* for a patient whose HbA1c above target, even when …

	Strongly disagree						***Strongly agree***
The patient has had laser treated maculopathy	1	2	3	4	5	6	7
The clinic is busy and I am running 20 minutes late	1	2	3	4	5	6	7
The patient is having mild side effects from their current hypoglycaemic medication	1	2	3	4	5	6	7
The patient is depressed	1	2	3	4	5	6	7
The patient has a BMI >35 Kg/m^2	1	2	3	4	5	6	7
The patient is on seven other drugs	1	2	3	4	5	6	7
The patient is unhappy with the idea of taking more drugs	1	2	3	4	5	6	7
There are three minutes of the consultation remaining	1	2	3	4	5	6	7

Target HbA1c: 8.0%

I have made a clear plan regarding the *prescribing of an additional therapy* for patients whose HbA1c is above target if …

	Strongly disagree						*Strongly agree*
The patient has had laser treated maculopathy	1	2	3	4	5	6	7
The clinic is busy and I am running 20 minutes late	1	2	3	4	5	6	7
The patient is having mild side effects from their current hypoglycaemic medication	1	2	3	4	5	6	7
The patient is depressed	1	2	3	4	5	6	7
The patient has a BMI >35 Kg/m^2	1	2	3	4	5	6	7
The patient is on seven other drugs	1	2	3	4	5	6	7
The patient is unhappy with the idea of taking more drugs	1	2	3	4	5	6	7
There are three minutes of the consultation remaining	1	2	3	4	5	6	7

***Prescribing an additional therapy* for any patient whose HbA1c is above target is something …**

	Strongly disagree						*Strongly agree*
I do frequently	1	2	3	4	5	6	7
I do automatically	1	2	3	4	5	6	7
I do without having to consciously remember	1	2	3	4	5	6	7
That makes me feel uncomfortable if I do not do it	1	2	3	4	5	6	7
I do without thinking	1	2	3	4	5	6	7
That would require effort not to do it	1	2	3	4	5	6	7
That belongs to my routine	1	2	3	4	5	6	7
I start doing before I realise I'm doing it	1	2	3	4	5	6	7
I would find hard not to do	1	2	3	4	5	6	7
I have no need to think about doing	1	2	3	4	5	6	7
That's typical for me	1	2	3	4	5	6	7
I have been doing for a long time	1	2	3	4	5	6	7

Over the past 12 months, for approximately how many of the last 10 patients whose HbA1c was above target did you *prescribe an additional therapy*?

0	1	2	3	4	5	6	7	8	9	10

………

Clinical care area 6:

PROVIDING GENERAL EDUCATION ABOUT DIABETES

The questions in this clinical area ask, both **in general** and **over the next 12 months**, about your **provision of general education** to patients with type 2 diabetes registered with your practice.

Is providing general education to patients with type 2 diabetes part of your clinical role?

If NO: please **write below whose role this is in your practice**, then **GO TO page 33**

Name (or **Initials** if you have already listed this person on page 5**)**

If YES: **please continue below and complete all of the following questions in this clinical area**:

Target: Any patient with type 2 diabetes registered with your practice

In my management of patients with diabetes …

	Strongly disagree						***Strongly agree***
Overall, it is highly likely that they will be worse off if I *provide general education about diabetes*	1	2	3	4	5	6	7
On balance, the consequences for me as a GP/Nurse (e.g. stress, time, future consultations etc.) will be worse in the long run if I *provide general education about diabetes*	1	2	3	4	5	6	7
On balance, my life as a GP/Nurse will be easier in the long run if I *provide general education about diabetes*	1	2	3	4	5	6	7

I have a clear plan of …

	Strongly disagree						***Strongly agree***
How I will *provide general education about diabetes*	1	2	3	4	5	6	7
Under what circumstances I will *provide general education about diabetes*	1	2	3	4	5	6	7
When I will *provide general education about diabetes*	1	2	3	4	5	6	7

Target: Any patient with type 2 diabetes registered with your practice

Over the next 12 months, given 10 patients with diabetes, for how many do you intend to *provide general education* about diabetes?

0	1	2	3	4	5	6	7	8	9	10

In my management of patients with diabetes …

	Strongly disagree						***Strongly agree***
I think it is beneficial to *provide general education about diabetes*	1	2	3	4	5	6	7
I think it is good practice to *provide general education about diabetes*	1	2	3	4	5	6	7
I think it is a good use of my time to *provide general education about diabetes*	1	2	3	4	5	6	7
I am expected to *provide general education about diabetes*	1	2	3	4	5	6	7
I feel under pressure to *provide general education about diabetes*	1	2	3	4	5	6	7
Most people whose opinions I value would approve if I *provide general education about diabetes*	1	2	3	4	5	6	7
I am confident that I can *provide general education about diabetes*	1	2	3	4	5	6	7
I intend to *provide general education about diabetes*	1	2	3	4	5	6	7
It is entirely up to me whether or not I *provide general education about diabetes*	1	2	3	4	5	6	7
I can overcome all obstacles, whatever they may be, *to providing general education about diabetes*	1	2	3	4	5	6	7
I always consider *providing general education about diabetes*	1	2	3	4	5	6	7
I plan to *provide general education about diabetes*	1	2	3	4	5	6	7
It is my usual practice to *provide general education about diabetes*	1	2	3	4	5	6	7
I expect to *provide general education about diabetes*	1	2	3	4	5	6	7

I am confident that I can *provide general education about diabetes*, even when …

	Strongly disagree						***Strongly agree***
The patient’s attendance on structured education programs is sporadic	1	2	3	4	5	6	7
The clinic is busy and I am running 20 minutes late	1	2	3	4	5	6	7
The patient is unenthusiastic about attending a structured education program	1	2	3	4	5	6	7
The patient has recently had an MI	1	2	3	4	5	6	7
The patient is depressed	1	2	3	4	5	6	7
There are three minutes of the consultation remaining	1	2	3	4	5	6	7
The patient has a low educational attainment	1	2	3	4	5	6	7
The patient is registered as partially sighted	1	2	3	4	5	6	7
The patient is hearing impaired	1	2	3	4	5	6	7
I have run out of printed leaflets	1	2	3	4	5	6	7
My Practice doesn’t support structured education	1	2	3	4	5	6	7

Target: Any patient with type 2 diabetes registered with your practice

Providing patients with general education **about diabetes is something …**

	Strongly disagree						*Strongly agree*
I do frequently	1	2	3	4	5	6	7
I do automatically	1	2	3	4	5	6	7
I do without having to consciously remember	1	2	3	4	5	6	7
That makes me feel uncomfortable if I do not do it	1	2	3	4	5	6	7
I do without thinking	1	2	3	4	5	6	7
That would require effort not to do it	1	2	3	4	5	6	7
That belongs to my routine	1	2	3	4	5	6	7
I start doing before I realise I'm doing it	1	2	3	4	5	6	7
I would find hard not to do	1	2	3	4	5	6	7
I have no need to think about doing	1	2	3	4	5	6	7
That's typical for me	1	2	3	4	5	6	7
I have been doing for a long time	1	2	3	4	5	6	7

Providing patients with general education **about diabetes is something that for me routinely includes …** *(please tick all that apply)*

1. Medical management .... ☐
2. What the symptoms of diabetes are... ☐
3. The time course of diabetes .... ☐
4. The cause of diabetes .... ☐
5. How the patient is involved in controlling diabetes .... ☐
6. Ensuring that they understand .... ☐
7. Providing a leaflet/printed materials .... ☐
8. Recommending a diabetes education course .... ☐
9. Recommending Diabetes UK .... ☐
10. Other (please specify) ....

....

If, during an appointment with a patient with diabetes, you are really pressed for time and could only do one of the above (1 to 10), which ONE would you do? ....

Target: Any patient with type 2 diabetes registered with your practice

I have made a clear plan regarding *providing general education* about diabetes if …

	Strongly disagree						***Strongly agree***
The patient's attendance on structured education programs is sporadic	1	2	3	4	5	6	7
The clinic is busy and I am running 20 minutes late	1	2	3	4	5	6	7
The patient is unenthusiastic about attending a structured education program	1	2	3	4	5	6	7
The patient has recently had an MI	1	2	3	4	5	6	7
The patient is depressed	1	2	3	4	5	6	7
There are three minutes of the consultation remaining	1	2	3	4	5	6	7
The patient has a low educational attainment	1	2	3	4	5	6	7
The patient is registered as partially sighted	1	2	3	4	5	6	7
The patient is hearing impaired	1	2	3	4	5	6	7
I have run out of printed leaflets	1	2	3	4	5	6	7
Your Practice doesn't support structured education	1	2	3	4	5	6	7

Over the past 12 months for approximately how many of the last 10 patients with diabetes, did you *provide general education*?

0	1	2	3	4	5	6	7	8	9	10

When managing patients with diabetes you have to perform multiple tasks under time constraints. We are interested in how you manage this process.

Due to clinic constraints I have to see a patient for annual review in a 20 minute appointment; I intend to prioritise each of these pairs of actions as follows … (please tick one box for each pair)

**Advise about weight management**	☐	☐	☐ **Give equal priority**	☐	☐	**Give education about diabetes**
**Advise about self-management**	☐	☐	☐ **Give equal priority**	☐	☐	**Advise about weight management**
**Give education about diabetes**	☐	☐	☐ **Give equal priority**	☐	☐	**Advise about self-management**

………

Thank you!

You have now completed the second section of the questionnaire.

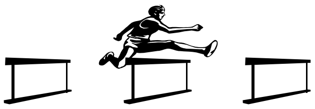

Please now continue to the final part of the questionnaire, SECTION THREE, on page 33.

SECTION THREE

The following pages contain a series of scenarios which include elements that may influence your management of patients with diabetes.

**We would like you to consider each scenario in the context of a routine review of a patient with type 2 diabetes.**

We appreciate that the observational and communication skills you may normally draw on during an actual consultation cannot be a factor in your decision.

Please try to consider each scenario based on the information presented then, in the space provided, record your decisions relating to:

- Management
- How difficult it was for you to decide your management of each scenario.

A worked example is provided on pages 34 & 35 overleaf.

WORKED EXAMPLE

No. 000 **Mrs Melody Dent, 2 Burnside Mews, Othertown** **Age 75 years**

Clinical Records

1. Consider the information presented in the clinical records for this patient

A Add **V** Values **H** Health | **X** All non-values **I** Immunisations **T** Templates | **C** Consultations **M** Medications **F** Forms & Admin | **P** Problems **N** Investigations **B** Allergies | **J** IOS Claims **L** Patient notes **Q** More

Active Problems: Type 2 DM 1992; Osteoarthritis; Hypertension

Smoker: Ex-smoker

Employment: Retired

Current medication: Co-codamol 2QDS PRN; Metformin 500mg TDS; Ramipril 10mg OD; Aspirin 75mg OD; Atenolol 100 mg OD; [illegible]e MR 40mg BD; Ipratropium 2 puffs QDS; Simvastatin 40 mg ON

2. Consider the clinical information presented in the table below relating to past reviews for this patient.

Significant past: Cancer of colon1998; COPD 1983; Fall, 2001, 2004, 2006

Allergies:

History: Reluctant to take additional drugs

	14/08/04	28/07/05	04/09/06	18/08/07	10/09/08	Would do	If time allows	What would you do?
**HbA1c**	8.3	8.0	7.3	7.7	8.4			
**Cholesterol**	5.4	3.9	4.2	4.1	4.3			
**Systolic BP**	[illegible]	[illegible]	[illegible]	[illegible]	145			
**Diastolic BP**	[illegible]	[illegible]	[illegible]	[illegible]	80			
**Albumin:Cre[illegible] ratio (norma[illegible]**	[illegible]	[illegible]	[illegible]	[illegible]	1.0			
**Foot inspect[illegible]**	[illegible]	[illegible]	[illegible]	[illegible]				
**BMI**	33	33	33	34	34			
**Patient education**	√							
**Weight management**	[illegible]	[illegible]	[illegible]	[illegible]				
**Self-managen[illegible]**	[illegible]	[illegible]	[illegible]	[illegible]				
**Notes**	[illegible]	[illegible] Start Ramipril	[illegible]	[illegible]ease [illegible]ipril				

3. Tick the aspects of care that you consider to be the most important for this patient and that you **would prioritise to do** during this consultation.

4. Tick the aspects of care that you also consider to be important for this patient and that you **would do <u>if time allows</u>** during this consultation.

- **This is a 15 minute consultation, so start with the most important elements of care and tick those that you *Would do* during this consultation and then tick the ones that you would prioritise to do only if you have time *(If time allows).***

- **Use the last three columns in the table above to indicate the elements of your management of this patient.**

- **Write a brief description of *what it is you would do* in the last column.**

You can add additional comments in the space provided below the table if necessary.

<u>Comments:</u>

5. Finally, please indicate below **how difficult** it was for you to make your decision about this patient scenario

On the scale 1 to 10, how difficult was it for you to make management decisions for this scenario?

**Not at all difficult**	0	1	2	3	4	5	6	7	8	9	10	**Extremely difficult**

If you wish to comment on this decision please do so here

No. 001 **Ms Sarah Mathers,** **Sycamore Avenue, Othertown** **Age 67 years**

Clinical Records

A Add
V Values
H Health
X All non-values
I Immunisations
T Templates
C Consultations
M Medications
F Forms & Admin
P Problems
N Investigations
B Allergies
J IOS Claims
L Patient notes
Q More

Active Problems: Type 2 DM 1996
Atrial Fibrillation
Hypertension

Smoker: Ex-smoker

Employment: Retired

Significant past: Hip replacement 2007
L CVA 2000
Depression, 1991, 2001

Current medication: Atenolol 100 mg OD; Metformin 800mg BD; Adizem-SR 120 BD; Simvastatin 40 mg ON; Doxazosin 8mg OD; Warfarin as directed

Allergies: Ramipril: rash 2006

History:

	14/08/05	28/07/06	04/09/07	18/03/08	10/09/08	Would do	Would do if time	What would you do?
**HbA1c**	8.3	8.4	7.8	7.6	7.6			
**Cholesterol**	4.7	5.2	4.6	4.6	4.8			
**Systolic BP**	162	150	140	148	156			
**Diastolic BP**	88	88	84	86	88			
**Albumin:Creatinine ratio (normal <2.5)**	0.8	1.5	1.4	0.9	1.3			
**Foot inspection**	√	√	√					
**BMI**	29	29.6	31.8	32.3	32.5			
**Patient education**	√		√					
**Weight management**	√	√	√	√				
**Self-management**		√						
**Notes**	Add Metformin	Add Ramipril Increase Metformin Add Doxazosin	Increase Doxazosin	Increase Doxazosin				

- **This is a 15 minute consultation, so start with the most important elements of care and tick those that you *Would do* during this consultation and then tick the ones that you would prioritise to do only if you have time *(Would do if time).***

- **Use the last three columns in the table above to indicate the elements of your management of this patient.**

- **Write a brief description of *what it is you would do* in the last column.**

You can add additional comments in the space provided below the table if necessary.

<u>Comments:</u>

On the scale 1 to 10, how difficult was it for you to make management decisions for this scenario?

**Not at all difficult**	0	1	2	3	4	5	6	7	8	9	10	**Extremely difficult**

If you wish to comment on this decision please do so here

No. 002 **Mrs Jane Tilbury, 2 Poplar Gardens, Othertown** **Age 75 years**

Clinical Records

**A** Add	**X** All non-values	**C** Consultations	**P** Problems	**J** IOS Claims
**V** Values	**I** Immunisations	**M** Medications	**N** Investigations	**L** Patient notes
**H** Health	**T** Templates	**F** Forms & Admin	**B** Allergies	**Q** More

Active Problems: Type 2 DM 1992
Osteoarthritis
Hypertension

Smoker: Ex-smoker

Employment: Retired

Significant past: Cancer of colon1998
COPD 1983
Fall, 2001, 2004, 2006

Current medication: Co-codamol 2QDS PRN; Metformin 500mg TDS; Ramipril 10mg OD; Aspirin 75mg OD; Atenolol 100 mg OD; Adipine MR 40mg BD; Ipratropium 2 puffs QDS; Simvastatin 40 mg ON

Allergies:

History: Reluctant to take additional drugs

(Please tick)

	14/08/04	28/07/05	04/09/06	18/08/07	10/09/08	would do	Would do if time	What would you do?
**HbA1c**	8.3	8.0	7.3	7.7	8.4			
**Cholesterol**	5.4	3.9	4.2	4.1	4.3			
**Systolic BP**	175	162	152	150	142			
**Diastolic BP**	95	92	92	88	80			
**Albumin:Creatinine ratio (normal <2.5)**	1.6	1.2	1.4	0.9	1.0			
**Foot inspection**	√	√		√				
**BMI**	33	33	33	34	34			
**Patient education**	√							
**Weight management**	√	√						
**Self-management**	√	√	√					
**Notes**	Add statin	Start Metformin Start Ramipril	Increase Ramipril	Increase Ramipril				

-
- **This is a 15 minute consultation, so start with the most important elements of care and tick those that you *Would do* during this consultation and then tick the ones that you would prioritise to do only if you have time *(Would do if time).***
- **Use the last three columns in the table above to indicate the elements of your management of this patient.**
- **Write a brief description of *what it is you would do* in the last column.**

You can add additional comments in the space provided below the table if necessary.

<u>Comments:</u>

On the scale 1 to 10, how difficult was it for you to make management decisions for this scenario?

**Not at all difficult**	0	1	2	3	4	5	6	7	8	9	10	**Extremely difficult**

If you wish to comment on this decision please do so here

No. 003 **Mr Jarrod Burns,** **58 St Thomas' Drive, Othertown** **Age 72 years**

Clinical Records

A Add
V Values
Health

X All non-values
I Immunisations
T Templates

C Consultations
M Medications
F Forms & Admin

P Problems
N Investigations
B Allergies

J IOS Claims
L Patient notes
Q More

Active Problems: Type 2 DM 1998
Angina
Hypertension
Heart failure

Smoker: Non-smoker

Employment: Retired

Significant past: Hip replacement 2005
Prostrate cancer 2006
CABG 1996

Current medication: Ramipril 10mg OD; Simvastatin 40 mg ON; Isosorbide mononitrate 20mg bd; Adipine MR 40mg BD

Allergies:

History:

	11/06/04	26/08/05	24/09/06	17/07/07	10/09/08	Would do	Would do if time	What would you do?
**HbA1c**	8.2	8.4	8.1	7.9	7.8			
**Cholesterol**	5.3	5.1	4.9	4.8	4.8			
**Systolic BP**	155	152	152	154	150			
**Diastolic BP**	78	74	74	74	78			
**Albumin:Creatinine ratio (normal <2.5)**	1.6	1.6	1.7	1.4	1.2			
**Foot inspection**	√	√	√	√				
**BMI**	34	34	33.6	33.3	33			
**Patient education**		√						
**Weight management**	√	√	√	√				
**Self-management**	√			√				
**Notes**	Encourage weight loss Add Adepine	Encourage weight loss Increase Adepine	Encourage weight loss	Encourage weight loss				

- **This is a 15 minute consultation, so start with the most important elements of care and tick those that you *Would do* during this consultation and then tick the ones that you would prioritise to do only if you have time *(Would do if time).***

- **Use the last three columns in the table above to indicate the elements of your management of this patient.**

- **Write a brief description of *what it is you would do* in the last column.**

You can add additional comments in the space provided below the table if necessary.

<u>Comments:</u>

On the scale 1 to 10, how difficult was it for you to make management decisions for this scenario?

**Not at all difficult**	0	1	2	3	4	5	6	7	8	9	10	**Extremely difficult**

If you wish to comment on this decision please do so here

No. 004 **Mr David Malloy,** **2 Dickens Cottages, Othertown** **Age 63 years**

Clinical Records

A Add
V Values
H Health

X All non-values
I Immunisations
T Templates

C Consultations
M Medications
F Forms & Admin

P Problems
N Investigations
B Allergies

J IOS Claims
L Patient notes
Q More

Active Problems: Type 2 DM 1999
Angina
Hypertension
Erectile dysfunction

Smoker: Smokes 15/day

Employment:

Significant past: TIA 2005
Hip replacement 2007

Current medication: Sildenafil 100mg 1pm; Adizem-SR 120mg bd; Isosorbide mononitrate 20mg bd; Simvastatin 40 mg ON; Metformin 500mg BD;

Allergies: Ramipril: cough
Atenolol: wheeze

History:

	14/08/05	28/07/06	04/09/07	18/03/08	10/09/08	would do	Would do if time	What would you do?
**HbA1c**	8.6	8.9	9.1	8.5	8.6			
**Cholesterol**	5.0	5.2	4.8	4.4	4.5			
**Systolic BP**	134	128	138	144	145			
**Diastolic BP**	82	84	85	84	85			
**Albumin:Creatinine ratio (normal <2.5)**	1.0	1.1	1.1	0.9	1.3			
**Foot inspection**	√	√	√					
**BMI**	38	38.4	38.6	38.6	38.6			
**Patient education**	√	√	√					
**Weight management**	√		√	√				
**Self-management**	√		√	√				
**Notes**			Add Metformin					

- **This is a 15 minute consultation, so start with the most important elements of care and tick those that you *Would do* during this consultation and then tick the ones that you would prioritise to do only if you have time *(Would do if time).***

- **Use the last three columns in the table above to indicate the elements of your management of this patient.**

- **Write a brief description of *what it is you would do* in the last column.**

You can add additional comments in the space provided below the table if necessary.

<u>Comments:</u>

On the scale 1 to 10, how difficult was it for you to make management decisions for this scenario?

**Not at all difficult**	0	1	2	3	4	5	6	7	8	9	10	**Extremely difficult**

If you wish to comment on this decision please do so here

Thank you!

You have now completed your questionnaire!

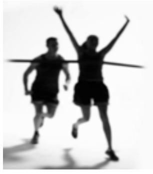

Is there any other comment you would like to make at this point?

____
____
____
____
____
____

Please now return your completed questionnaire, sealed in the envelope provided, to the study contact for your practice.

If you have any queries or wish to find out more about this study please contact:

Susan Hrisos

Research Associate
Institute of Health & Society
Newcastle University
21 Claremont Place
Newcastle upon Tyne
NE2 4AA

Tel: 0191 222 6774

Email: susan.hrisos@ncl.ac.uk
