# Supplementary material for: Instrument development, data collection, and characteristics of practices, staff, and measures in the Improving Quality of Care in Diabetes (iQuaD) Study
Source: Implement Sci. 2011 Jun 9;6:61. doi: 10.1186/1748-5908-6-61 (PMC3130687; doi:10.1186/1748-5908-6-61)
Supplement: Additional file 3 — 12 month clinician self report behaviour questionnaire.pdf. Pdf file. 12 month clinician self report behaviour questionnaire. [file 1748-5908-6-61-S3.PDF]

Confidential

Newcastle University

Diabetes UK

Institute of Health&Society

Improving the delivery of care for patients with type 2 diabetes

Clinician Follow-up Questionnaire 2009

1. What is your role within this General Practice? (Please circle)

Practice nurse   Nurse Specialist (Specialty:…………………)   Nurse Practitioner

Nurse Prescriber   District Nurse   GP (Salaried)   GP (Partner)

Other (please specify)………………………………

2. How long have you worked at this general practice?   ……. Years   ……. Months

Questions 3 & 4 ask about you **providing advice about weight management, both in general and over the past 12 months**, to patients with type 2 diabetes whose **BMI is above a target** of **30 kg/m^2** **even following previous management.**

3. Is giving advice about weight management to patients with type 2 diabetes part of your clinical role?   ☐ Yes **GO TO Q4**   ☐ No **GO TO Q5**

4. Over the past 12 months, given 10 patients with diabetes whose BMI was above target, **for how many did you *provide advice about weight management?***   ***(Please circle one number)***

0	1	2	3	4	5	6	7	8	9	10

Questions 5 & 6 ask about you **prescribing additional antihypertensive drugs, both in general and over the past 12 months,** for patients with type 2 diabetes whose blood pressure (BP) is **5mm Hg above a target of 140 mm Hg Systolic BP or 80 mm Hg Diastolic BP, even following previous management.**

5. Is deciding to prescribe antihypertensive drugs to patients with type 2 diabetes part of your clinical role?   ☐ Yes **GO TO Q6**   ☐ No **GO TO Q7**

6. Over the past 12 months, given 10 patients with diabetes whose BP was 5 mm Hg above target, **for how many did you *prescribe an additional antihypertensive drug?***

(Please circle one number)

0	1	2	3	4	5	6	7	8	9	10

Questions 7 & 8 ask about you **examining the circulation and sensation in the feet, both in general and over the past 12 months,** of patients with type 2 diabetes registered with your practice,.

7. Is examining the circulation and sensation in the feet of patients with type 2 diabetes part of your clinical role? ☐ **Yes GO TO Q8** ☐ **No GO TO Q9**

8. Over the past 12 months, given 10 patients with diabetes, **for how many did you *examine the circulation and sensation in their feet*?** ***(Please circle one number)***

0	1	2	3	4	5	6	7	8	9	10

Questions 9 & 10 ask about you **providing advice about self-management, both in general and over the past 12 months,** to patients with type 2 diabetes registered with your practice.

9. Is providing advice about self-management to patients with diabetes part of your clinical role?
☐ **Yes GO TO Q10** ☐ **No GO TO Q11**

10. Over the past 12 months, given 10 patients with diabetes, **for how many of these patients did you *provide advice about their self-management*?** ***(Please circle one number)***

0	1	2	3	4	5	6	7	8	9	10

Questions 11 & 12 ask, **both in general and over the past 12 months,** about you **prescribing an additional therapy** for the management of HbA1c in patients with type 2 diabetes whose **<u>HbA1c is higher than 8.0%</u> despite maximum dosage of two oral hypoglycaemic drugs.**

11. Is deciding to prescribe additional therapy for patients with type 2 diabetes part of your clinical role? ☐ **Yes GO TO Q12** ☐ **No GO TO Q13**

12. Over the past 12 months, given 10 patients with diabetes whose HbA1c is above target, for how many did you *prescribe an additional therapy*? ***(Please circle one number)***

0	1	2	3	4	5	6	7	8	9	10

Questions 13 & 14 ask, both **in general** and **over the past 12 months**, about you **providing general education** to patients with type 2 diabetes registered with your practice.

13. Is providing general education to patients with type 2 diabetes part of your clinical role?
☐ **Yes GO TO Q14** ☐ **No**

14. Over the past 12 months, given 10 patients with diabetes, for how many did you *provide general education* about diabetes? ***(Please circle one number)***

0	1	2	3	4	5	6	7	8	9	10

Thank you for completing this questionnaire.

Please now return it to us in the pre-paid envelope provided.
