# Supplementary material for: Instrument development, data collection, and characteristics of practices, staff, and measures in the Improving Quality of Care in Diabetes (iQuaD) Study
Source: Implement Sci. 2011 Jun 9;6:61. doi: 10.1186/1748-5908-6-61 (PMC3130687; doi:10.1186/1748-5908-6-61)
Supplement: Additional file 4 — Computer Read Codes.pdf. Pdf file. List of primary care practice computer data extraction items. [file 1748-5908-6-61-S4.PDF]

5 Byte Read Codes and Clauses for Diabetes UK Research Queries

C10%	Diabetes Mellitus (Excluding C10F8 Reaven's Syndrome)

Clause 2	
467A.	24 hour urine protein output
467E.	Urine protein level
467H.	Random urine protein level
46W%	Urine microalbumin
46N5.	24 hour urine protein excretion test
46N6.	24 hour urine albumin output
46N7.	Urine protein/creatinine index
46N8.	Urine microalbumin profile
46N3.	Urine total protein
46N4.	Urine albumin

Clause 3	
44J7.	Albumin / creatinine ratio
46TC.	Urine albumin:creatinine ratio
46TD.	Urine microalbumin:creatinine ratio

Clause 4	
1Z1%	Chronic renal impairment
R1103	[D]Microalbuminuria
C10EL	Type 1 diabetes mellitus with persistent microalbuminuria
C10FM	Type 2 diabetes mellitus with persistent microalbuminuria

Clause 5	
246%	O/E - blood pressure reading

Clause 6	**BNF Chapters**		**Read Codes**
2.2%	Diuretics	b2%	THIAZIDE DIURETICS
2.4%	Beta Androceptor Blocking Drugs	b3%	LOOP DIURETICS
2.5%	Hypertension and Heart Failure	b4%	POTASSIUM SPARING DIURETICS
2.6.2%	Calcium Channel Blockers	b5%	POTASSIUM SPARING COMPOUND DIURETICS
2.6.3%	Other Antanginal Drugs	b6%	OSMOTIC DIURETICS
		b7%	MERCURIAL DIURETICS
		b91%	DIURETICS+POTASSIUM SUPPLEMENT A-Z
		bd...	BETA-ADRENOCEPTOR BLOCKERS
		bk...	OTHER ANTIHYPERTENSIVES
		bf...	CENTRAL ANTIHYPERTENSIVES
		bg...	ADRENERGIC NEURONE BLOCKERS
		bh...	ALPHA-ADRENOCEPTOR BLOCKERS
		bA...	Ca Channel Block+Angiotensin-Converting Enzyme Inhib
		bl...	VASODILATORS USED IN ANGINA PECTORIS
		bB...	IF INHIBITOR

Clause 7	
42W%	Hb. A1C - diabetic control
42c%	HbA1 - diabetic control
44TB.	Haemoglobin A1c level
44TC.	Haemoglobin A1 level
44TL.	Total glycosylated haemoglobin level

Clause 8	**BNF Chapters**		**Read Codes**
6.1.1%	Insulins	f1...	SHORT-ACTING INSULIN PREPARATIONS
6.1.2%	Anti Diabetic Drugs	f2...	MEDIUM/LONG-ACTING INSULINS
		fw...	SHORT WITH INTERMEDIATE-ACTING INSULINS
		f3...	SULPHONYLUREAS
		f4...	BIGUANIDES
		ft...	OTHER DRUGS USED IN DIABETES

Clause 9	
44P..	Serum cholesterol
44P1.	Serum cholesterol normal
44P2.	Serum cholesterol borderline
44P3.	Serum cholesterol raised
44P4.	Serum cholesterol very high
44PH.	Total cholesterol measurement
44PJ.	Serum total cholesterol level
44OE.	Plasma total cholesterol level

Clause 10	
24E%	O/E - peripheral pulses R.-leg
24F%	O/E - peripheral pulses L.leg
585V.	Left dorsalis pedis doppler pressure
585W.	Right dorsalis pedis doppler pressure
585X.	Left posterior tibial doppler pressure

585Y.	Right posterior tibial doppler pressure
585a.	ABPI - Ankle brachial pressure index
585b.	Left dorsalis pedis ABPI
585c.	Right dorsalis pedis ABPI
585d.	Left posterior tibial ABPI
585e.	Right posterior tibial ABPI

Clause 11	
29B%	O/E - tactile sensation
29H%	O/E - vibration sense
66AE.	Feet examination
66AW.	Diabetic foot risk assessment
66Ab.	Diabetic foot examination
311A.	Monofilament foot sensation test
66Ac.	Diabetic peripheral neuropathy screening
29HA.	O/E - Vibration sense of right foot absent
29HB.	O/E - Vibration sense of left foot absent
29H4.	O/E - Vibration sense of right foot abnormal
29H5.	O/E - Vibration sense of right foot normal
29H6.	O/E - Vibration sense of left foot abnormal
29H7.	O/E - Vibration sense of left foot normal
29H8.	O/E - vibration sense left foot reduced
29H9.	O/E - vibration sense right foot reduced
2G5A.	O/E - Right diabetic foot at risk
2G5B.	O/E - Left diabetic foot at risk
2G5E.	O/E - Right diabetic foot at low risk
2G5F.	O/E - Right diabetic foot at moderate risk
2G5G.	O/E - Right diabetic foot at high risk
2G5H.	O/E - Right diabetic foot - ulcerated
2G5I.	O/E - Left diabetic foot at low risk
2G5J.	O/E - Left diabetic foot at moderate risk
2G5K.	O/E - Left diabetic foot at high risk
2G5L.	O/E - Left diabetic foot - ulcerated
9NND.	Under care of diabetic foot screener
8H7r.	Refer to diabetic foot screener

Clause 12	
22K%	Body Mass Index

Clause 13	
22A..	O/E - weight

Clause 14	
229..	O/E - height

Clause 15	
8H76.	Refer to dietician
8CA4.	Patient advised re diet
6799	Health ed. - diet
8CE4.	Diet leaflet given
66C%	Obesity monitoring

Clause 16	
8A17.	Self monitoring of blood glucose
8A18.	Self monitoring of urine glucose
8A19.	Self monitoring of blood and urine glucose
66AR.	Diabetes management plan given

Clause 17	**BNF Chapters**		**Read Codes**
6.1.6%	Diagnostic and Monitoring Agents for Diabetes Mellitus	pui..	BLOOD GLUCOSE METERS
		puk..	BLOOD GLUCOSE TESTING KIT
		puh..	BLOOD GLUCOSE TESTING STRIPS
		puj..	URINALYSIS MULTI-TESTING STRIPS

Clause 18	
9OLB.	Attended diabetes structured education programme
9OLE.	Attended DESMOND structured programme
9OLF.	Diabetes structured education programme completed
9OLG.	Attended XPERT diabetes structured education programme
9OLH.	Attended DAFNE diabetes structured education programme
9OLJ.	DAFNE diabetes structured education programme completed
9OLK.	DESMOND diabetes structured education programme completed
9OLL.	XPERT diabetes structured education programme completed
3881	Education score - diabetes
66Af.	Patient diabetes education review
8CE0.	Diabetic leaflet given
