# Supplementary material for: Instrument development, data collection, and characteristics of practices, staff, and measures in the Improving Quality of Care in Diabetes (iQuaD) Study
Source: Implement Sci. 2011 Jun 9;6:61. doi: 10.1186/1748-5908-6-61 (PMC3130687; doi:10.1186/1748-5908-6-61)
Supplement: Additional file 5 — Patient Questionnaire.pdf. Pdf File. Patient questionnaire items. [file 1748-5908-6-61-S5.PDF]

Confidential

Institute of Health&Society

Diabetes UK

Improving the care of patients with diabetes

This survey is about your experiences as a person with diabetes.

Who should complete this questionnaire?

The questions should be answered by the person named on the front of the envelope. If that person needs help to complete the questionnaire, the answers should be given from his/her point of view – not the point of view of the person who is helping.

How to complete this questionnaire

Some questions are answered by placing a tick inside a box √ others by circling a number inside of a box

1	2	3	4	5

Sometimes you will find a box you have ticked has an instruction to go to another question (e.g. **Go to Q6)**. By following these instructions carefully you will miss out questions that do not apply to you.

Don't worry if you make a mistake; simply cross out the mistake and put a tick or circle in the correct box.

Please do not write your name or address anywhere on the questionnaire.

When you have completed your questionnaire please return it to us in the pre-paid envelope provided.

Your participation in this survey is voluntary.

If you choose to take part, your answers will be treated **in confidence**.

Newcastle University

Newcastle NHS Primary Care Trust

About your diabetes

Q1. How old were you when you were first diagnosed with diabetes? (Your best estimate is fine)

I was ☐ years old

Q2. Do you have Type 1 or Type 2 diabetes? (please tick)

Type 1	
Type 2	
Don't know	

Q3. How do you control your diabetes now? (Please **tick** all that apply)

Insulin	
Tablets	
Diet	
Physical activity	
Other (please write in)	

Q4. Do you take medication for any other condition?

Yes ☐ ***Go to Q5*** **No** ☐ ***Go to Q6***

Q5. What type of medication(s) do you take? (Please **tick** all that apply)

Tablets for high blood pressure	
Tablets for high cholesterol	
Tablets for heart disease	
Other (please write in)	

Q6. Does your diabetes affect your day-to-day activities?

Yes ☐ **No** ☐

About your check-ups

Q7. Where do you go for your diabetes check-up, where your blood test results and treatment are reviewed? This check-up is sometimes known as an 'annual review' though some people have more or less than one every year.

(Please **tick** at least one box)

My doctor's surgery	
The hospital clinic	
Somewhere else (please write in)	
It varies	
Don't know	
I have never had a diabetes check-up ***Go to Q13 on page 6***	

Q8. In the last 12 months how many times have you had a diabetes check-up (where your blood test results and treatment are reviewed)?

(Please **tick** at least one box)

None ***Go to Q13 on page 6***	
Once	
Twice	
Three or more times	
Don't know	

About the care you have received over the last year

Q 9. Thinking about *the last 12 months*, when you received care for your diabetes from a doctor or nurse …

(please **circle** a number for each question)

	**Yes**	**No**	**Don't Know**
**a)** … were you given written information (e.g. a leaflet) about managing your weight?	1	2	3
**b)** … was it suggested to you to attend a weight loss organisation?	1	2	3
**c)** … was it suggested to you to attend a gym to help manage your diabetes?	1	2	3
**d)** … were you offered or did you receive "exercise on prescription" to help manage your diabetes?	1	2	3
**e)** … were you given advice about how to manage your weight?	1	2	3
**f)** … were you given advice about eating less to manage your weight?	1	2	3
**g)** … were you given advice about doing more exercise to manage your weight?	1	2	3
**h)** … were you asked to see a dietician to discuss managing your weight?	1	2	3
**i)** … were you asked to see a dietician to discuss managing your blood sugar?	1	2	3
**j)** … were you given advice about how YOU should manage YOUR diabetes?	1	2	3
**k)** … were you prescribed a drug to help you lose weight?	1	2	3
**l)** … were you provided with general information about diabetes?	1	2	3
**m)** Did this information help you to better understand diabetes?	1	2	3
**n)** **If the information did not help you,** can you tell us why?			

Q10. Again thinking about *the last 12 months*, when you received care for your diabetes from a doctor or nurse …

(please **circle** a number for each question)

	Rarely or not at all	***Some of the time***	***Almost always***
**a)**… did you discuss your ideas about the best way to manage your diabetes?	1	2	3
**b)** … were you given the chance to discuss different medications?	1	2	3
**c)** … did you discuss your goals in caring for your diabetes?	1	2	3
**d)** …were you given personal advice about the kinds of food to eat?	1	2	3
**e)** …were you given personal advice about your levels of physical activity?	1	2	3
**f)** … did you agree when your next appointment would be?	1	2	3
**g)** …did you agree a plan to manage your diabetes over the next 12 months?	1	2	3

About the management of your diabetes

Q11. In the last 12 months have you …

(please **tick** one box**)**

	**Yes**	**No**	**Don't know**
**a)** … had an eye test where a **photograph of the back of your eyes** was taken?			
**b)** … had your bare feet examined?			
**c)** … seen a dietician?			

Q12. In the past 12 months, did you get advice about any of the following with a GP or nurse in relation to your diabetes?

(Please **tick** all that apply)

**a)** The causes of diabetes	
**b)** The effects of being ill, e.g. having flu, on managing your diabetes	
**c)** Getting to and keeping to a certain weight	
**d)** What to expect if your blood glucose drops too low	
**e)** The reasons for taking prescribed medicines to manage your diabetes	
**f)** The long term health effects of your diabetes	
**g)** The impact of cholesterol levels on your diabetes	
**h)** The impact of blood pressure levels on your diabetes	
**i)** Getting your eyes checked	
**j)** Checking and looking after your feet	
**k)** How drinking alcohol can affect your diabetes	
**l)** The effects of stress on your diabetes	
**m)** The effects of tiredness on your diabetes	
**n)** What to do to manage your symptoms	
**o)** How did you get this advice:	
1. Verbally	
2. GP/nurse gave you a leaflet or other printed material	
3. The Diabetes UK website	
4. Other:	

How you manage your diabetes

Q13. How often do you test your own blood glucose levels? (Please **tick** one box only)

**a)** Never		***Go to Q15***
**b)** Less than once a day		***Go to Q14***
**c)** Once a day		***Go to Q14***
**d)** 2 or 3 times a day		***Go to Q14***
**e)** 4 or more times a day		***Go to Q14***

Q14. How do you use the results of your blood glucose tests? (please **tick** all that apply)

**a)** I check or alter the amount of insulin I take	
**b)** I check or alter my tablets	
**c)** To help me decide what I eat	
**d)** To help me decide how much physical activity to do	
**e)** To tell me if I am "hypo"	
**f)** To contact my diabetes doctor or nurse	
**g)** I write them down	
**h)** Other (Please write in)	

Q15. Overall, how would you rate your health during the past 4 weeks? (please **tick** one)

**a)** Excellent	
**b)** Very Good	
**c)** Good	
**d)** Fair	
**e)** Poor	

Q16. How confident are you that you understand …

*(please **circle** a number for each question)*

	Not at All Confident				Very Confident
**a)** … what to expect if your blood glucose drops too low?	1	2	3	4	5
**b)** … the reasons for taking prescribed medicines to manage your diabetes	1	2	3	4	5
**c)** … the long term health effects of your diabetes?	1	2	3	4	5
**d)** … the impact of cholesterol levels on your diabetes?	1	2	3	4	5
**e)** … the impact of blood pressure levels on your diabetes	1	2	3	4	5
**f)** … how drinking alcohol can affect your diabetes?	1	2	3	4	5
**g)** … the effects of stress on your diabetes?	1	2	3	4	5
**h)** … the effects of tiredness on your diabetes?	1	2	3	4	5

Q17. How confident are you that you can … *(please **circle** a number for each question)*

	Not at All Confident				Very Confident
**a)** … manage your diabetes?	1	2	3	4	5
**b)** … get to and keep to a certain weight?	1	2	3	4	5
**c)** … get your eyes checked?	1	2	3	4	5
**d)** … check and look after your feet?	1	2	3	4	5
**e)** … manage your exercise/activity levels?	1	2	3	4	5
**f)** … manage your diet ?	1	2	3	4	5
**g)** … take your medication as prescribed?	1	2	3	4	5

Q18. How good are you at … *(please **circle** a number for each question)*

	Not at All Good				Very Good
**a)** … eating the right foods to help you manage your diabetes?	1	2	3	4	5
**b)** … eating the right foods to help you manage your weight?	1	2	3	4	5
**c)** … being physically active to help you manage your diabetes?	1	2	3	4	5
**d)** … being physically active to help you manage your weight?	1	2	3	4	5

Education and knowledge

Q19. Have you ever … (please tick)

	**Yes**	**No**
**a)** … been offered the opportunity to attend an education or training course about how to help you manage your diabetes?		
**b)** … participated in an education or training course on how to help you manage your diabetes?		
**c)** … visited the Diabetes UK website?		

Q20. Do you know enough about … (please tick)

	**Yes**	**No**
**a)** … what you should eat to help you manage your diabetes?		
**b)** … what you should eat to help you manage your weight?		
**c)** … the role of physical activity in managing your diabetes?		
**d)** … the role of physical activity in managing your weight?		
**e)** … when to take your medication?		
**f)** … how much medication to take?		

Q21. Would you like to know more about …

(please tick)

	Yes	No
**a)** … what you should eat to help you manage your diabetes?		
**b)** … what you should eat to help you manage your weight?		
**c)** … how to get to and keep to a certain weight?		
**d)** … how to manage your diet?		
**e)** … the role of physical activity in managing your diabetes?		
**f)** … the role of physical activity in managing your weight?		
**g)** … how to manage your exercise/activity?		
**h)** … how to manage your symptoms?		
**i)** … when to take your medication?		
**j)** … how much medication to take?		
**k)** … how to manage your diabetes if you become ill?		
**l)** … checking your eyes?		
**m)** … checking and looking after your feet?		
**n)** … what to expect if your blood glucose drops too low?		
**o)** … the reasons for taking prescribed medicines to manage your diabetes		
**p)** … the long term health effects of your diabetes?		
**q)** … the impact of cholesterol levels on your diabetes?		
**r)** … the impact of blood pressure levels on your diabetes		
**s)** … how drinking alcohol can affect your diabetes?		
**t)** … the effects of stress on your diabetes?		
**u)** … the effects of tiredness on your diabetes?		

Your background

Q22. Are you … Male? ☐ Female? ☐ **What is your height?** ____________________

Q23. How old are you? ☐ years **What is your weight?** ____________________

Q24. How old were you when you left full-time education?

16 years or younger ☐
17 or 18 years ☐
19 years or older ☐
I have not had any formal education ☐

Q25. To which of these ethnic groups would you say you belong? (Please tick one box only)

a. WHITE

British ☐
Irish ☐

b. MIXED

White and Black Caribbean ☐
White and Black African ☐
White and Asian ☐

c. ASIAN OR ASIAN BRITISH

Indian ☐
Pakistani ☐
Bangladeshi ☐

d. BLACK OR BLACK BRITISH

Caribbean ☐
African ☐

e. CHINESE OR OTHER ETHNIC GROUP

Chinese ☐
Other ethnic group (Please write in) ☐

Thank you very much for your time

If you would like to make any further comments, please feel free to write them on the back of the questionnaire.

When you have completed the questionnaire please return it in the reply-paid envelope provided to:

Susan Hrisos
Institute of Health & Society
Newcastle University
21 Claremont Place
Newcastle upon Tyne
NE4 9UN
Tel: 0191 222 6774

Email:susan.hrisos@ncl.ac.uk

Practice ID: | | |

Additional comments:
