# Supplementary material for: Instrument development, data collection, and characteristics of practices, staff, and measures in the Improving Quality of Care in Diabetes (iQuaD) Study
Source: Implement Sci. 2011 Jun 9;6:61. doi: 10.1186/1748-5908-6-61 (PMC3130687; doi:10.1186/1748-5908-6-61)
Supplement: Additional file 6 — Deriving composite measures from the patients survey items.pdf. Pdf file. Items and analysis for composite measures from the patient survey. [file 1748-5908-6-61-S6.PDF]

Computing composite scores of patients' report of health professional behaviour
Summary, Methods and Results
Last updated 8 March 2011

Summary:
The patient questionnaire includes items which assess patients' report of whether they had been provided with weight advice, self-management advice, and general education and whether had their feet examined in the past 12 months. We aimed to use scores on these items as additional indicators of health professional behaviour. At previous team meetings, we identified the single item measures that most closely corresponded with the iQuaD behaviours and also additional items which assessed more specific aspects of each behaviour. Our aim was to produce a composite score for each behaviour based on the items that we identified. We ran the psychometrics prior to computing those scores, the details of which we present below. We verified the internal consistencies and ran principle components analyses on the items within each behaviour and then across behaviours.

Main results were as follows:

<u>Providing Weight management advice</u>
Cronbach's alpha : 0.80 (8 items)
Single item results: 51% yes
Composite score : mean=2.50 (SD=2.25). 70.6% Yes to at least 1 item

<u>Providing Self management advice</u>
Cronbach's alpha: 0.66 (3 items)
Single item results: 67.5% yes
Composite score: mean=1.51 (SD=0.99). 83.4% yes to at least 1 item

<u>Providing General Education</u>
Cronbach's alpha: 0.91 (18 items)
Single item results: 72.3% yes
Composite score: mean=7.44 (SD=5.16). 93% yes to at least 1 item

<u>Examining feet</u>
Single item results: 90.5% yes
(no composite score as only one item)

The principle components analysis (PCA) on items within each behaviour suggested that weight management, self-management and general education items involved more than one component. For behaviours with a low internal consistency, PCA results informed the decision of which items to remove (in particular for self-management). PCA on all items across behaviours showed that items loaded onto components represented by the placement of the items in the questionnaire rather than mapping onto the behaviours.

Given acceptable internal consistency levels and the clinical face validity of items composing each composite scale, we propose to use the composite scores for patients' report of receiving weight advice, self-management advice and general education for future analyses.

Detailed methods and results (SPSS screenshots) are presented in the pages that follow.

Method

We sought to use patients' report of whether they had been provided with weight advice, self-management advice, and general education and whether had their feet examined in the past 12 months as additional indicators of health professional behaviour. First, we identified single items that most closely represented each of these clinical behaviours (Table 1).

Table 1 – Single item measures of patients' report of health professionals' behaviour

Providing weight management advice

- Thinking about the last 12 months, when you received care for your diabetes from a doctor or nurse... were you given advice about how to manage your weight? (q9e). Response: Yes/No/Don't Know

Providing self-management advice

- Thinking about the last 12 months, when you received care for your diabetes from a doctor or nurse... were you given advice about how YOU should manage YOUR diabetes? (q9j). Response: Yes/No/Don't Know

Providing general education

- Thinking about the last 12 months, when you received care for your diabetes from a doctor or nurse... were you provided with general information about diabetes? (q9l). Response: Yes/No/Don't Know

Examining feet

- In the last 12 months have you … have you had your bare feet examined?" (q11b) (Yes/No/Don't know)

We also identified additional items that involved specific aspects of each of the behaviours using a consensus approach and clinical judgement (Table 2). For three of the four behaviours, multiple items were identified. We reduced these items to a single composite (mean) score for each of three behaviours following item analysis. No composite score was computed for foot exams as we only identified a single item representing patients' report of whether they had had their feet examined.

Table 2 – Candidate items composing each composite dependent variable

Providing weight management advice (8 items)

Q.9: Thinking about the last 12 months, when you received care for your diabetes from a doctor or nurse …(Yes/No/Don't know)

a) were you given written information (e.g. a leaflet) about managing your weight?
b) was it suggested to you to attend a weight loss organisation?
e) were you given advice about how to manage your weight?"
f) were you given advice about eating less to manage your weight?
g) were you given advice about doing more exercise to manage your weight?
h) were you asked to see a dietician to discuss managing your weight?
k) were you prescribed a drug to help you lose weight?

Q12 In the past 12 months did you get advice about .... (Yes/No)

c) getting to and keeping to a certain weight?

Providing self-management advice (6 items)

Q.9: Thinking about the last 12 months, when you received care for your diabetes from a doctor or nurse …(Yes/No/Don't know)

c) was it suggested to you to attend a gym to help manage your diabetes?
d) were you offered or did you receive "exercise on prescription" to help manage your diabetes?
i) were you asked to see a dietician to discuss managing your blood sugar?

j) were you given advice about how YOU should manage YOUR diabetes?

Q10 Again thinking about *the last 12 months*, when you received care for your diabetes from a doctor or nurse …… (rarely or not at all, some of the time, almost always)

a) did you discuss your ideas about the best way to manage your diabetes?

g) did you agree a plan to manage your diabetes over the next 12 months?

Providing general education (18 items)

Q9l. Thinking about the last 12 months, when you received care for your diabetes from a doctor or nurse... were you provided with general information about diabetes? (q9l). Response: Yes/No/Don't Know

Q10. Again thinking about *the last 12 months*, when you received care for your diabetes from a doctor or nurse …(Rarely – Always)

b)… were you given the chance to discuss different medications?
c) … did you discuss your goals in caring for your diabetes?
d) …were you given personal advice about the kinds of food to eat?
e) …were you given personal advice about your levels of physical activity?

Q12. In the past 12 months, did you get advice about any of the following with a GP or nurse in relation to your diabetes? (Tick all that apply)

a) The causes of diabetes
b) The effects of being ill, e.g. having flu, on managing your diabetes
d) What to expect if your blood glucose drops too low
e) The reasons for taking prescribed medicines to manage your diabetes
f) The long term health effects of your diabetes
g) The impact of cholesterol levels on your diabetes
h) The impact of blood pressure levels on your diabetes
i) Getting your eyes checked
j) Checking and looking after your feet
k) How drinking alcohol can affect your diabetes
l) The effects of stress on your diabetes
m) The effects of tiredness on your diabetes
n) What to do to manage your symptoms

Recoding

Items were recoded such that 1 represented a 'Yes' response, whereas 0 represented 'No'. Items from q10 were coded in two ways to compare psychometric qualities of each. First, items in q10 were dichotomised to more closely reflect dichotomous items from other items. Second, items in q10 were reverse-scored then recoded to 0 (rarely or not at all), 0.5 (some of the time), and 1 (almost always).

Internal consistency

Prior to computing the composite score for each behaviour, we assessed the internal consistency of the items for each of the three behaviours. We investigated whether removing items from the composite scale improved the internal consistency of the scale.

Component structure

We also investigated the component structure of each of the items within behaviours as well as all items across behaviours. We ran principle components analyses (PCA) with Verimax rotation on items representing each of three behaviours. We examined the scree plot and identified the number of components with an eigenvalue over 1. We then examined the rotated components matrix to investigate how each item loaded onto each component, using a .45 loading cut-off. For the individual PCAs, we included components with an eigenvalue over 1. For the overall PCA, we specified that three components should be extracted in order to verify whether they loaded onto each of the three multi-item behaviours (components).

Results

Internal consistency analysis

Providing weight advice

Case Processing Summary

		N	%
Cases	Valid	3127	87.1
	Excluded[a]	464	12.9
	Total	3591	100.0

a. Listwise deletion based on all variables in the procedure.

Reliability Statistics

Cronbach's Alpha	N of Items
.799	8

Item-Total Statistics

	Scale Mean if Item Deleted	Scale Variance if Item Deleted	Corrected Item-Total Correlation	Cronbach's Alpha if Item Deleted
q9a_0_1: in last 12 months - were you given written info about managing your weight	2.18	3.898	.528	.774
q9b_0_1: in last 12 months - was it suggested you attend a weight loss organisation	2.51	4.638	.354	.797
q9e_0_1: in last 12 months - were you given advice about how to manage your weight	2.10	3.585	.705	.742
q9f_0_1: in last 12 months - were you given advice about eating less to manage your	2.15	3.617	.691	.745
q9g_0_1: in last 12 months - were you given advice about doing more exercise to mana	2.16	3.775	.594	.763
q9h_0_1: in last 12 months - were you asked to see a dietician to discuss managing y	2.35	4.132	.483	.781
q9k_0_1: in last 12 months - were you prescribed a drug to help you lose weight	2.54	4.937	.168	.813
q12c_0_1: in past 12 months did you get advice about - getting to and keeping to a c	2.21	3.992	.482	.782

Providing self-management advice

Scoring option 1 for q10 (dichotomised)

Case Processing Summary

		N	%
Cases	Valid	2990	83.3
	Excluded[a]	601	16.7
	Total	3591	100.0

a. Listwise deletion based on all variables in the procedure.

Reliability Statistics

Cronbach's Alpha	N of Items
.593	6

Item-Total Statistics

	Scale Mean if Item Deleted	Scale Variance if Item Deleted	Corrected Item-Total Correlation	Cronbach's Alpha if Item Deleted
q9c_0_1: in last 12 months - was it suggested you attend a gym to help manage your	2.13	1.717	.243	.580
q9d_0_1: in last 12 months - were you offered or did you receive "exercise on prescr	2.17	1.802	.215	.589
q9j_0_1: in last 12 months - were you given advice about how you should manage your	1.57	1.331	.426	.501
q9i_0_1: in last 12 months - were you asked to see a dietician to discuss managing	2.04	1.581	.276	.569
q10a_dicho: in last 12 months - did you discuss your ideas about best way to manage yo	1.58	1.350	.402	.514
q10g_dicho: in last 12 months - did you agree a plan to manage your diabetes over next	1.71	1.304	.407	.512

Scoring option 2 for q10 (recode to 0, 0.5, 1)

Case Processing Summary

		N	%
Cases	Valid	2990	83.3
	Excluded[a]	601	16.7
	Total	3591	100.0

Case Processing Summary

		N	%
Cases	Valid	2990	83.3
	Excluded[a]	601	16.7
	Total	3591	100.0

a. Listwise deletion based on all variables in the procedure.

Reliability Statistics

Cronbach's Alpha	N of Items
.619	6

Item-Total Statistics

	Scale Mean if Item Deleted	Scale Variance if Item Deleted	Corrected Item-Total Correlation	Cronbach's Alpha if Item Deleted
q9c_0_1: in last 12 months - was it suggested you attend a gym to help manage your	1.815	1.524	.256	.608
q9d_0_1: in last 12 months - were you offered or did you receive "exercise on prescr	1.855	1.603	.234	.614
q9j_0_1: in last 12 months - were you given advice about how you should manage your	1.260	1.166	.426	.543
q9i_0_1: in last 12 months - were you asked to see a dietician to discuss managing	1.729	1.378	.305	.594
q10a_recode: in last 12 months - did you discuss your ideas about best way to manage yo	1.470	1.281	.456	.532
q10g_recode: in last 12 months - did you agree a plan to manage your diabetes over next	1.503	1.216	.434	.539

Providing general education

Case Processing Summary

		N	%
Cases	Valid	2959	82.4
	Excluded[a]	632	17.6
	Total	3591	100.0

a. Listwise deletion based on all variables in the procedure.

Reliability Statistics

Cronbach's Alpha	N of Items
.909	18

Item-Total Statistics

	Scale Mean if Item Deleted	Scale Variance if Item Deleted	Corrected Item-Total Correlation	Cronbach's Alpha if Item Deleted
q9l_0_1	7.2508	23.615	.457	.907
q10b_recode: in last 12 months - were you given the chance to discuss different medicat	7.6556	24.353	.348	.909
q10c_recode: in last 12 months - did you discuss your goals in caring for your diabetes	7.6141	23.775	.502	.906
q10d_recode: in last 12 months - were you given personal advice about kinds of food to	7.5333	23.595	.535	.905
q10e_recode: in last 12 months - were you given personal advice about your levels of ph	7.6130	23.858	.499	.906
q12a_0_1: in past 12 months did you get advice about - causes of diabetes	7.6367	23.008	.569	.904
q12b_0_1: in past 12 months did you get advice about - effects of being ill e.g. hav	7.6164	22.857	.594	.903
q12d_0_1: in past 12 months did you get advice about - what to expect if your blood	7.6110	22.896	.583	.903
q12e_0_1: in past 12 months did you get advice about - reasons for taking prescribed	7.4907	22.765	.586	.903
q12f_0_1: in past 12 months did you get advice about - long term health effects of y	7.5019	22.419	.664	.901
q12g_0_1: in past 12 months did you get advice about - impact of cholesterol levels	7.4856	22.359	.677	.900
q12h_0_1: in past 12 months did you get advice about - impact of blood pressure leve	7.5059	22.406	.668	.901
q12i_0_1: in past 12 months did you get advice about - getting your eyes checked	7.1730	23.739	.488	.906
q12j_0_1: in past 12 months did you get advice about - checking and looking after yo	7.2122	23.657	.473	.906
q12k_0_1: in past 12 months did you get advice about - how drinking alcohol can affe	7.5471	22.700	.608	.903
q12l_0_1: in past 12 months did you get advice about - effects of stress on your dia	7.7056	22.937	.632	.902
q12m_0_1: in past 12 months did you get advice about - effects of tiredness on your	7.7138	22.995	.624	.902
q12n_0_1: in past 12 months did you get advice about - what to do to manage your sym	7.6563	22.648	.665	.901

Results

Principle components analysis

Providing weight advice

Scree Plot

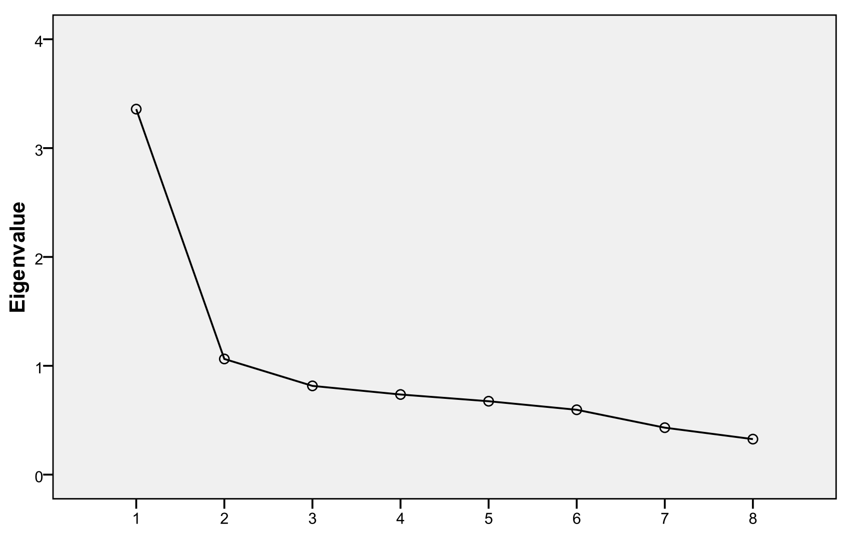

Total Variance Explained

Component	Initial Eigenvalues Total	% of Variance	Cumulative %	Rotation Sums of Squared Loadings Total	% of Variance	Cumulative %
1	3.358	41.976	41.976	3.092	38.656	38.656
2	1.063	13.285	55.261	1.328	16.605	55.261
3	.815	10.191	65.452			
4	.736	9.202	74.654			
5	.674	8.429	83.083			
6	.596	7.448	90.531			
7	.431	5.392	95.923			
8	.326	4.077	100.000			

Rotated Component Matrix[a]

	Component 1	Component 2
q9a: in last 12 months - were you given written info about managing your weight	.647	.168
q9b: in last 12 months - was it suggested you attend a weight loss organisation	.263	.650
q9e: in last 12 months - were you given advice about how to manage your weight	.829	.120
q9f: in last 12 months - were you given advice about eating less to manage your	.814	.136
q9g: in last 12 months - were you given advice about doing more exercise to mana	.747	.090
q9h: in last 12 months - were you asked to see a dietician to discuss managing y	.522	.377

q9k: in last 12 months - were you prescribed a drug to help you lose weight	-.047	.833
q12c: in past 12 months did you get advice about - getting to and keeping to a c	.649	.030

PCA - Providing self-management advice (continuous measure of q10)

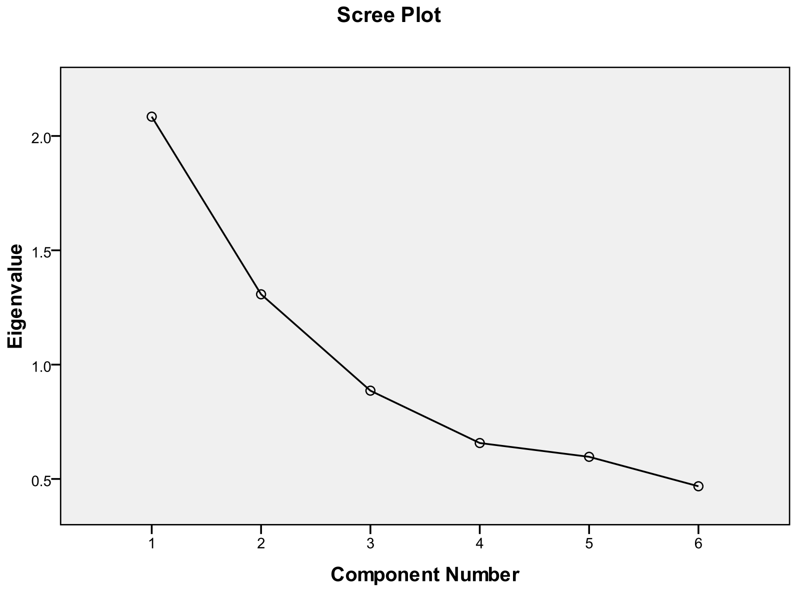

Total Variance Explained

Component	Initial Eigenvalues			Rotation Sums of Squared Loadings		
	Total	% of Variance		Total	% of Variance	
1	2.084	34.742	34.742	1.910	31.840	31.840
2	1.308	21.792	56.533	1.482	24.693	56.533
3	.886	14.768	71.301			
4	.657	10.952	82.253			
5	.597	9.947	92.200			
6	.468	7.800	100.000			

Rotated Component Matrix[a]

	Component	
	1	2
q9c: in last 12 months - was it suggested you attend a gym to help manage your	.069	.809
q9d: in last 12 months - were you offered or did you receive "exercise on prescr	.014	.816
q9i: in last 12 months - were you asked to see a dietician to discuss managing	.385	.368
q9j: in last 12 months - were you given advice about how you should manage your	.680	.160
q10a_reverse	.806	.012
q10g_reverse	.803	-.004

Extraction Method: Principal Component Analysis.

Rotation Method: Varimax with Kaiser Normalization.

Rotated Component Matrix[a]

	Component	
	1	2
q9c: in last 12 months - was it suggested you attend a gym to help manage your	.069	.809
q9d: in last 12 months - were you offered or did you receive "exercise on prescr	.014	.816
q9i: in last 12 months - were you asked to see a dietician to discuss managing	.385	.368
q9j: in last 12 months - were you given advice about how you should manage your	.680	.160
q10a_reverse	.806	.012
q10g_reverse	.803	-.004

Extraction Method: Principal Component Analysis.

Rotation Method: Varimax with Kaiser Normalization.

a. Rotation converged in 3 iterations.

Providing self-management advice: secondary internal consistency tests following PCA (using continuous measure of q10)

Reliability Statistics

Cronbach's Alpha	N of Items
.636	4

Item-Total Statistics

	Scale Mean if Item Deleted	Scale Variance if Item Deleted	Corrected Item-Total Correlation	Cronbach's Alpha if Item Deleted
q9i_0_1: in last 12 months - were you asked to see a dietician to discuss managing	1.553	.997	.268	.663
q9j_0_1: in last 12 months - were you given advice about how you should manage your	1.084	.784	.440	.551
q10a_recode: in last 12 months - did you discuss your ideas about best way to manage yo	1.296	.872	.495	.518
q10g_recode: in last 12 months - did you agree a plan to manage your diabetes over next	1.328	.808	.479	.520

Reliability Statistics

Cronbach's Alpha	N of Items
.662	3

Item-Total Statistics

	Scale Mean if Item Deleted	Scale Variance if Item Deleted	Corrected Item-Total Correlation	Cronbach's Alpha if Item Deleted
q9j_0_1: in last 12 months - were you given advice about how you should manage your	.887	.512	.389	.692
q10a_recode: in last 12 months - did you discuss your ideas about best way to manage yo	1.103	.543	.538	.497
q10g_recode: in last 12 months - did you agree a plan to manage your diabetes over next	1.135	.491	.513	.512

PCA - Providing general education

Total Variance Explained

Component	Initial Eigenvalues			Rotation Sums of Squared Loadings		
	Total	% of Variance	Cumulative %	Total	% of Variance	Cumulative %
1	7.107	39.485	39.485	4.966	27.586	27.586
2	1.603	8.905	48.390	2.609	14.493	42.079
3	1.340	7.442	55.832	2.475	13.752	55.832
4	.837	4.652	60.484			
5	.807	4.486	64.970			
6	.780	4.331	69.301			
7	.665	3.697	72.998			
8	.632	3.514	76.511			
9	.562	3.122	79.634			
10	.535	2.969	82.603			
11	.524	2.913	85.516			
12	.489	2.718	88.234			
13	.443	2.464	90.697			
14	.413	2.296	92.993			
15	.388	2.153	95.147			
16	.359	1.996	97.143			
17	.268	1.490	98.633			
18	.246	1.367	100.000			

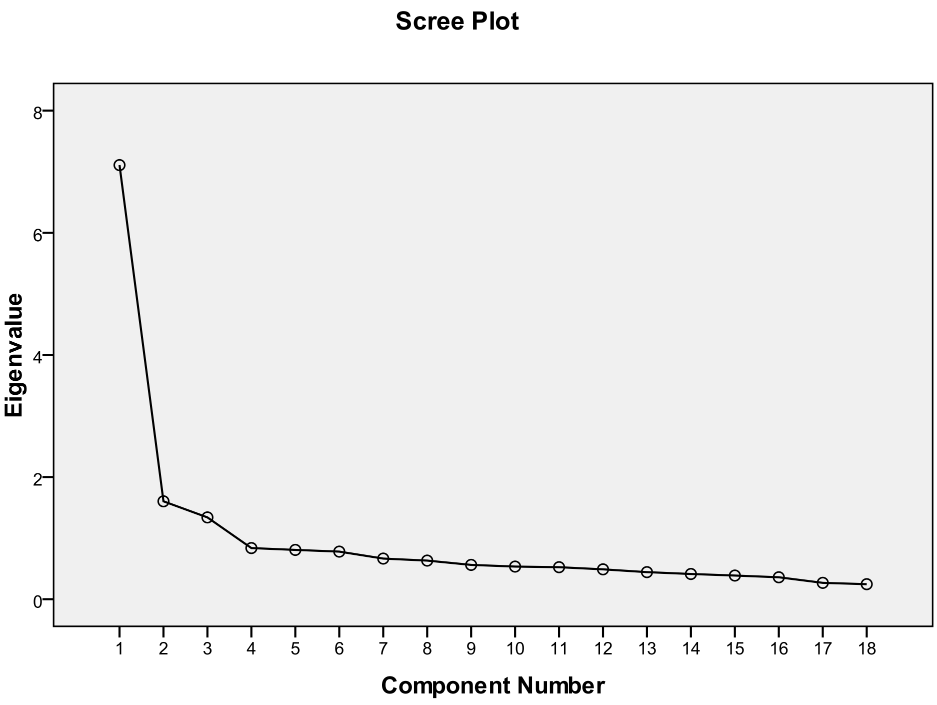
Scree Plot
Eigenvalue
8
6
4
2
0
1
2
3
4
5
6
7
8
9
10
11
12
13
14
15
16
17
18
Component Number

Rotated Component Matrix[a]

	Component		
	1	2	3
q9l_0_1	.222	.191	.576
q10b_recode: in last 12 months - were you given the chance to discuss different medicat	.154	.720	-.065
q10c_recode: in last 12 months - did you discuss your goals in caring for your diabetes	.226	.796	.085
q10d_recode: in last 12 months - were you given personal advice about kinds of food to	.196	.699	.309
q10e_recode: in last 12 months - were you given personal advice about your levels of ph	.195	.747	.186
q12a_0_1: in past 12 months did you get advice about - causes of diabetes	.541	.207	.255
q12b_0_1: in past 12 months did you get advice about - effects of being ill e.g. hav	.633	.206	.154
q12d_0_1: in past 12 months did you get advice about - what to expect if your blood	.634	.143	.185
q12e_0_1: in past 12 months did you get advice about - reasons for taking prescribed	.538	.212	.287
q12f_0_1: in past 12 months did you get advice about - long term health effects of y	.591	.236	.344
q12g_0_1: in past 12 months did you get advice about - impact of cholesterol levels	.608	.199	.379
q12h_0_1: in past 12 months did you get advice about - impact of blood pressure leve	.621	.200	.338
q12i_0_1: in past 12 months did you get advice about - getting your eyes checked	.197	.048	.821
q12j_0_1: in past 12 months did you get advice about - checking and looking after yo	.192	.064	.786
q12k_0_1: in past 12 months did you get advice about - how drinking alcohol can affe	.588	.131	.333
q12l_0_1: in past 12 months did you get advice about - effects of stress on your dia	.815	.092	.030
q12m_0_1: in past 12 months did you get advice about - effects of tiredness on your	.812	.088	.023
q12n_0_1: in past 12 months did you get advice about - what to do to manage your sym	.727	.220	.126

Extraction Method: Principal Component Analysis.

Rotation Method: Varimax with Kaiser Normalization.

a. Rotation converged in 4 iterations.

PCA - All items (<u>not</u> including single item foot exam) , except those removed following internal consistency tests – specified three components to be extracted

Total Variance Explained

Component	Initial Eigenvalues			Rotation Sums of Squared Loadings		
	Total	% of Variance	Cumulative %	Total	% of Variance	Cumulative %
1	9.661	33.314	33.314	6.247	21.542	21.542
2	2.150	7.413	40.727	3.893	13.424	34.967
3	2.020	6.965	47.691	3.690	12.725	47.691
4	1.354	4.670	52.362			
5	1.048	3.613	55.974			
6	.945	3.259	59.233			
7	.827	2.851	62.084			
8	.811	2.798	64.882			
9	.775	2.673	67.555			
10	.726	2.503	70.058			
11	.685	2.363	72.420			
12	.647	2.232	74.653			
13	.596	2.054	76.706			
14	.549	1.895	78.601			
15	.544	1.877	80.478			
16	.523	1.804	82.282			
17	.515	1.776	84.058			
18	.506	1.746	85.804			
19	.489	1.686	87.491			
20	.480	1.655	89.145			
21	.444	1.532	90.677			
22	.434	1.498	92.175			
23	.406	1.399	93.574			
24	.358	1.235	94.810			
25	.347	1.197	96.007			
26	.335	1.156	97.162			
27	.312	1.077	98.240			
28	.267	.920	99.160			
29	.244	.840	100.000			

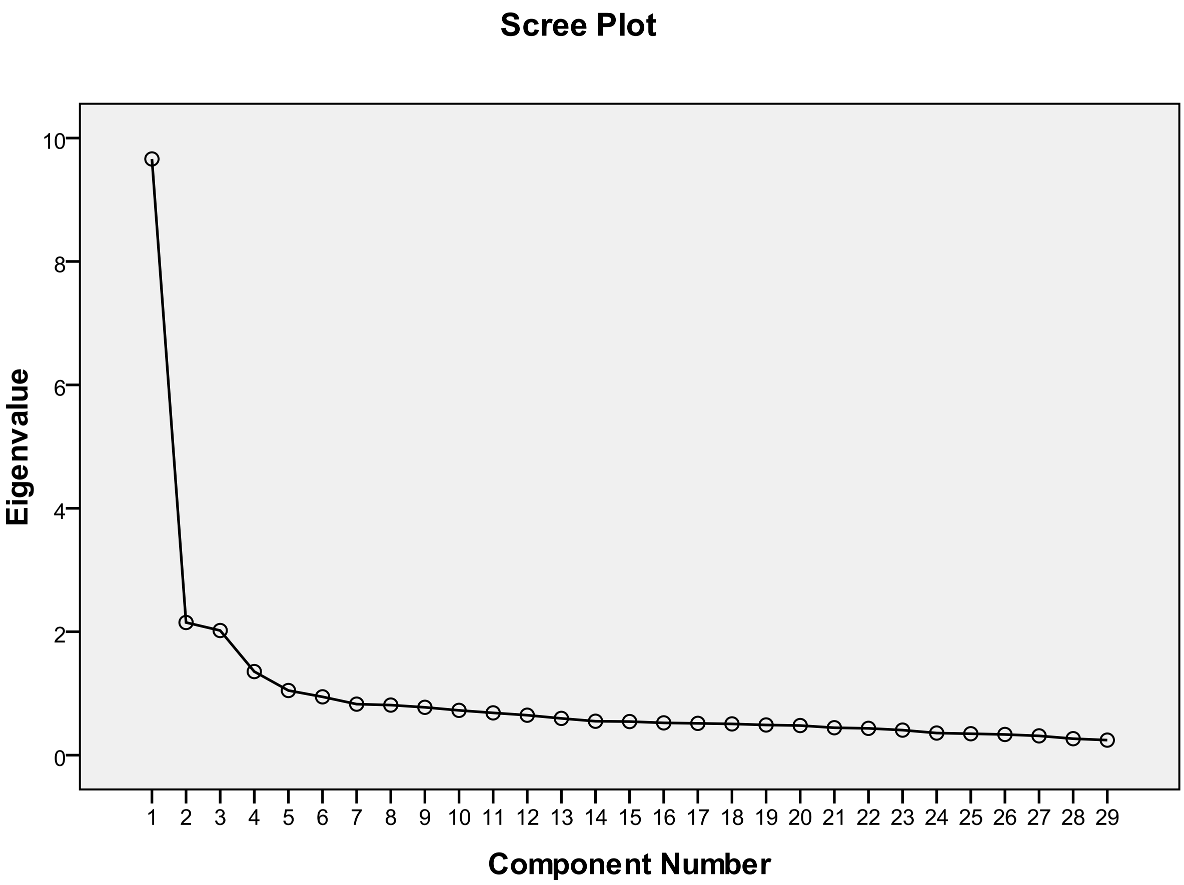

Rotated Component Matrix[a]

	Component		
	1	2	3
q9a_0_1: in last 12 months - were you given written info about managing your weight	.246	.627	.124
q9b_0_1: in last 12 months - was it suggested you attend a weight loss organisation	.042	.445	.051
q9e_0_1: in last 12 months - were you given advice about how to manage your weight	.209	.753	.215
q9f_0_1: in last 12 months - were you given advice about eating less to manage your	.194	.740	.164
q9g_0_1: in last 12 months - were you given advice about doing more exercise to mana	.161	.668	.183
q9h_0_1: in last 12 months - were you asked to see a dietician to discuss managing y	.107	.612	.049
q9j_0_1: in last 12 months - were you given advice about how you should manage your	.343	.466	.293
q9k_0_1: in last 12 months - were you prescribed a drug to help you lose weight	.018	.246	-.043
q9l_0_1	.354	.484	.138
q10a_recode: in last 12 months - did you discuss your ideas about best way to manage yo	.192	.130	.769
q10g_recode: in last 12 months - did you agree a plan to manage your diabetes over next	.244	.139	.703
q10b_recode: in last 12 months - were you given the chance to discuss different medicat	.142	-.065	.694
q10c_recode: in last 12 months - did you discuss your goals in caring for your diabetes	.237	.067	.809
q10d_recode: in last 12 months - were you given personal advice about kinds of food to	.249	.343	.619

q10e_recode: in last 12 months - were you given personal advice about your levels of ph	.207	.300	.664
q12a_0_1: in past 12 months did you get advice about - causes of diabetes	.555	.261	.185
q12b_0_1: in past 12 months did you get advice about - effects of being ill e.g. hav	.635	.115	.196
q12c_0_1: in past 12 months did you get advice about - getting to and keeping to a c	.474	.393	.248
q12d_0_1: in past 12 months did you get advice about - what to expect if your blood	.647	.072	.145
q12e_0_1: in past 12 months did you get advice about - reasons for taking prescribed	.600	.142	.194
q12f_0_1: in past 12 months did you get advice about - long term health effects of y	.649	.225	.211
q12g_0_1: in past 12 months did you get advice about - impact of cholesterol levels	.703	.139	.201
q12h_0_1: in past 12 months did you get advice about - impact of blood pressure leve	.701	.132	.193
q12i_0_1: in past 12 months did you get advice about - getting your eyes checked	.475	.310	.045
q12j_0_1: in past 12 months did you get advice about - checking and looking after yo	.460	.298	.069
q12k_0_1: in past 12 months did you get advice about - how drinking alcohol can affe	.648	.197	.121
q12l_0_1: in past 12 months did you get advice about - effects of stress on your dia	.738	.078	.111
q12m_0_1: in past 12 months did you get advice about - effects of tiredness on your	.736	.075	.102
q12n_0_1: in past 12 months did you get advice about - what to do to manage your sym	.694	.137	.227

Extraction Method: Principal Component Analysis.

Rotation Method: Varimax with Kaiser Normalization.

a. Rotation converged in 5 iterations.

Results – Patient report of clinicians' behaviour (descriptives)

Last updated 8 March 2011 by JP

Two sets of scores were produced for patients' report of clinicians' behaviour. First, the frequency of 'yes' responses to each single item measure was assessed. Second, we computed a composite score for each behaviour composed of the mean of the set of items with the highest internal consistency.

Providing weight management advice *Single item*

q9e_0_1: in last 12 months - were you given advice about how to manage your weight

		Frequency	Percent	Valid Percent	Cumulative Percent
Valid	no	1646	45.8	49.0	49.0
	yes	1716	47.8	51.0	100.0
	Total	3362	93.6	100.0	
Missing	System	229	6.4		
Total		3591	100.0		

Providing weight management advice *Complex DV (8 items)*

Weight_advice_PATIENT_DV - 8 item patient report of receiving weight management advice

		Frequency	Percent	Valid Percent	Cumulative Percent
Valid	.00	1057	29.4	29.4	29.4
	1.00	508	14.1	14.1	43.6
	2.00	343	9.6	9.6	53.1
	3.00	397	11.1	11.1	64.2
	4.00	449	12.5	12.5	76.7
	5.00	404	11.3	11.3	87.9
	6.00	290	8.1	8.1	96.0
	7.00	119	3.3	3.3	99.3
	8.00	24	.7	.7	100.0
	Total	3591	100.0	100.0	

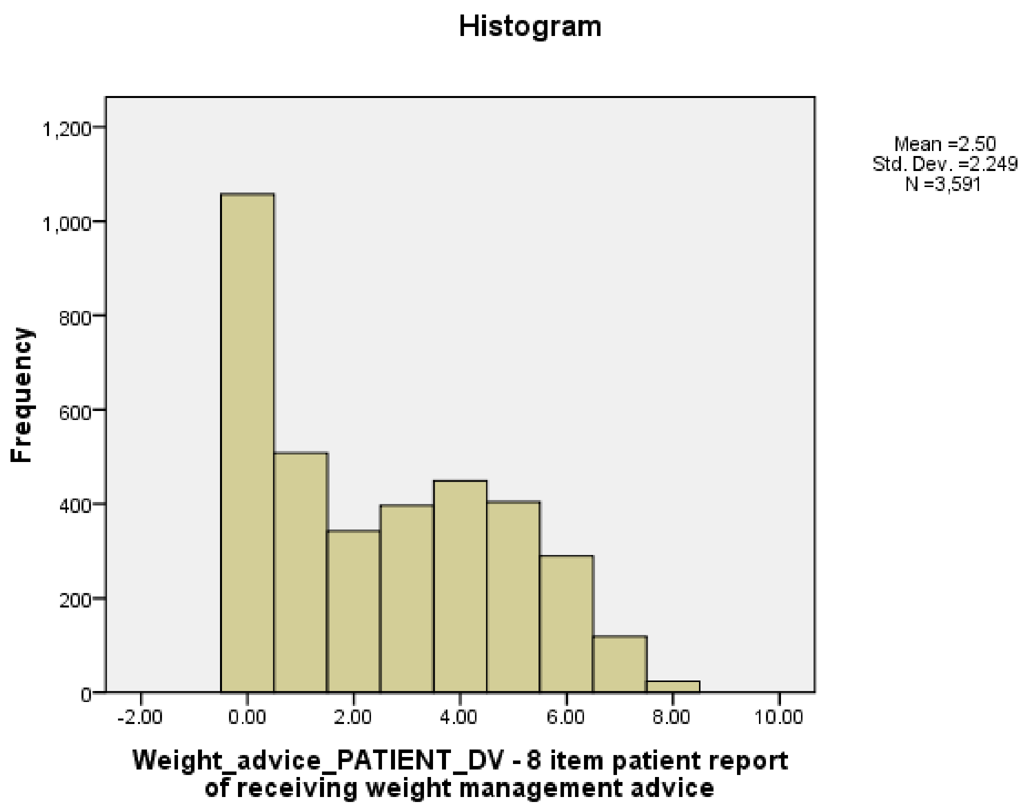

Providing self-management advice *Single item*

q9j_0_1: in last 12 months - were you given advice about how you should manage your

		Frequency	Percent	Valid Percent	Cumulative Percent
Valid	no	1103	30.7	32.5	32.5
	yes	2292	63.8	67.5	100.0
	Total	3395	94.5	100.0	
Missing	System	196	5.5		
Total		3591	100.0		

Providing self-management advice *Complex DV (3 items)*

SelfMan_PATIENT_DV - 3 item patient report of receiving self-management advice

		Frequency	Percent	Valid Percent	Cumulative Percent
Valid	.00	572	15.9	16.6	16.6
	.50	242	6.7	7.0	23.6
	1.00	638	17.8	18.5	42.1
	1.50	518	14.4	15.0	57.2
	2.00	579	16.1	16.8	74.0
	2.50	385	10.7	11.2	85.2
	3.00	511	14.2	14.8	100.0
	Total	3445	95.9	100.0	
Missing	System	146	4.1		
Total		3591	100.0		

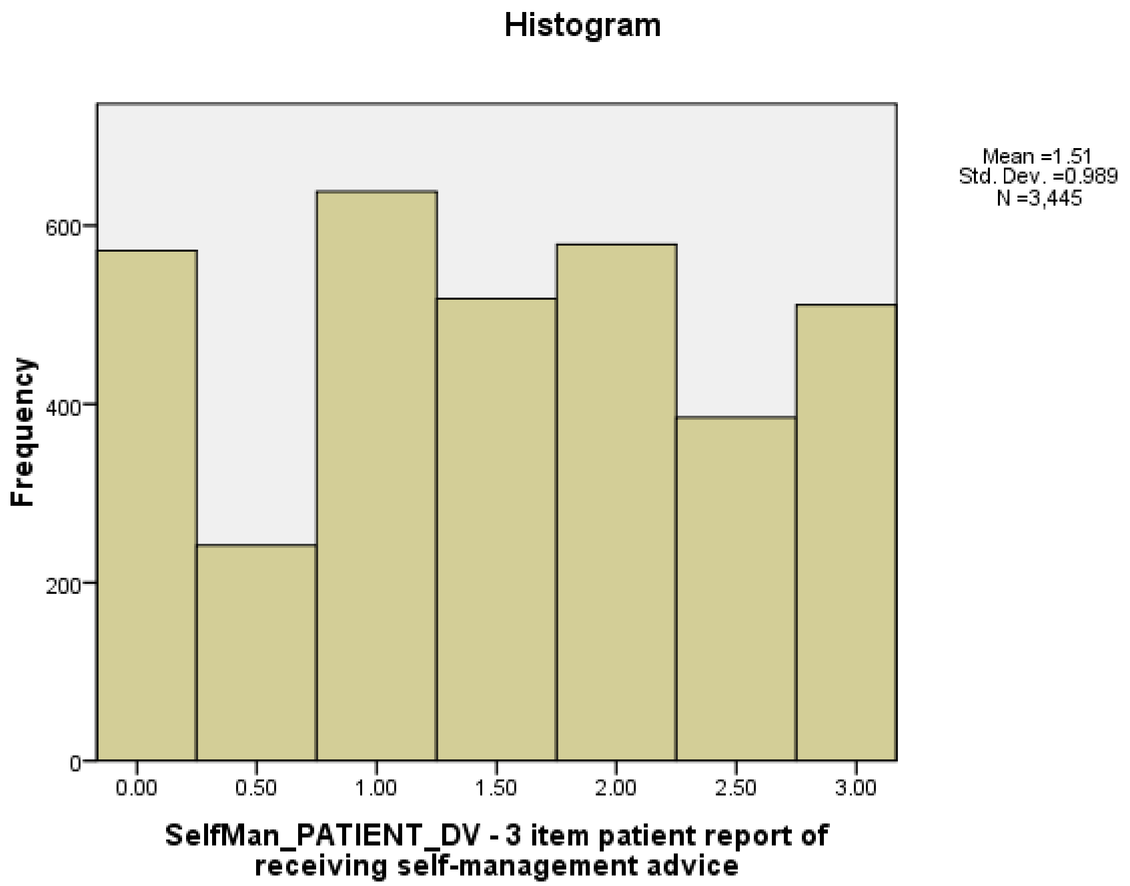
Histogram
Mean =1.51
Std. Dev. =0.989
N =3,445
Frequency
0
200
400
600
0.00
0.50
1.00
1.50
2.00
2.50
3.00
SelfMan_PATIENT_DV - 3 item patient report of receiving self-management advice

Providing general education *Single item*

q9l_0_1

		Frequency	Percent	Valid Percent	Cumulative Percent
Valid	.00	914	25.5	27.2	27.2
	1.00	2443	68.0	72.8	100.0
	Total	3357	93.5	100.0	
Missing	System	234	6.5		
Total		3591	100.0		

Providing general education *Complex DV (18 items)*

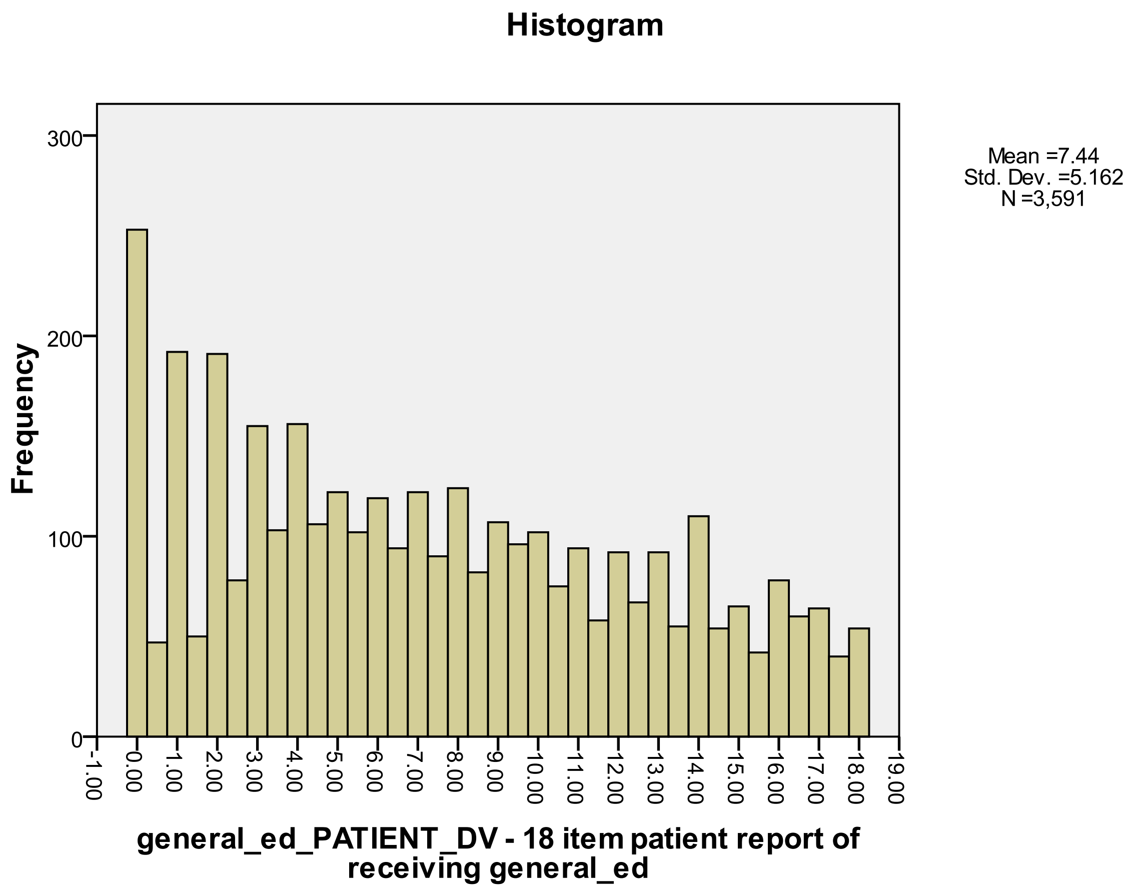

general_ed_PATIENT_DV - 18 item patient report of receiving general_ed

		Frequency	Percent	Valid Percent	Cumulative Percent
Valid	.00	253	7.0	7.0	7.0
	.50	47	1.3	1.3	8.4
	1.00	192	5.3	5.3	13.7
	1.50	50	1.4	1.4	15.1
	2.00	191	5.3	5.3	20.4
	2.50	78	2.2	2.2	22.6
	3.00	155	4.3	4.3	26.9
	3.50	103	2.9	2.9	29.8
	4.00	156	4.3	4.3	34.1
	4.50	106	3.0	3.0	37.1
	5.00	122	3.4	3.4	40.5
	5.50	102	2.8	2.8	43.3
	6.00	119	3.3	3.3	46.6
	6.50	94	2.6	2.6	49.2
	7.00	122	3.4	3.4	52.6
	7.50	90	2.5	2.5	55.1
	8.00	124	3.5	3.5	58.6
	8.50	82	2.3	2.3	60.9
	9.00	107	3.0	3.0	63.9
	9.50	96	2.7	2.7	66.5
	10.00	102	2.8	2.8	69.4
	10.50	75	2.1	2.1	71.5
	11.00	94	2.6	2.6	74.1
	11.50	58	1.6	1.6	75.7
	12.00	92	2.6	2.6	78.3
	12.50	67	1.9	1.9	80.1
	13.00	92	2.6	2.6	82.7
	13.50	55	1.5	1.5	84.2
	14.00	110	3.1	3.1	87.3
	14.50	54	1.5	1.5	88.8
	15.00	65	1.8	1.8	90.6
	15.50	42	1.2	1.2	91.8

16.00	78	2.2	2.2	93.9
16.50	60	1.7	1.7	95.6
17.00	64	1.8	1.8	97.4
17.50	40	1.1	1.1	98.5
18.00	54	1.5	1.5	100.0
Total	3591	100.0	100.0	

Examining feet *Single item*

q11b_0_1: in last 12 months have you - had your bare feet examined

		Frequency	Percent	Valid Percent	Cumulative Percent
Valid	no	322	9.0	9.5	9.5
	yes	3078	85.7	90.5	100.0
	Total	3400	94.7	100.0	
Missing	System	191	5.3		
Total		3591	100.0		
